# A New Species of *Diaporthe*: Metabolite Profile and Ability to Suppress Sclerotium Rot of Sunchoke

**DOI:** 10.3390/jof12100794

**Published:** 2026-10-21

**Authors:** Thanapat Suebrasri, Thanawan Gateta, Wasan Seemakram, Panupon Hongpakdee, Poramate Banterng, Urachart Kokaew, Sophon Boonlue

**Affiliations:** 1 Division of Basic and Preclinical Science, Institute of Science and General Education, Nakhon Ratchasima College, Nakhon Ratchasima 30000, Thailand; s.thanapat@nmc.ac.th; 2 Department of Microbiology, Faculty of Science, Khon Kaen University, Khon Kaen 40002, Thailand; thanawan.gateta@kkumail.com; 3 Department of Microbiology and Parasitology, Faculty of Medical Science, Naresuan University, Phitsanulok 65000, Thailand; wasans@nu.ac.th; 4 Department of Horticulture, Faculty of Agriculture, Khon Kaen University, Khon Kaen 40002, Thailand; panupon@kku.ac.th; 5 Department of Agronomy, Faculty of Agriculture, Khon Kaen University, Khon Kaen 40002, Thailand; bporam@kku.ac.th; 6 Plant Breeding Research Center for Sustainable Agriculture, Faculty of Agriculture, Khon Kaen University, Khon Kaen 40002, Thailand; 7 Applied Intelligence and Data Analytics Laboratory, College of Computing, Khon Kaen University, Khon Kaen 40002, Thailand; urachart@kku.ac.th

**Keywords:** antifungal activity, Diaporthaceae, endophytic fungi, Jerusalem artichoke, metabolomics, proteomics

## Abstract

The pathogenic fungus *Sclerotium rolfsii* is a major causal agent of tuber rot disease in Sunchoke (*Helianthus tuberosus*), resulting in significant yield losses. Therefore, this study aimed at investigating the antifungal activity of endophytic fungi isolated from medicinal plants against Sclerotium rot disease in sunchoke tubers. In addition, proteomic and metabolomic profiling of a selected endophytic fungus was performed. A total of 12 endophytic fungal isolates exhibiting antagonistic activity against *S. rolfsii* were obtained from 69 medicinal plant samples using the dual culture technique. Among these isolates, strain KKU-3.2, isolated from the root of *Cratoxylum formosum*, was selected and identified as a novel endophytic fungus, *Diaporthe formosum* sp. nov. Protein and metabolite profiling was performed using the selected strain *D. formosum* KKU-3.2. LC–MS-based proteomic analysis identified 32 proteins in the culture supernatant of the fungus. Furthermore, LC–MS-based metabolomic analysis detected 1600 metabolites, of which 24 were tentatively identified and selected as potential antifungal compounds based on previously reported activities or predicted functions. These tentatively identified metabolites were classified into eight chemical groups: coumarins, phenolic and organic acids, limonoids, depsides, pyrones and pyridines, flavonoids, sesquiterpenes, and indoles. Co-inoculation of *D. formosum* and *S. rolfsii* in sunchoke tubers demonstrated that *D. formosum* inhibited disease progression by 54.31%. This is the first report of *D. formosum* sp. nov. and, to the best of our knowledge, the first report describing the proteomic and metabolomic profiling of this novel species. The findings of this study provide a foundation for future research on the isolation and characterization of bioactive proteins and secondary metabolites, as well as their potential agricultural applications as biocontrol agents and biofertilizers.

## 1. Introduction

Sunchoke (*Helianthus tuberosus* L.), also known as Jerusalem artichoke, is an important tuber crop widely cultivated for its nutritional, medicinal, and industrial value. The tubers are rich in inulin, dietary fiber, and bioactive compounds, making sunchoke an economically important crop with increasing applications in food, pharmaceutical, and bioenergy industries [1]. However, sunchoke production is frequently threatened by various fungal diseases that negatively affect tuber quality, storage, and yield. Among soil-borne fungal pathogens, *Sclerotium rolfsii* is recognized as one of the most destructive phytopathogens causing tuber rot disease in sunchoke and many other economically important crops [2]. This pathogen can infect host tissues under favorable environmental conditions, leading to severe tissue decay, reduced productivity, and substantial economic losses. Effective management of *S. rolfsii* remains challenging due to its wide host range, persistence in soil, and ability to survive through resistant structures such as sclerotia [3].

Conventional disease management strategies mainly rely on chemical fungicides. Although fungicides are commonly used to suppress fungal pathogens, their long-term and excessive application has raised concerns regarding environmental pollution, the development of pathogen resistance, and potential adverse effects on human and animal health [4]. Therefore, environmentally friendly and sustainable alternatives for controlling plant pathogenic fungi are urgently needed.

Endophytic fungi are microorganisms that inhabit internal plant tissues without causing apparent harm to their host plants [5]. These fungi have gained considerable attention as valuable biological resources because they are capable of producing diverse bioactive compounds, extracellular enzymes, and secondary metabolites with antimicrobial, antioxidant, and plant growth-promoting properties [6,7,8]. Several endophytic fungi have been reported to exhibit antagonistic activity against phytopathogens through multiple mechanisms, including competition for nutrients and space, mycoparasitism, production of cell-wall-degrading enzymes such as chitinases and β-1,3-glucanases, and secretion of antifungal secondary metabolites. These mechanisms can act individually or in combination to restrict pathogen growth and colonization, highlighting the potential of endophytic fungi as promising biological control agents for sustainable plant disease management [9]. The genus *Diaporthe* comprises diverse endophytic fungi with considerable potential as biological control agents against plant pathogens. Their antagonistic activities may involve multiple mechanisms, including nutrient and space competition, antibiosis, and the production of antifungal secondary metabolites and extracellular enzymes [10]. Notably, *Diaporthe* species produce diverse bioactive metabolites, particularly polyketides, with demonstrated antifungal and antibacterial activities [11]. These properties highlight the potential of endophytic *Diaporthe* spp. for sustainable management of fungal diseases and as sources of novel bioactive compounds. However, *Diaporthe* species also include important plant pathogens, and their pathogenic potential should be considered when evaluating their use as biological control agents. For example, *Diaporthe helianthi* is a recognized pathogen of sunflower and has been associated with virulence through the action of fungal polyketide synthase genes [12]. The pathogenicity of newly identified *Diaporthe* species toward their target crops should be assessed before considering their potential application as biocontrol agents. Therefore, investigating novel *Diaporthe* species and their antagonistic mechanisms against important plant pathogens may provide promising candidates for the development of effective biocontrol agents. Although several *Diaporthe* species have demonstrated promising antifungal and biocontrol activities, their potential against *S. rolfsii*, particularly strains derived from medicinal plants, remains insufficiently explored. Previous studies have mainly focused on biological control efficacy based on growth inhibition or disease suppression assays, whereas the molecular and biochemical mechanisms underlying these interactions remain less well understood. Integrated metabolomic and proteomic approaches provide complementary information on bioactive secondary metabolites and proteins potentially involved in fungal antagonism. However, such approaches have rarely been combined to investigate the antifungal mechanisms of medicinal plant-derived endophytic fungi against *S. rolfsii*. Therefore, further investigation using integrated omics approaches is warranted to better elucidate the mechanisms underlying their biocontrol potential.

Several studies have reported the potential of endophytic fungi to inhibit *S. rolfsii* and reduce disease severity in sunchoke. For instance, the co-inoculation of *Trichoderma harzianum* and arbuscular mycorrhizal fungi (AMF) has been extensively investigated. The synergistic effects of cultivar, AMF, and *T. harzianum* in suppressing *S. rolfsii* may be attributed to competition for colonization within plant roots, while the increased development of extraradical hyphae enhances soil nutrient availability, thereby improving plant resistance or tolerance against the pathogen [13]. In addition, Suebrasri et al. reported that endophytic fungi isolated from the petioles of Jerusalem artichoke and ginger roots could suppress *S. rolfsii* through multiple mechanisms, including the production of chitinase and β-1,3-glucanase, which contribute to fungal cell wall degradation, as well as the secretion of secondary metabolites with antifungal activity [14]. Consequently, endophytic fungi are increasingly considered promising candidates for biological control in sustainable agriculture.

Medicinal plants represent an important reservoir of diverse endophytic microorganisms due to their rich phytochemical composition and ecological adaptations. Endophytic fungi associated with medicinal plants may possess unique biochemical characteristics and produce metabolites with biological activities [15]. However, relatively few studies have specifically investigated the antifungal potential of endophytic fungi isolated from medicinal plants against *S. rolfsii*, particularly in relation to their underlying antagonistic mechanisms. Although metabolomic and proteomic approaches have increasingly been applied to characterize fungal secondary metabolites and proteins associated with biological activities, their integrated application to investigate the mechanisms underlying the antagonistic activity of medicinal plant-derived endophytic fungi against *S. rolfsii* remains less frequently reported [13,14]. This knowledge gap provides a rationale for combining biological assays with proteomic and metabolomic analyses to gain a more comprehensive understanding of the potential mechanisms involved in pathogen suppression. Therefore, this study aimed at investigating the antifungal activity of endophytic fungi isolated from medicinal plants against *S. rolfsii*, the causal agent of sunchoke tuber rot disease. In addition, proteomic and metabolomic profiling of a selected endophytic novel *Diaporthe* species was performed to identify potential bioactive proteins and secondary metabolites associated with antifungal activity. The findings of this study may contribute to the development of novel biological control agents for sustainable agricultural applications.

## 2. Materials and Methods

### 2.1. Collection and Plant Materials

A total of sixty-nine medicinal plant samples representing the plant species listed in Supplementary Data Table S1 were collected from a mountainous area in Loa Hai Ngam Subdistrict, Kuchinarai District, Kalasin Province, Thailand (16°47′47.64″ N, 104°23′28.00″ E). The collected plant materials were stored in sterilized plastic bags at 4 °C until further processing.

### 2.2. Isolation of Endophytic Fungi

Plant samples were washed with tap water to remove surface debris and cut into 1–2 mm segments. Surface disinfection was performed by immersing the segments in 70% (*v*/*v*) ethanol for 3 min, then in 5% (*v*/*v*) sodium hypochlorite for 1 min, and finally in 70% (*v*/*v*) ethanol for 3 min. The segments were then rinsed three times with sterile distilled water and dried on sterile paper under airflow. Disinfected samples were placed on half-strength potato dextrose agar (PDA) supplemented with 2000 mg/L chlortetracycline to suppress endophytic bacterial growth. Emerging fungal colonies were subcultured onto fresh PDA plates until pure cultures were obtained. To confirm surface sterilization efficacy, an aliquot of the final sterilizing solution was inoculated onto agar plates and incubated for 2 weeks to verify the absence of fungal contamination [6].

### 2.3. Screening Endophytic Fungi as Biological Control Agents Against S. rolfsii

All endophytic fungal isolates were screened for antagonistic activity against *Sclerotium rolfsii* using a dual culture assay, following the methods of Suebrasri et al. (2020) [14]. Mycelial plugs (5 mm diameter) from 7-day-old endophytic fungal cultures grown on potato dextrose agar (PDA) were placed on fresh PDA plates at a distance of 2 cm from one edge, opposite to a mycelial plug of *S. rolfsii*. Plates inoculated with *S. rolfsii* alone served as the control. All plates were incubated at 30 °C in the dark for 5 days. After incubation, the radial growth of *S. rolfsii* was measured on dual culture plates (R2) and control plates (R1). The percentage inhibition of radial growth (PIRG) was calculated according to Suebrasri et al. (2020) using the following formula: PIRG (%) = [(R1 − R2)] × 100 [14].

### 2.4. Fungal Endophyte Identification

Fungal endophyte isolates exhibiting antagonistic activity against *S. rolfsii* were preliminarily identified based on light-microscopy observations of morphological characteristics and colony morphology on potato dextrose agar (PDA). Molecular identification was subsequently performed by amplifying the internal transcribed spacer (ITS1–4) regions, β-tubulin 2 (TUB2), and translation elongation factor 1-alpha (TEF-1α) genes using universal primers. The primer sequences used were ITS1 (5′-TCC GTA GGT GAA CCT GCG G-3′), ITS4 (5′-TCC TCC GCT TAT TGA TAT GC-3′), Btub2 (5′-GTB CAC CTY CAR ACC GGY CAR TG-3′), Btub4 (5′-CCR GAY TGR CCR AAR ACR AAG TTG TC-3′), EF1-278F (5′-CAT CGA GAA GTT CGA CAA GG-3′), and EF1-986R (5′-TAC TTG AAG GAA CCC TTA CC-3′). PCR amplification was carried out in a total reaction volume of 25 µL containing 5 µL of 10× PCR buffer, 1.5 µL of 25 mM MgCl_2_, 0.5 µL of 1.25 mM dNTPs, 1.5 µL of each primer (150 µM), 100 ng of fungal DNA template, and 0.2 µL of Taq DNA polymerase (5 U/µL) (Thermo Fisher Scientific, Vilnius, Lithuania), with sterile distilled water added to the final volume. Amplification was performed using a thermal cycler under the following conditions: initial denaturation at 95 °C for 5 min; 35 cycles of denaturation at 95 °C for 1 min, annealing at 52 °C for TEF-1α and ITS1–4 and at 55 °C for TUB2 for 1 min, and extension at 72 °C for 90 s; followed by a final extension at 72 °C for 10 min. PCR products were purified using the GeneJET™ PCR Purification Kit (Fermentas, Burlington, Canada) and sequenced using the 1st BASE DNA sequencing service (Seri Kembangan, Selangor, Malaysia). The obtained nucleotide sequences were analyzed using the online NCBI BLAST tool (National Center for Biotechnology Information, Bethesda, MD, USA) for fungal identification.

Authentic DNA sequences retrieved from GenBank, together with sequences obtained from PCR products and newly generated sequences from this study, were compiled for phylogenetic analysis (Table 1). *Diaporthe corylina* CBS 121124 was used as the outgroup taxon. Multiple sequence alignment was performed using MUSCLE [16] with default parameters, and manual adjustments were made when necessary, using BioEdit version 6.0.7 [17]. The combined alignment of ITS, TEF-1α, and β-tubulin (TUB2) sequences was subsequently deposited in TreeBASE.

Phylogenetic analysis was performed using a combined dataset of ITS, TEF-1α, and β-tubulin (TUB2) sequences; the sequence alignment file is provided in Supplementary Data File S1. Phylogenetic trees were constructed using both maximum likelihood (ML) and Bayesian inference (BI) methods. ML analysis was conducted under the GTRCAT nucleotide substitution model with 25 rate categories and 1000 bootstrap (BS) replicates using RAxML-HPC2 version 8.2.12 [18,19] through the CIPRES web portal. For BI analysis, the best-fit substitution models were selected based on the Akaike information criterion (AIC) in jModelTest version 2.1.10 [20], with GTR applied to ITS and β-tubulin datasets. Bayesian analysis was performed using MrBayes version 3.2.6 [21] with six simultaneous Markov chains run for 1,000,000 generations, starting from random trees and sampling every 1000 generations. The initial 20% of sampled trees were discarded as burn-in, and the remaining trees were used to estimate posterior probabilities (PPs) in a majority-rule consensus tree. The resulting phylogenetic trees were visualized using FixTree version 1.4.0 [22].

### 2.5. Culture Conditions and Proteomic LC-MS-Based Analysis

The endophytic fungus KKU-3.2 was cultured in potato dextrose broth (PDB) with shaking at 250 rpm at 25 °C for 2 weeks. The fermentation broth was then collected and freeze-dried using a lyophilizer. For sample preparation, the dried broth was resuspended in 3 mL of distilled water and sonicated for 20 min. The samples were subsequently centrifuged at 14,000 rpm for 10 min, and the supernatants were collected. Proteins were precipitated by adding 10 mL of 100% methanol to each supernatant, followed by thorough mixing and centrifugation at 14,000 rpm for 10 min. The resulting protein pellets were collected, air-dried for 10 min, and resuspended in 200 µL of 50 mM ammonium bicarbonate (ABC) buffer (pH 7.5). The protein concentration was determined using the Bradford assay [23], and the total protein concentration of the KKU-3.2 sample was 0.21 mg/mL. An aliquot of 40 µL of the protein solution was reduced with 10 mM dithiothreitol (DTT) at 65 °C for 30 min. Alkylation was subsequently performed by adding 25 mM iodoacetamide (IAA) and incubating the mixture at room temperature in the dark for 20 min. Protein digestion was performed by adding 2.5 µL of trypsin solution (0.1 mg/mL) and incubating the samples at 37 °C overnight. The digestion reaction was terminated by adding 10% formic acid (FA). The digested samples were centrifuged at 14,000 rpm for 10 min, and the resulting supernatants were collected and transferred to LC–MS vials for subsequent analysis.

LC–MS/MS analysis was performed using an Agilent LC-QTOF 6545XT system (Agilent Technologies, Santa Clara, CA, USA) operated in positive electrospray ionization mode. Peptides were separated using an Agilent Peptide Map column (2.1 × 150 mm, 2.7 µm) maintained at 60 °C. A 20 µL sample was injected, and chromatographic separation was performed over an 85 min run at a flow rate of 0.4 mL/min. The mobile phases consisted of water containing 0.1% formic acid (solvent A) and acetonitrile containing 0.1% formic acid (solvent B), using a gradient elution program. Mass spectra were acquired in MS/MS centroid mode over an m/z range of 100–1700 for MS scans and 50–1700 for MS/MS scans. The MS source conditions were as follows: gas temperature, 325 °C; drying gas flow rate, 13 L/min; nebulizer pressure, 35 psi; capillary voltage, 4000 V; and nozzle voltage, 500 V. Collision energies were applied according to the precursor charge state using the following equations: 3.1 × (m/z/100) + 1 for +1 and +2 ions and 3.6 × (m/z/100) − 4.8 for ions with charge states ≥+3. The reference mass used for internal calibration was m/z 922.0098. For proteomic data processing, the Agilent *.d files were converted to mzXML files using MSConvert and OpenMS. Protein identification was performed using MaxQuant software version 2.6.3 against the UniProt database for *Diaporthe* (taxonomy ID: 36922). Trypsin was specified as the digestion enzyme. Carbamidomethylation of cysteine was set as a fixed modification, whereas N-terminal acetylation and methionine oxidation were set as variable modifications. The LFQ minimum ratio count was set to 1, and the protein and peptide-spectrum-match false discovery rate (FDR) was intentionally set to 0.1 to increase the number of proteins identified. Proteins with q-values < 0.01 were considered high-confidence identifications, whereas those with q-values between 0.01 and 0.1 were considered moderate-confidence identifications. The identified proteins, together with the number of peptides supporting each protein and their corresponding q-values, are presented in Supplementary Data Table S3.

### 2.6. Metabolite Extraction and LC-MS Analysis

Fungal endophytes were cultured on potato dextrose agar (PDA) at 30 °C for 7 days. Mycelial plugs (0.5 cm diameter; cork borer no. 5) from 250 colonies were inoculated into 5000 mL Erlenmeyer flasks containing 2500 mL of potato dextrose broth (PDB) and incubated at room temperature under static conditions for 30 days. After incubation, the cultures were filtered to separate the mycelium from the broth. The mycelial biomass was dried in a hot air oven at 55 °C for 5 days. The filtered broth and dried mycelium were each extracted twice with an equal volume of ethyl acetate. The organic phases were concentrated to dryness at 40 °C under reduced pressure using a rotary vacuum evaporator, and the crude fungal extracts were weighed. Each crude extract was dissolved in dimethyl sulfoxide (DMSO) and diluted with sterile distilled water to a final concentration of 20 mg/mL. Antifungal activity against *S. rolfsii* was evaluated using the disc diffusion method [6]. The crude fungal extracts of the endophytic fungus KKU-3.2 were used to investigate metabolic profiling.

Regarding sample preparation for LC-MS-based analysis [24,25], the crude fungal extracted was weighed and mixed with 70% (*v*/*v*) ethanol, supplied with 50 ng/mL of sulfadimethoxine (as internal standard), to a final concentration of 10 mg/mL. Then, the sample was centrifuged at 14,000 rpm for 10 min, and the supernatant was collected, transferred to a vial and subjected to LC-MS analysis. Liquid chromatography (LC) analysis was performed using a Poroshell 120 EC-C18 column (2.1 × 100 mm, 2.7 µm) maintained at 50 °C. The injection volume was 10 µL, and the flow rate was set at 0.4 mL/min. The mobile phases consisted of 0.1% formic acid in water (solvent A) and 0.1% formic acid in acetonitrile (solvent B). Separation was carried out using a gradient elution program under the following LC conditions. Mass spectrometry (MS) analysis was performed using an Agilent LC-QTOF 6545XT system operated in high-resolution mode under both positive and negative electrospray ionization conditions. The drying gas temperature and flow rate were set at 325 °C and 13 L/min, respectively, while the sheath gas temperature and flow rate were maintained at 275 °C and 12 L/min. The nebulizer pressure was set at 45 psi. Capillary voltages were 4000 V in positive mode and 3000 V in negative mode. The isotope model width was set at 1.3 m/z. MS1 spectra were acquired over a mass range of 40–1700 m/z, whereas MS2 spectra were collected over a mass range of 25–1000 m/z. Collision energies were set at 20 eV for the positive mode and 10 eV for the negative mode. Data acquisition was performed at a rate of 3.35 spectra/s, with a maximum of 10 precursors per cycle and a precursor threshold of 5000 counts. The retention time threshold was set at 0.001%. Reference masses used for mass calibration were m/z 121.0509 and 922.0098 in positive mode, and m/z 112.9856 and 1033.9881 in negative mode.

### 2.7. Pathogenicity Test and Inhibition of Disease Expression

The pathogenicity test was conducted on healthy sunchoke tubers (Hel65 strain). The fungi *D. formosum* and *S. rolfsii* were cultured on PDA for 7 days prior to inoculation. The sunchoke tubers were washed under running tap water and surface-sterilized with 70% (*v*/*v*) ethanol. Subsequently, the tubers were wounded twice along the line connecting both ends using a cork borer (No. 0.5) to a depth of 0.5 cm. On each tuber, one wound was inoculated with the endophytic fungus *D. formosum*, while the other wound was inoculated with *S. rolfsii*. The positive control was inoculated with *S. rolfsii* only, whereas the negative control was inoculated with a sterile agar plug. Each tuber was placed in a sterilized bottle containing cotton moistened with distilled water to maintain humidity and then incubated at 25 °C for 7 days, with the experiment performed in five replicates. After incubation, the mean lesion size was measured at each wound site on the sunchoke tubers. The lesion area was determined by measuring the maximum width and length of the necrotic tissue and calculating the area as length Î width. The lesion area was expressed in square millimeters (mm^2^). Moreover, the percentage of disease reduction was calculated according to the following formula:

The disease reduction (%) = [(C − T)/C] × 100

where: C (mm^2^) = lesion area caused by *S. rolfsii* in the positive control; T (mm^2^) = lesion area at the *S. rolfsii* inoculation site on sunchoke tubers co-inoculated with the endophytic fungus *D. formosum* and *S. rolfsii* [26].

### 2.8. Statistical Analysis

Statistical analysis was performed using an independent *t*-test in SPSS software version 26.0 (IBM Corp., Armonk, NY, USA) to determine significant differences between treatments. Differences were considered statistically significant at *p* < 0.05.

## 3. Results

### 3.1. Isolation and Screening of the Effects of Fungal Endophytes on Mycelial Growth of S. rolfsii

**Figure 1:**
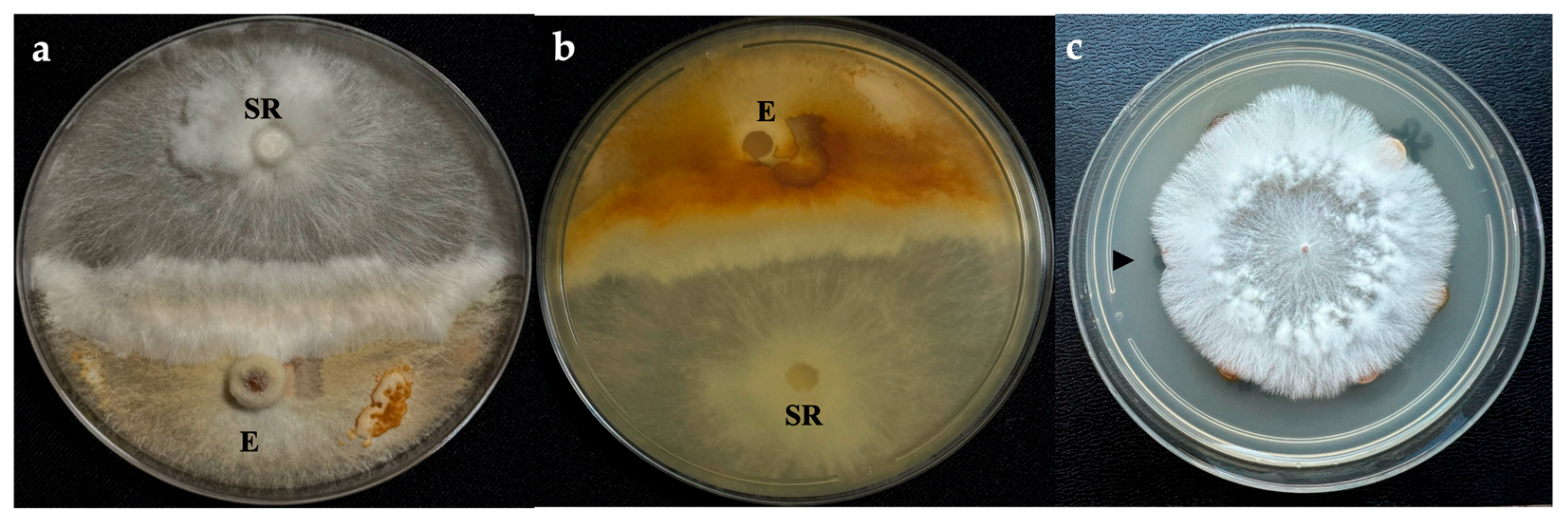
Dual culture assay showing the antifungal activity of endophytic fungal isolate KKU-3.2 (E) against *S. rolfsii* (SR). (**a**) Top view; (**b**) Reverse view; (**c**) Effect of crude ethyl acetate extract of endophytic fungal isolate KKU-3.2 against *S. rolfsii* (black arrowhead).

A total of 69 medicinal plant samples were collected from a mountain area in Loa Hai Ngam Subdistrict, Kuchinarai District, Kalasin Province, Thailand, for the isolation of endophytic fungi and evaluation of their antagonistic activity against the pathogenic fungus *S. rolfsii*. A total of 196 endophytic fungal isolates were recovered from root, leaf, and stem tissues, of which 12 isolates (6.12%) exhibited inhibitory activity against *S. rolfsii* in the dual-culture assay. The percentage inhibition among the antagonistic isolates ranged from 50.00% to 68.57% (Supplementary Data Table S2). Among these isolates, endophytic fungal isolate KKU-3.2 exhibited 57.14% inhibition against *S. rolfsii* (Figure 1a,b).

### 3.2. Biological Activity of Crude Extracts from Fungal Endophytes

The 12 antagonistic endophytic fungal isolates were identified based on their ITS gene sequences, as shown in Supplementary Table S2. Among these isolates, KKU-3.2, isolated from the root of the host plant *Cratoxylum formosum*, was identified as a novel *Diaporthe* species and was therefore selected for further study. The ethyl acetate crude extracts obtained from the mycelia and fermentation broths of the 12 isolates were subsequently evaluated for antagonistic activity against *S. rolfsii*. Most of the crude extracts showed no inhibitory effect, whereas the crude extract of KKU-3.2 exhibited slight inhibitory activity against *S. rolfsii* (Figure 1c). KKU-3.2 was subsequently selected for further proteomic and metabolomic profiling.

### 3.3. Identification of Fungal Endophyte

**Figure 2:**
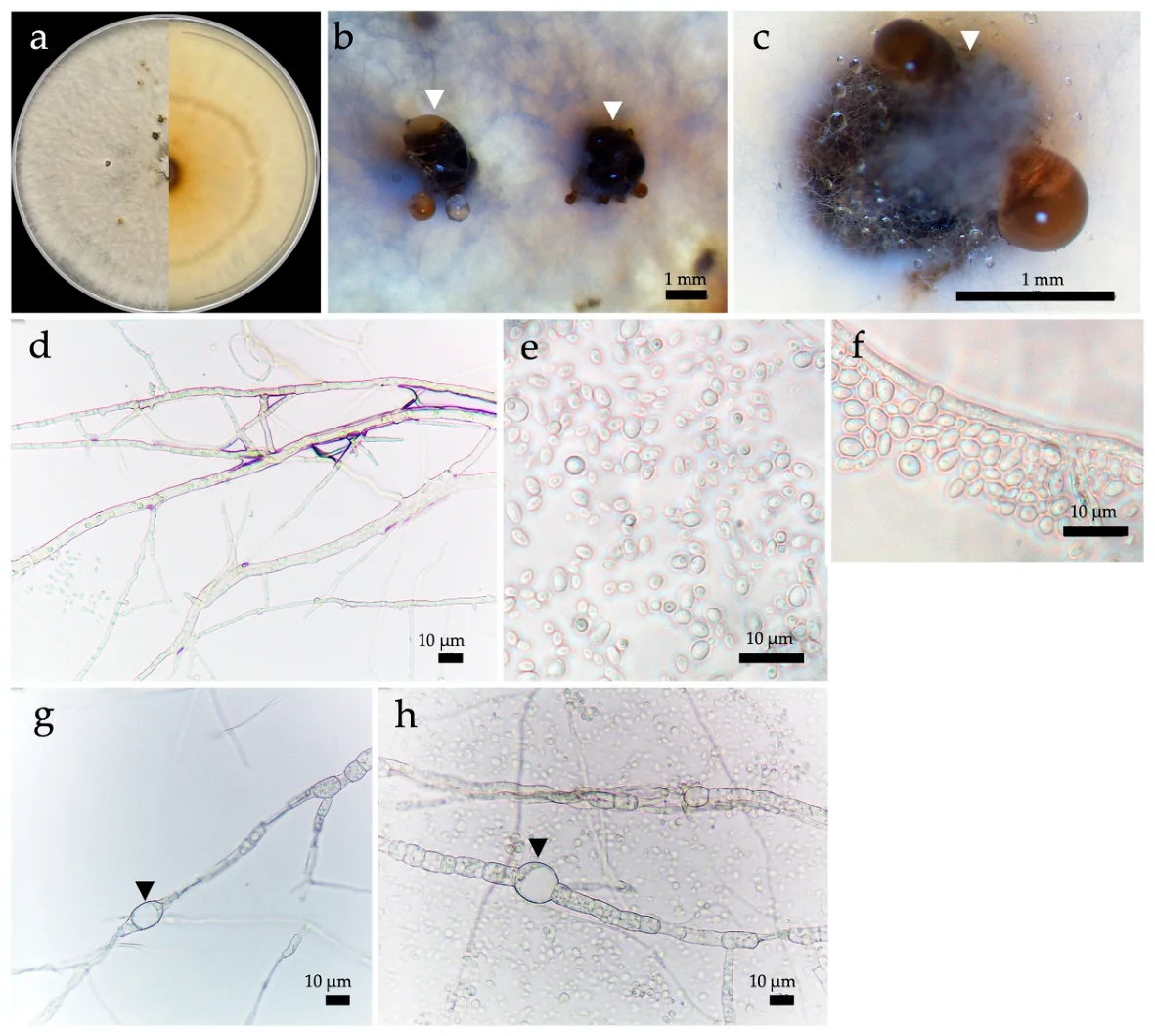
Morphological characteristics of endophytic fungal isolate 3.2. (**a**) Colony appearance on potato dextrose agar (PDA): upper view (left) and reverse view of the colony (right); (**b**,**c**) conidiomata produced on PDA (white arrows); (**d**) hyphal morphology; (**e**,**f**) alpha conidia; (**g**,**h**) chlamydospore-like structures (black arrows).

The endophytic fungal isolate KKU-3.2 was cultured on potato dextrose agar (PDA) for 10 days. The colonies were flat and spreading, with white flocculent aerial mycelium and entire margins. Black conidiomata were observed on the mycelial surface, while the reverse side of the colony exhibited a concentric ring pattern (Figure 2a). Conidiomata formation was clearly observed on PDA cultures (Figure 2b,c). The hyphae were septate (Figure 2d), and alpha conidia were abundant, unicellular, ellipsoidal to cylindrical, hyaline, aseptate (Figure 2e,f). Chlamydospore-like structures were also observed (Figure 2g,h). The sizes of the alpha conidia of *D. formosum* sp. nov. compared with those of other *Diaporthe* species are presented in Table 2. The results of BLAST sequence similarity analyses for the ITS, *TEF-1α*, and β-tubulin (TUB2) regions are presented in Table 3. Comparison of the ITS, *TEF-1α*, and TUB2 sequences of endophytic fungal isolate KKU-3.2 with those of reference strains revealed that the ITS sequence similarities with the top five reference strains were lower than 98.55%. Moreover, the sequence comparisons revealed genetic divergence from the closest related species, with sequence similarity values for the TEF-1α and TUB2 genes below 96.49% and 96.61%, respectively. Based on these results, the endophytic fungal isolate KKU-3.2 is likely to represent a novel species. Therefore, its phylogenetic placement was further investigated using multigene phylogenetic analyses.

Taxonomy

*Diaporthe formosum* Suebrasri T. and Seemakram W sp. nov. (Figure 2.)

MycoBank: MB865277

Cultural characteristics: After 10 days of culture on PDA in the dark at 25 °C, the colony was flat and spreading, with abundant white, flocculent aerial mycelia and entire margins, whereas the reverse exhibited distinct concentric rings.

Description: Conidiomata, black conidiomata developed on the colony surface. Alpha conidia were abundant, unicellular, ellipsoidal to cylindrical, hyaline, aseptate; size, 3.5–5.78 × 1.64–2.66 μm (mean = 4.77 × 2.07 μm, n = 30).

Etymology: *formosum*, referring to the epithet of the host plant *Cratoxylum formosum*

Type: Thailand, Kalasin Province, Kuchinarai District, a mountain area in Lao Hai Ngam Subdistrict (16°47′47.64″ N, 104°23′28.00″ E), isolated as an endophytic fungus from the root of *Cratoxylum formosum*, 27 April 2025; collectors: Thanapat Suebrasri and Wasan Seemakram.

Holotype: TISTR 3739, a lyophilized (metabolically inactive) culture deposited at the Thailand Institute of Scientific and Technological Research (TISTR); Ex-type culture KKU-3.2 (preserved as a living culture on PDA at the Mycotechnology laboratory, Department of Microbiology, Faculty of Science, Khon Kaen University, Thailand). GenBank accession numbers for ITS: PZ203759; *TEF-1α*: PZ399302; and TUB2: PZ435428.

Habitat: The only known host is the plant *C. formosum,* The fungus was isolated from the root of *C. formosum,* the only known host.

Note: The strain of *D. formosum* sp. nov. was compared with the typical characteristics of known species (Table 2), *D. formosum* sp. nov. differs from *D. aseana* MFLUCC 12-0299a, *D. pseudophoenicicola* CBS 462.69 and *D. australiana* BRIP 66145 in that it possesses smaller alpha conidia (3.5–5.78 × 1.64–2.66 μm vs. 5–8.5 × 1.5–2 μm, 5–6.5(–7) × 2.3–3.0 μm and 6–9 × 2–3 µm) Therefore, the morphological characteristics and molecular phylogenetic results support *D. formosum* as a new species.

**Figure 3:**
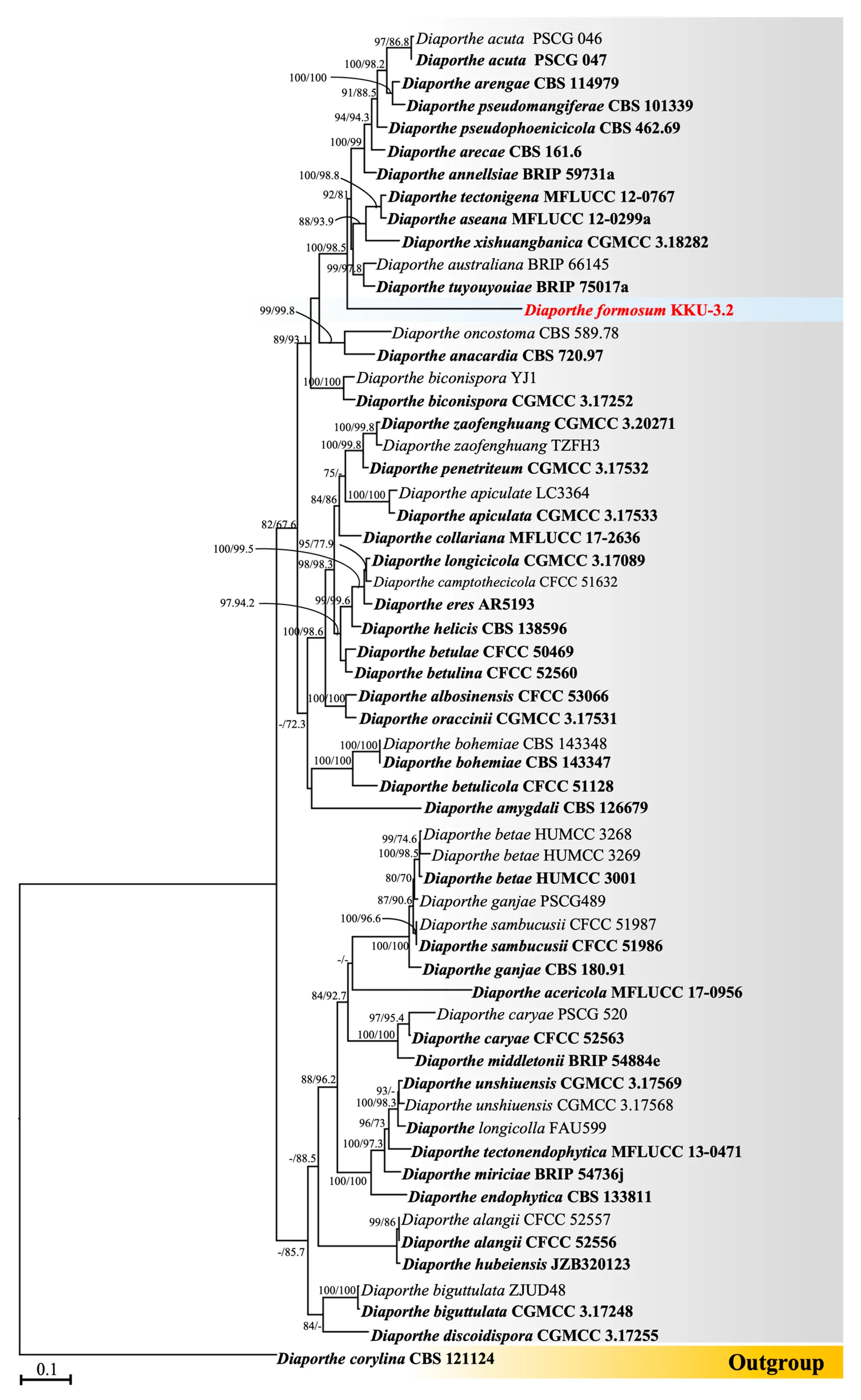
Phylogenetic tree derived from maximum likelihood analysis of 60 fungal strains harboring combined ITS, *Tef-1α*, and Tub2 genes. *D. corylina* CBS 121124 was used as the outgroup. The numbers above the branches represent the maximum likelihood bootstrap percentages. Bootstrap values ≥ 70% and SH-aLRT. The scale bar represents the expected number of nucleotide substitutions per site. The sequences of the fungal strains obtained in this study are in red.

The phylogenetic tree generated from the maximum likelihood (ML) analysis is shown in Figure 3. The combined sequence alignment comprised 60 strains, with *Diaporthe corylina* CBS 121124 used as the outgroup taxon. The dataset consisted of 1368 characters, including alignment gaps (511 for ITS, 511 for TUB2, and 346 for TEF-1α). The phylogenetic tree placed endophytic fungal strain KKU-3.2 in a distinct clade, clearly separated from previously described *Diaporthe* species, with moderate-to-strong branch support (BS = 92%, SH-aLRT = 81%). Based on these phylogenetic results, strain KKU-3.2 was recognized as a novel species and is herein described as *Diaporthe formosum* KKU-3.2 sp. nov. The accession numbers for the ITS, TEF-1α, and TUB2 sequences were PZ203759, PZ399302, and PZ435428, respectively (Table 1).

### 3.4. LC-MS-Based Proteomic Profiling

**Figure 4:**
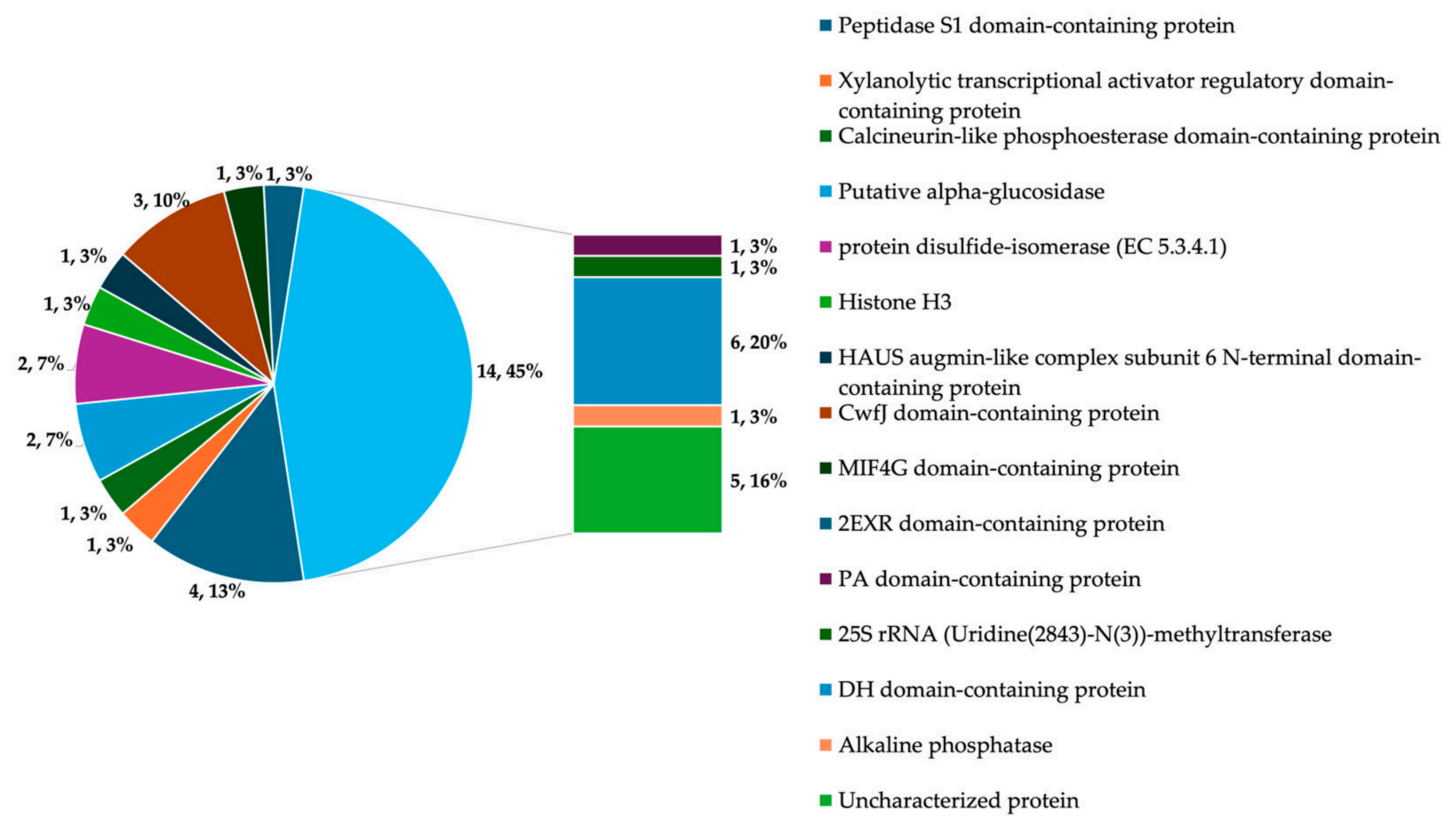
Proteomic profile of *Diaporthe formosum* sp. nov.

The secreted proteome of the endophytic fungus *D. formosum* KKU-3.2 was analyzed by liquid chromatography-mass spectrometry (LC-MS). In the present study, proteomic analysis identified a total of 32 proteins in the culture medium supernatant of the fungus (Supplementary Data Table S3). Among these proteins, five proteins could not be identified as a type of protein (Figure 4). Several fungal proteins were identified, including peptidase and alpha glucosidase, which are involved in the hydrolysis of proteins and α-glycosidic bonds in carbohydrates such as starch and glycogen. Moreover, the HAUS augmin-like complex subunit 6 N-terminal domain-containing protein is associated with microtubule rearrangement during cell division, thereby potentially affecting fungal growth inhibition.

### 3.5. LC-MS-Based Metabolic Profiling and Identification of Antifungal Secondary Metabolites from D. formosum sp. nov.

Metabolic profiling of the endophytic fungus *D. formosum* KKU-3.2 detected approximately 1600 metabolites (Supplementary Data Table S4). Among these, 24 metabolites were tentatively annotated and considered potential antifungal compounds based on their previously reported activities and predicted functions (Table 4). These metabolites belonged to several chemical classes, primarily coumarins, phenolic acids, limonoids, and depsides. The reported or predicted antifungal activities of these compounds involve diverse mechanisms, including disruption of cellular integrity and interference with essential metabolic pathways. However, these activities and mechanisms were not experimentally validated for the identified metabolites in the present *D. formosum* KKU-3.2–*S. rolfsii* system. For example, kojic acid and various coumarin derivatives have been reported to exhibit biological activities such as tyrosinase inhibition and induction of oxidative stress, respectively, suggesting their potential relevance to antifungal activity. Furthermore, organic acids such as propionic acid contributed to growth inhibition by acidifying the fungal cytoplasm, leading to metabolic exhaustion. Therefore, the potent inhibition of *S. rolfsii* observed in this study may be supposed to be due to the synergistic effects of the 24 tentatively identified secondary metabolites produced by *D. formosum* KKU-3.2.

### 3.6. Pathogenicity and Biocontrol Efficacy of D. formosum KKU-3.2

**Figure 5:**
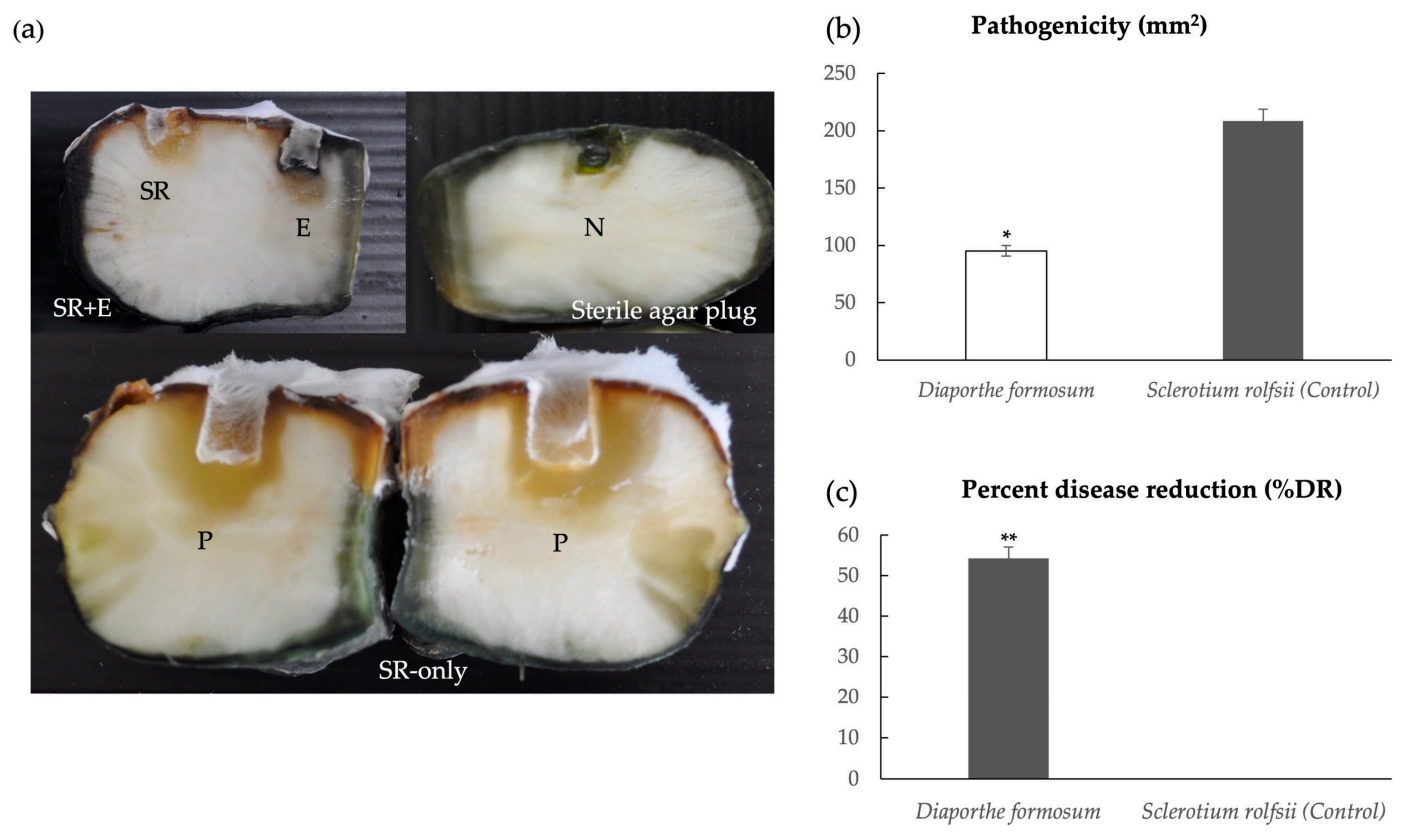
Effect on sunchoke tuber using co-inoculation between the fungal endophytes (E) and pathogenic fungus *S. rolfsii* (SR), positive control pathogenic fungus *S. rolfsii* inoculated in sunchoke tuber (P), and negative control only sterilized agar plug placed on sunchoke tuber (N) after 7 days of incubation (**a**); pathogenicity (**b**); and percent inhibition of disease progression in sunchoke (**c**). Error bars are SDs of five replicates, and asterisks indicate significant differences by t-*test* at * *p* < 0.05 and ** *p* < 0.01.

To evaluate the virulence and biocontrol potential of the endophytic fungus *D. formosum* KKU-3.2, pathogenicity tests were conducted on sunchoke tubers. Furthermore, the interaction between *D. formosum* KKU-3.2 and the pathogenic fungus *S. rolfsii* was assessed through co-inoculation to determine the endophyte’s inhibitory effects. In the pathogenicity assay, tubers inoculated solely with the endophyte *D. formosum* KKU-3.2 exhibited minimal disease progression (Figure 5a). However, in the co-inoculation treatment, *D. formosum* KKU-3.2 significantly suppressed the virulence of *S. rolfsii*. The resulting lesion area in co-inoculated tubers was 95.25 mm^2^, which was significantly lower than the 208.50 mm^2^ observed in the positive control (inoculated with *S. rolfsii* alone) (*p* < 0.05; Figure 5b). Analysis of the interaction between the fungal endophyte and the pathogen revealed a mean disease reduction of 54.31% (Figure 5c). These results demonstrate that the endophytic fungus *D. formosum* KKU-3.2 effectively inhibits the progression of Sclerotium rot in sunchoke tubers.

## 4. Discussion

This study demonstrated that the novel endophytic fungus *D. formosum* KKU-3.2 exhibited effective antagonistic activity against *S. rolfsii*, as confirmed by the dual culture assay, with an inhibition rate of 57.14%. Based on morphological characteristics [26,27,28] and phylogenetic analysis of ITS rDNA, β-tubulin, and TEF-1α gene sequences, this isolate was identified as *D. formosum* KKU-3.2, a newly described species within the genus *Diaporthe*. Endophytic fungi are widely recognized for suppressing phytopathogens through multiple mechanisms, including mycoparasitism, antibiosis, nutrient competition, secretion of secondary metabolites, and induction of plant stress resistance [28]. However, knowledge regarding the antifungal potential of *Diaporthe* species against *S. rolfsii* remains limited. In the present study, the stronger inhibition observed in the dual-culture assay compared with the ethyl acetate crude extract assay may be attributed to multiple antagonistic mechanisms. In addition to the production of antifungal secondary metabolites and extracellular enzymes, *D. formosum* KKU-3.2 may suppress *S. rolfsii* through competition for nutrients and space. This observation is consistent with previous findings for *Diaporthe phaseolorum*, in which antagonistic activity against *S. rolfsii* in sunchoke was primarily associated with hyphal competition and nutrient limitation. Thus, the combined effects of these mechanisms may account for the greater inhibition observed in the dual-culture assay than in the crude extract assay [14]. Proteomic analysis identified 32 potential secreted proteins in *D. formosum* KKU-3.2, which were classified into six functional groups. Among these, proteins associated with cell wall and protein degradation, including peptidase S1 domain-containing proteins and alkaline phosphatase, may be associated with fungal antagonistic potential. Putative alpha-glucosidase is primarily involved in the hydrolysis of α-glycosidic bonds in carbohydrates such as starch and glycogen. Based on their annotated functions and previous reports, these extracellular proteins may be involved in processes related to the degradation of fungal structural components and carbohydrate metabolism [35,52,53]. However, their specific roles in the antagonistic interaction between *D. formosum* KKU-3.2 and *S. rolfsii* were not experimentally validated in the present study. In addition to extracellular proteins, metabolomic profiling revealed diverse secondary metabolites that were tentatively annotated and may have potential antifungal activities based on previous reports. These metabolites were classified into eight major chemical groups, namely coumarins, phenolic and organic acids, limonoids, depsides, pyrones and pyridines, flavonoids, sesquiterpene lactones, and indoles. The presence of these compounds suggests that the antagonistic potential of *D. formosum* KKU-3.2 may involve biochemical interactions in addition to nutrient competition. Previous studies have reported that coumarins and phenolic acids may affect fungal membranes, oxidative stress, and metabolic processes [30,31,34]. Similarly, limonoids, depsides, and catechol derivatives have been reported to exhibit activities associated with membrane destabilization, lipid peroxidation, and cellular damage [37,38,40]. Pyrones, pyridines, and flavonoids have also been reported to possess various biological activities, including metal chelation, enzyme inhibition, oxidative stress induction, and membrane perturbation. Nevertheless, these activities were not directly demonstrated for the tentatively annotated metabolites in the present *D. formosum* KKU-3.2–*S. rolfsii* system [45,46,47]. Furthermore, (+)-costunolide and indole-3-acetaldehyde may interfere with fungal signaling, ROS balance, and intracellular homeostasis, which could further suppress pathogen colonization [50,51]. These findings are consistent with previous studies demonstrating that endophytic fungi can effectively suppress *S. rolfsii* in sunchoke. Suebrasri et al. reported that endophytic fungi isolated from Jerusalem artichoke and ginger roots reduced disease progression by 36.43–58.14%, mainly through hyphal competition. Similarly, *D. phaseolorum* BUP3/1, an endophytic fungus isolated from Jerusalem artichoke, inhibited *S. rolfsii* mycelial growth by 76.00% in a dual-culture assay, while *Trichoderma erinaceum* ST-KKU2 showed 63.63% inhibition under the same conditions. Moreover, *T. erinaceum* ST-KKU2 and *Daldinia eschscholtzii* 2NTYL11 reduced Sclerotium rot severity by 58.14% in a greenhouse pot experiment [14]. The 54.31% disease reduction achieved by *D. formosum* KKU-3.2 in the present study therefore falls within the range reported for fungal biocontrol agents evaluated under *in planta* conditions. Although direct comparisons should be interpreted cautiously because of differences in experimental systems and disease assessment methods, the observed suppression suggests that *D. formosum* may employ multiple antagonistic mechanisms, including competition for nutrients and space, secretion of antifungal metabolites, and production of extracellular enzymes. Collectively, these findings support the potential of *D. formosum* KKU-3.2 as a promising endophytic biocontrol agent for the management of Sclerotium rot in sunchoke. Importantly, pathogenicity and co-inoculation assays confirmed the biocontrol potential of *D. formosum* KKU-3.2 in sunchoke tubers. Tubers inoculated solely with *D. formosum* KKU-3.2 exhibited minimal disease symptoms compared with those inoculated with *S. rolfsii*. Although the observed lesion was substantially smaller, the occurrence of necrosis indicates that *D. formosum* KKU-3.2 may have weak pathogenicity toward Jerusalem artichoke under the conditions tested. This finding should be considered when evaluating the safety of this fungus as a potential biological control agent. The genus *Diaporthe* includes endophytic, saprobic, and pathogenic species, and some species can cause economically important diseases in plants [54,55]. For example, *D. helianthi* is a recognized pathogen of sunflower, and disruption of the *DhPKS1* gene resulted in reduced virulence, indicating a role for this gene in fungal pathogenicity [55]. Therefore, the pathogenicity observed in the present study represents an important consideration for the further evaluation of *D. formosum* as a biological control candidate. In contrast, co-inoculation with *S. rolfsii* significantly reduced lesion development, resulting in a 54.31% reduction in disease progression compared with the positive control. This reduction indicates that *D. formosum* KKU-3.2 may suppress disease development caused by *S. rolfsii*, although the underlying mechanisms remain to be experimentally validated. These findings suggest that *D. formosum* KKU-3.2 has potential as a candidate for further evaluation as a biological control agent against *S. rolfsii*. Early inoculation of *D. formosum* KKU-3.2 during the seedling production stage may facilitate endophytic colonization before pathogen infection, thereby reducing disease development and limiting the progression of Sclerotium rot in sunchoke tubers. In addition, endophytic fungi are known to enhance host defense responses and stress tolerance, which may further contribute to disease suppression [12,56]. Therefore, the present findings suggest that *D. formosum* KKU-3.2 has potential antagonistic activity against *S. rolfsii*, which may involve several complementary processes, including nutrient competition, extracellular enzymatic activity, and the production of potentially antifungal metabolites and secreted proteins. However, several limitations of the present study should be acknowledged. The proteins and metabolites identified through proteomic and metabolomic analyses were not individually validated functionally; therefore, their specific contributions to pathogen suppression remain to be confirmed. In addition, these analyses were performed using *D. formosum* KKU-3.2 grown in pure culture rather than in direct interaction with *S. rolfsii*. Consequently, the identified proteins and metabolites may represent constitutively produced or culture-dependent molecules and may not fully reflect those induced during pathogen interaction. Therefore, their potential contributions to fungal antagonism should be considered putative mechanisms supported by predicted functions or previously reported biological activities rather than experimentally demonstrated mechanisms. Future studies should focus on functionally validating candidate proteins and metabolites using purified compounds, targeted antifungal assays, and gene expression or gene-function approaches. Co-culture experiments between *D. formosum* KKU-3.2 and *S. rolfsii*, coupled with comparative proteomic and metabolomic analyses, would further help identify interaction-induced molecules and elucidate the mechanisms underlying antagonism. In addition, greenhouse and field trials are necessary to evaluate the efficacy, formulation, shelf stability, application methods, and practical applicability of *D. formosum* KKU-3.2 under agricultural conditions. Such studies will be important for determining whether this novel endophytic fungus can be developed as a reliable and sustainable biocontrol agent for the management of *S. rolfsii* in sunchoke.

## 5. Conclusions

In conclusion, the novel endophytic fungus *D. formosum* KKU-3.2 exhibited significant antifungal activity against *S*. *rolfsii* and effectively reduced disease progression in sunchoke tubers. Proteomic and metabolomic profiling revealed diverse bioactive proteins and secondary metabolites that may contribute to its antagonistic activity. Importantly, this study represents the first report describing the proteomic and metabolomic profiling of the novel species *D. formosum* sp. nov. These findings provide important insights into its biological characteristics and highlight the potential of *D. formosum* KKU-3.2 as a promising biocontrol agent for sustainable agricultural applications. Another aspect that deserves to be better investigated before proposing *D. formosum* as a candidate biocontrol agent is its pathogenicity on Jerusalem artichoke.

## Figures and Tables

**Table 1:** Details of the sequences used in the molecular phylogenetic analyses in this study.

*Diaporthe* Species	Strain/Isolate	Country	GenBank Accession Number		
			ITS	*Tef-1α*	*TUB2*
*D. acericola*	MFLUCC 17-0956 *	Italy	KY964224	KY964180	KY964074
*D. acuta*	PSCG 047 *	China	MK626957	MK654802	MK691225
*D. acuta*	PSCG 046	China	MK626958	MK654803	MK691224
*D. alangii*	CFCC 52556 *	China	MH121491	MH121533	MH121573
*D. alangii*	CFCC 52557	China	MH121492	MH121534	MH121574
*D. albosinensis*	CFCC 53066 *	China	MK432659	MK578133	MK578059
*D. alnea*	CBS 146.46 *	Netherlands	KC343008	KC343734	KC343976
*D. amygdali*	CBS 126679 *	Portugal	KC343022	KC343748	KC343990
*D. annellsiae*	BRIP 59731a *	Australia	OM918687	OM960596	OM960614
*D. apiculata*	CGMCC 3.17533 *	China	KP267896	KP267970	KP293476
*D. apiculata*	LC3364	China	KP267887	KP267961	KP293467
*D. arecae*	CBS 161.64 *	India	KC343032	KC343758	KC344000
*D. arengae*	CBS 114979 *	China	KC343034	KC343760	KC344002
*D. aseana*	MFLUCC 12-0299a *	Thailand	KT459414	KT459448	KT459432
*D. australiana*	BRIP 66145	Australia	MN708222	MN696522	MN696530
*D. betulae*	CFCC 50469 *	China	KT732950	KT733016	KT733020
*D. betae*	HMPHU 3001	China	MW882216	MW882222	MW882228
*D. betae*	HUMCC 3268	China	MW882217	MW882223	MW882229
*D. betae*	HUMCC 3269	China	MW882218	MW882224	MW882230
*D. biconispora*	CGMCC 3.17252 *	China	KJ490597	KJ490476	KJ490418
*D. biconispora*	YJ1	China	MN901242	MT113118	MT113116
*D. biguttulata*	CGMCC 3.17248 *	China	KJ490582	KJ490461	KJ490403
*D. biguttulata*	ZJUD48	China	KJ490583	KJ490462	KJ490404
*D. bohemiae*	CBS 143347 *	Czech Republic	MG281015	MG281536	MG281188
*D. bohemiae*	CBS 143348	Czech Republic	MG281016	MG281537	MG281189
*D. camptothecicola*	CFCC 51632	China	KY203726	KY228887	KY228893
*D. caryae*	CFCC 52563 *	China	MH121498	MH121540	MH121580
*D. caryae*	PSCG 520	China	MK626952	MK654895	MK691315
*D. betulicola*	CFCC 51128 *	China	KX024653	KX024655	KX024657
*D. betulina*	CFCC 52560 *	China	MH121495	MH121537	MH121577
*D. bohemiae*	CBS 143347 *	Czech Republic	MG281015	MG281536	MG281188
*D. bohemiae*	CBS 143348	Czech Republic	MG281016	MG281537	MG281189
*D. caryae*	CFCC 52563 *	China	MH121498	MH121540	MH121580
*D. caryae*	PSCG 520	China	MK626952	MK654895	MK691315
*D. collariana*	MFLUCC 17-2636 *	Thailand	MG806115	MG783040	MG783041
*D. discoidispora*	CGMCC 3.17255 *	China	KJ490624	KJ490503	KJ490445
*D. endophytica*	CBS 133811 *	Brazil	KC343065	KC343791	KC344033
*D. eres*	AR5193 *	Germany	KJ210529	KJ210550	KJ420799
***D. formosum***	**KKU-3.2**	**Thailand**	**PZ203759**	**PZ399302**	**PZ435428**
*D. ganjae*	CBS 180.91 *	USA	KC343112	KC343838	KC344080
*D. ganjae*	PSCG489	China	MK626955	MK654897	MK691287
*D. helicis*	CBS 138596 *	France	KJ210538	KJ210559	KJ420828
*D. hubeiensis*	JZB320123 *	China	MK335809	MK523570	MK500148
*D. longicicola*	CGMCC 3.17089 *	China	KF576267	KF576242	KF576291
*D. longicolla*	FAU599 *	USA	KJ590728	KJ590767	KJ610883
*D. middletonii*	BRIP 54884e *	Australia	KJ197286	KJ197248	KJ197266
*D. miriciae*	BRIP 54736j *	Australia	KJ197282	KJ197244	KJ197262
*D. oncostoma*	CBS 589.78	France	KC343162	KC343888	KC344130
*D. oraccinii*	CGMCC 3.17531 *	China	KP267863	KP267937	KP293443
*D. penetriteum*	CGMCC 3.17532 *	China	KP714505	KP714517	KP714529
*D. pseudomangiferae*	CBS 101339 *	Dominican Republic	KC343181	KC343907	KC344149
*D. pseudophoenicicola*	CBS 462.69 *	Spain	KC343184	KC343910	KC344152
*D. sambucusii*	CFCC 51986 *	China	KY852495	KY852507	KY852511
*D. sambucusii*	CFCC 51987	China	KY852496	KY852508	KY852512
*D. tectonendophytica*	MFLUCC 13-0471 *	Thailand	KU712439	KU749367	KU743986
*D. tectonigena*	MFLUCC 12-0767 *	Thailand	KU712429	KU749371	KU743976
*D. tuyouyouiae*	BRIP 75017a *	Australia	OQ917074	OQ889558	OQ889559
*D. unshiuensis*	CGMCC 3.17569 *	China	KJ490587	KJ490466	KJ490408
*D. unshiuensis*	CGMCC 3.17568	China	KJ490586	KJ490465	KJ490407
*D. xishuangbanica*	CGMCC 3.18282 *	China	KX986783	KX999175	KX999216
*D. zaofenghuang*	CGMCC 3.20271 *	China	MW477883	MW480871	MW480875
*D. zaofenghuang*	TZFH3	China	MW477884	MW480872	MW480876

* Strain KKU-3.2 is highlighted in red to indicate the novel isolate characterized in the present study.

**Table 2:** Morphological comparison of the endophytic fungus *D. formosum* sp. nov with other *Diaporthe* species.

Taxon	Conidiogenous Layer	Alpha Conidia	Beta Conidia	Reference
*D. aseana* MFLUCC 12-0299a	*Conidiogenous cells* 6–10 × 2–3 µm	6–9 × 2–3 µm	Not observed	[27]
*D. pseudophoenicicola* CBS 462.69t	*Conidiogenous cells* (10.5–)12.5–18(–22) × 1.3–1.5 μm (n = 30)	5–6.5(–7) × 2.3–3.0 μm (n = 30)	Not observed	[28]
*D. australiana* BRIP 66145t	*Conidiogenous cells* 10–20 × 1–2.5 μm	5–8.5 × 1.5–2 μm	Not observed	[29]
*D. formosum* KKU-3.2	Not observed	3.5–5.78 × 1.64–2.66 (mean 4.77 × 2.07, n = 30)	Not found	This study

**Table 3:** The similarity of the ITS, β-Tubulin, and *Tef-1α* sequences between the fungal strains obtained in this study and the top five most closely related *Diaporthe* species retrieved from the GenBank database.

Query Species	Related Species	Sequence Similarity Value (%)		
		ITS	*Tef-1α*	*TUB2*
*Diaporthe formosum* KKU-3.2	*D. tuyouyouiae* BRIP 75017a	98.55	95.12	92.94
	*D. annellsiae* BRIP 59731a	98.37	96.47	96.61
	*D. aseana* MFLUCC 12-0299a	98.18	95.20	89.80
	*D. pseudophoenicicola* CBS 462.69t	97.25	96.49	95.18
	*D. australiana* BRIP 66145t	96.39	96.28	95.54

**Table 4:** Metabolic profiling and predicted antifungal activity/mechanisms of tentatively identified bioactive metabolites from *D. formosum* KKU-3.2 against pathogenic fungi.

Chemical Class	Metabolite Name	Antifungal Mechanism	Reference
Coumarins	Byakangelicol	Membrane disruption	[30]
	Angelol D	Oxidative stress induction and inhibition of hyphal growth	[31]
	7,8-Dihydroxy-4-methylcoumarin	Membrane destabilization and ROS accumulation	[32]
	7-Hydroxy-4-methylcoumarin	Inhibition of fungal enzymes and mitochondrial function	[31]
	4-Methylumbelliferone	Cell membrane perturbation and inhibition of fungal metabolism	[33]
	4-Methylesculetin	ROS-mediated cellular damage	
Phenolic and Organic Acids	Caffeic acid,	Membrane damage, ROS generation, and inhibition of spore germination	[34]
	Ethyl caffeate	Membrane permeabilization and inhibition of fungal growth	[35]
	Trans-Ferulic acid	Ergosterol interference and oxidative stress	
	Propionic acid	Cytoplasmic acidification and metabolic inhibition, accumulation of ROS	[36,37]
	Nicotinic acid	Disruption of NAD-related metabolic pathways	[38]
Limonoids (Triterpenoids)	Limonin	Membrane disruption and inhibition of fungal proliferation	[39]
	Obacunone	Inhibition of fungal metabolism and hyphal growth	[40]
Phenolics and Depsides	Lecanoric acid	Membrane destabilization and oxidative stress	[41,42]
	Divaric acid	Cell membrane damage	
	Catechol	ROS generation and lipid peroxidation	[43,44]
	3-Methylcatechol	Membrane oxidation and cellular leakage	[43]
Pyrones and Pyridines	Kojic Acid	Metal chelation and inhibition of melanin biosynthesis	[45]
	Maltol	Oxidative stress induction and metal chelation, cell wall damage	[46]
	3-Hydroxypyridine	Enzyme inhibition and oxidative stress; inhibition of fungal metabolism	
Flavonoids	Heptamethoxyflavone	Membrane perturbation and inhibition of fungal enzymes	[47]
	Hesperetin	Membrane disruption and mitochondrial dysfunction	[48,49]
Sesquiterpene Lactones	(+)-Costunolide	Membrane disruption, ROS generation, and thiol-reactive α-methylene-γ-lactone activity	[50]
Indoles	Indole-3-acetaldehyde	Interference with fungal conidial death and metabolism	[51]

## Data Availability

The datasets obtained and analyzed in the current study are available from the corresponding author upon reasonable request.

## Supplementary Materials

The following supporting information can be downloaded at https://www.mdpi.com/article/10.3390/jof12100794/s1, Table S1: Host plants, numbers of samples examined, and numbers of endophytic fungal isolates recovered. Table S2: Effect of fungal endophyte on the pathogenic fungus *S. rolfsii* using the dual culture technique. Table S3: Proteins identified from the endophytic fungus *D. formosum* sp. nov. using LC-MS-based analysis. Table S4: Metabolomics profiling of the endophytic fungus *D. formosum* sp. nov. using LC-MS-based analysis. Data File S1: The sequence alignment used for phylogenetic tree construction.
