# Supplementary material for: A New Species of Diaporthe: Metabolite Profile and Ability to Suppress Sclerotium Rot of Sunchoke
Source: J Fungi (Basel). 2026 Oct 21;12(10):794. doi: 10.3390/jof12100794 (PMC13706414; doi:10.3390/jof12100794)
Supplement: Supplementary file 1 [file jof-12-00794-s001.zip › Supplementary Data File S1.pdf]

Supplementary Data File S1:

The sequence alignment used for phylogenetic tree construction.

>*Diaporth formosum* KKU-3.2

C C C T T T G T G A A C T T A T A C C T T A - C T G T T G C C T C G G C G - C A T G C T G G C C C C C -

ATGGGGTCCCTCGAGACGAGGAGCAGGCACGCCGGCGGCCAAGTTAACTCTTGTTTTTACACTGAAACTCT

GAGAAAAAACACAAATGAATCAAAACTTTCAACAACGGATCTCTTGGTTCTGGCATCGATGAAGAACGCA

GCGAAATGCGATAAGTAATGTGAATTGCAGAATTCAGTGAATCATCGAATCTTTGAACGCACATTGCGCCC

TCTGGTATTCCGGAGGGCATGCCTGTTCGAGCGTCATTTCAACCCTCAAGCATTGCTTGGTGTTGGGGCACT

GCCTGTAAAAGGGCAGGCCCTGAAATCTAGTGGCGAGCTCGCCAGGACCCCGAGCGTAGTAGT---

T A A A C C C T C G C - T T T G G A A G G C C C T G G C G G T G C C C T - G C C G T T A A A - C C C C C A A -

CTTTTGAAAATTTGACCTCGGATCAGGTAGGAATACCCGCTGAACTTAAGCATAGGTGGAGGGGCTAAGCA

TTTGTCGTAATCGGAGA------------------------TGGGCGAAGGCAACAGGGTGTAGCCGAC-------

CTTCTTGATGAAGGAGGAGGTCTCCTGATCTCCTGGTATGGAGCTGCTACCCAGGGTGTAGGCGAGCAGAG

C G C T C A C G A G T C T G G C C A T C T C C A G C C T -

CGAACTCATATGATGAGAATGGCCAGTCGGCCTGGGAGGTACCAGTGCATTCTTGATGAAGTCACGGTGAC

C G G G A G C G - - - - - - - T C A A G C A G G C A G C G - - - - - - - -

AGTGCTGACAAGTTGGATCCACAGCAACTCGATCCTTTGTTGGAATGCGTAAGTTGCT-

C C T G T C A A C A C C G C -

CGGACCTTATCATCGCCACCTGTAGCTGACACGTTTCCCAGGGTAACCAAATCGGTGCTGCTTTCTGGTGC--

G T C C - - - - - - - - - - - - - - - - - - A C C G C C G C G A G C T C -

G A C G C G C G A C A A T A C G A C C T C G A A C C A T C G T T A C T G A C C T C G A C T C -

TTAGGCAAACCATCTCTGGCGAGCACGGTCTCGACAGCAATGGCGTGTATGCACCTCCTATTCCCTACCTCC

AGATCTCGTCCTCCCTGCCGGCTTGGCACTGACAATCATACAGTTACAACGGCACTTCCGAGCTCCAGCTCG

A G C G C A T G A A C G T C T A C T T C A A C G A G G T C A G T A A A C A G C C A C G T C G - - - - -

TTGACCATCTGCAGCACGATTTTCTGCCGTCGCCGGGACCTTGCTAACGCGTTATCGCCCAGGCTTCCGGCA

ACAAGTATGTCCCTCGCGCCGTCCTCGTCGATCTCGAGCCCGGTACCATGGACGCCGT

>KY964224.1 Diaporthe acericola strain MFLUCC 17-0956

C C C T T T G T G - A C T T A T A C C - C A - C T G T T G C C T C G G C G -

CAGGCCGGCCTCTGCTGAGGCCCCCTGAAACAGGGAGCA-GCCCGCCGGTGGCCAACTAAACTC-

TGTTTCTATAGTGAATCTCTGAGAAAAAACATAAATGAATCAAAACTTTCAACAACGGATCTCTTGGTTCTG

GCATCGATGAAGAACGCAGCGAAATGCGATAAGTAATGTGAATTGCAGAATTCAGTGAATCATCGAATCTT

TGAACGCACATTGCGCCCTCTGGTATTCCGGAGGGCATGCCTGTTCGAGCGTCATTTCAACCCTCAAGCCTG

GCTTGGTGATGGGGCACTGCCTGTAAAAGGGCAGGCCCTGAAATCTAGTGGCGAGCTCGCCAGGACCCCG

AGCGTAGTAGT---TATA-TCTCGC-TTTGGAAGGCCCTGGCGGTGCCCT-GCCGTTAAA-CCCCCAA-
CTTCTGAAAATT---GGATGACT--------CCAC-----------------
CAAGCCCACCGTGCGCACCGTCATCATTGGTGCGCGACTGCCTGCGCGGCTTCATCACCCCTCAAC---
CATTTTCACCCCTCCCTCTGGGTTTTTATTTTCAGTGCGGGTGCGGGGCGCGCTTATCAG-
CCGCTTATCTCCTATG--
CAAAACCCTGTGGCATTAACCACTCCTTGCCACCGCCAACACCAACTCTACCACTTTCAA-
CCCTATCAATCTC------------
CCAGGCGAAACGTCAAGAATATGAAGCTGACTTACTTTCCACACAGCCGCCGAGCTGGGTAAGGGTC-------
---ATCGCGGT-
CTCGACGCGCGATGACAGGACCTCGCAACATCGTTATTGACTTCGACTT-
TTAGGCAAACCATCTCTGGCGAGCACGGCCTCGACAGCAATGGCGTGTATGTACCTCCCATTCCCTACTCGT
CGGTCTCGTCCGCCGGC-----
TTGGCACTGACAGCTTCACAGTTACAACGGCTCTTCTGAGCTCCAGCTCGAGCGCATGAACGTCTACTTCAA
CGAGGTCAGTCCTCGATATTTTTATTGCACCCAC-ATCTCCAA---
ATTGCTGTGTTGTCGTTTGGACTTTGCTGACACCTTATCGTCCAGGCTTCCGGCAACAAGTATGTGCCTCGC
GCTGTCCTCGTCGATCTCGAGCCCGGTACCATGGACGCCGT

>MK626957.1 Diaporthe acuta strain PSCG 047

CCCTATGTGAAC-TATACC--T-CTGTTGCCTCGGCG-C-TGCTGGTCTTTAT--AGGCCCTTTT-
GGTAAAGAGAAGGCACGCCGGCGGCCAAAACAACTC-TGTTTTTATACTGAAACTCTGAG-CAATACA-
AAATGAATCAAAACTTTCAACAACGGATCTCTTGGTTCTGGCATCGATGAAGAACGCAGCGAAATGCGATA
AGTAATGTGAATTGCAGAATTCAGTGAATCATCGAATCTTTGAACGCACATTGCGCCCTCTGGTATTCCGGA
GGGCATGCCTGTTCGAGCGTCATTTCAACCCTCAAGCATTGCTTGGTGTTGGGGCACTGCTTTTAACGAAGC
AGGCCCTGAAATCTAGTGGCGAGCGCGCCAGGACCCCGAGCGTAGTAGT---TAAACCCTCGC-
TTTGGAAGGCCCTGGCGGTGCCCT-GCCGTTAAA-CCCCCAA-
CTTTTGAAAATTTGACCTCGGATCAGGTAGGAATACCCGCTGAACTTAAGCATA----------------------------------
----
TCCTCTGCCCTCACCTTTTGCCTCAAATCATCAGTGTGCGGCCGTTGGTACGGCTTCGTCATACTGAGGGGC
GCATTTTCACCCCTCGCTCTGGATTTTCCATTTTCAGTGCGGGTGCGGGGTGCGCTTATCAGGGAGCTTATC
TCCTACACCCAAAACCCTGTTGCAACAACAAC------------
CTACCGCTACTTCATTCATCCTCAACCCCCATCGACATCCTTGAAT-CAA-TCAAGCAAACCGCCAGG-
AAATCATGCTGACTTTCTATCTACATAGCCGCTGAGCTTGGTAAGGGTT---
---GTTGGGCTCCACCGCGAGCT-
CGACGCGCGACAACATGACCTCGAGCCATCGTTACTGACCTCGACTC-
TCAGGCAAACCATCTCTGGCGAGCACGGCCTCGACAGCAATGGCGTGTATGCACCTCCTATTTCCTGCCTTC

AGATCTCGTCCTCCCTGCCGGCTTGGCACTGACAATCACACAGTTACAACGGCACTTCCGAGCTCCAGCTCG
AGCGCATGAACGTCTACTTCAACGAGGTAAGTCAACAGCCACGTCG-----
TTGATCATCTGCAGCACGATTTTCTGCCGTCGCCGGGGCCTTGCTAACGCGTTATCGCCCAGGCCTCCGGCA
ACAAGTATGTCCCTCGCGCCGTCCTCGTCGATCTCGAGCCCGGTACCATGGACGCCGT

>MK626958.1 Diaporthe acuta strain PSCG 046

CCCTATGTGAAC-TATACC--T-CTGTTGCCTCGGCG-C-TGCTGGTCTTTAT--AGGCCCTTTT-
GGTAAAGAGAAGGCACGCCGGCGGCCAAAACAACTC-TGTTTTTATACTGAAACTCTGAG-CAATACA-
AAATGAATCAAAACTTTCAACAACGGATCTCTTGGTTCTGGCATCGATGAAGAACGCAGCGAAATGCGATA
AGTAATGTGAATTGCAGAATTCAGTGAATCATCGAATCTTTGAACGCACATTGCGCCCTCTGGTATTCCGGA
GGGCATGCCTGTTCGAGCGTCATTTCAACCCTCAAGCATTGCTTGGTGTTGGGGCACTGCTTTTAACGAAGC
AGGCCCTGAAATCTAGTGGCGAGCGCGCCAGGACCCCGAGCGTAGTAGT---TAAACCCTCGC-
TTTGGAAGGCCCTGGCGGTGCCCT-GCCGTTAAA-CCCCCAA-
CTTTTGAAAATTTGACCTCGGATCAGGTAGGAATACCCGCTGAACTTAAGCATA-----------------------------------
----
TCCTCTGCCCTCACCTTTTGCCTCAAATCATCAGTGTGCGGCCGTTGGTACGGCTTCGTCATACTGAGGGGC
GCATTTTCACCCCTCGCTCTGGATTTTCCATTTTCAGTGCGGGTGCGGGGTGCGCTTATCAGGGAGCTTATC
TCCTACACCCAAAACCCTGTTGCAACAACAAC------------
CTACCGCTACTTCATTCATCCTCAACCCCCATCGACATCCTTGAAT-CAA-TCAAGCAAACCGCCAGG-
AAATCATGCTGACTTTCTATCTACATAGCCGCTGAGCTTGGTAAGGGTT--
---TGTTGG--CTAACCGCGAGCT-CGACGCGCGACA-
CATGACCTCGAGCCATCGTTACTGACCTCGACTC-
TCAGGCAAACCATCTCTGGCGAGCACGGCCTCGACAGCAATGGCGTGTATGCACCTCCTATTTCCTGCCTTC
AGATCTCGTCCTCCCTGCCGGCTTGGCACTGACAATCACACAGTTACAACGGCACTTCCGAGCTCCAGCTCG
AGCGCATGAACGTCTACTTCAACGAGGTAAGTCAACAGCCACGTCG-----
TTGATCATCTGCAGCACGATTTTCTGCCGTCGCCGGGGCCTTGCTAACGCGTTATCGCCCAGGCCTCCGGCA
ACAAGTATGTCCCTCGCGCCGTCCTCGTCGATCTCGAGCCCGGTACCATGGACGCCGT

>MH121491.1 Diaporthe alangii strain CFCC 52556

CCCTTTGTGAACTTATACCATA-CTGTTGCCTCGGCG-
CTGGCCGGCCTCCACCGAGGCCCCCTGAGACAGGGAGCA-
GCCCGCCGGCGGCCAAACAAACTCTTGTTTCT-
TAGTGAATCTCTGAGAAAAAACATAAATGAATCAAAACTTTCAACAACGGATCTCTTGGTTCTGGCATCGA
TGAAGAACGCAGCGAAATGCGATAAGTAATGTGAATTGCAGAATTCAGTGAATCATCGAATCTTTGAACGC

ACATTGCGCCCTCTGGTATTCCGGAGGGCATGCCTGTTCGAGCGTCATTTCAACCCTCAAGCCTGGCTTGGT
GTTGGGGCACCGCCTTTAAAAGGGCGGGCCCTGAAATCTAGTGGCGAGCTCGCCAGGACCCCGAGCGTAGT
AGT---TATA-TCTCGT-TCTGGAAGGCCCTGGCGGTGCCCT-GCCGTTAAA-CCCCCAA-CTTCTGAAA-----------
- -
GAGAAGGAAGGTGAGTAAACATCAAAGTTACGGCATACACTCCCTTGTACCCAGTTGCACATCAGGATTAC
T G G C G C G C T G C C G T T T G C G C G G C T T C G T C A C A C C C G C C A G -
GCATTTTCACCCCTCCCTCTGGATTTTCCATTTTCAGTGCGGGTGCGGGGTGCGCTTATCAGGCGGCTTATCT
CTGACGCTACAAACCCTGCTGCATCAACCATTCCTTGTCGCTGCCACCACCA---CCATCACCGTCAA-
A T T C A T C G C - - - - - - - - - - - - - -
ACGGTCAAATTGATCACGTTGGAGATGAAGCTGACTTTCCATCTCTATAGCCGCCGAGCTCGGTAAGGGTT
T G C G T A A G T T G C T T C C T G T C A A C A C C G C - C C G A C C T T A T C G C C A C C T - - -
C T A G C T G A C A C G T T T C C C A G G G T A A C C A A A T C G G T G C T G C T T T C T G G T G C G T C C C A G C T C -
CAGCTCAGAGTCGACCACCGCGACAATCGACGAGCGACAACAGTAGCTCGTAGCATTGATACTGACATCG
G C T C C -
TAGGCAAACCATCTCTGGCGAGCACGGCCTCGACAGCAATGGCGTGTATGCACCTCCTATTCCCTGCCTACT
GGTCTCGTCCTCTCTCCTACCTTGGCACTGACAATGGCACAGCTACAACGGCACTTCTGAGCTCCAGCTCGA
GCGCATGAACGTCTACTTCAACGAGGTAAGCCAAAGCCCACGTTGTCAATCCGGACACCTGCAGCACAATC
CCTTGCCACCGCCAAGGCCTAGCTAACGCGTTATCGTCCAGGCCTCCGGCAACAAGTATGTGCCTCGCGCC
GTCCTCGTCGATCTCGAGCCCGGTACCATGGATGCCG-

>MH121492.1 Diaporthe alangii strain CFCC 52557

C C C T T T G T G A A C T T A T A C C A T A - C T G T T G C C T C G G C G -
C T G G C C G G C C T C C A C C G A G G C C C C C T G A G A C A G G G A G C A -
G C C C G C C G G C G G C C A A A C A A A C T C T T G T T T C T -
TAGTGAATCTCTGAGAAAAAACATAAATGAATCAAAACTTTCAACAACGGATCTCTTGGTTCTGGCATCGA
TGAAGAACGCAGCGAAATGCGATAAGTAATGTGAATTGCAGAATTCAGTGAATCATCGAATCTTTGAACGC
ACATTGCGCCCTCTGGTATTCCGGAGGGCATGCCTGTTCGAGCGTCATTTCAACCCTCAAGCCTGGCTTGGT
GTTGGGGCACCGCCTTTAAAAGGGCGGGCCCTGAAATCTAGTGGCGAGCTCGCCAGGACCCCGAGCGTAGT
AGT---TATA-TCTCGT-TCTGGAAGGCCCTGGCGGTGCCCT-GCCGTTAAA-CCCCCAA-CTTCTGAAA-----------
- -
GAGAAGGAAGGTGAGTAAACATCAAAGTTACGGCATACACTCCCTTGTACCCAGTTGCACATCAGGATTAC
T G G C G C G C T G C C G T T T G C G C G G C T T C G T C A C A C C C G C C A G -
GCATTTTCACCCCTCCCTCTGGATTTTCCATTTTCAGTGCGGGTGCGGGGTGCGCTTATCAGGCGGCTTATCT
CTGACGCTACAAACCCTGCTGCATCAACCATTCCTTGTCGCTGCCACCACCA---CCATCACCGTCAA-
A T T C A T C G - - - - - - - - - - - - - - C -

ACGGTCAAATTGATCACGTTGGAGATGAAGCTGACTTTCCATCTCTATAGCCGCCGAGCTCGGTAAGGGTT

TGCGTAAGTTGCTTCCTGTCAACACCGC-CCGACCTTATCGCCACCT---

CTAGCTGACACGTTTCCCAGGGTAACCAAATCGGTGCTGCTTTCTGGTGCGTCCCAGCTC-

CAGCTCAGAGTCGACCACCGCGACAATCGACGAGCGACAACAGTAGCTCGTAGCATTGATACTGACATCG

G C T C C -

TAGGCAAACCATCTCTGGCGAGCACGGCCTCGACAGCAATGGCGTGTATGCACCTCCTATTCCCTGCCTACT

GGTCTCGTCCTCTCTCCTACCTTGGCACTGACAATGGCACAGCTACAACGGCACTTCTGAGCTCCAGCTCGA

GCGCATGAACGTCTACTTCAACGAGGTAAGCCAAAGCCCACGTTGTCAATCCGGACACCTGCAGCACAATC

CCTTGCCACCGCCAAGGCCTAGCTAACGCGTTATCGTCCAGGCCTCCGGCAACAAGTATGTGCCTCGCGCC

GTCCTCGTCGATCTCGAGCCCGGTACCATGGATGCCG-

>MK432659.1 Diaporthe albosinensis isolate CFCC 53066

CCCTTTGTGAACTTATACCTTA-CTGTTGCCTCGGCG-

AAGGCTGGCCCCCACGGGGGCCCCTTCAGGTGTTGAGACAGCCCGCCGGCGGCCAAGTTAACTCTTGTTTT

TACACTGAAACTCTGAGATAAAACATAAATGAATCAAAACTTTCAACAACGGATCTCTTGGTTCTGGCATC

GATGAAGAACGCAGCGAAATGCGATAAGTAATGTGAATTGCAGAATTCAGTGAATCATCGAATCTTTGAAC

GCACATTGCGCCCTCTGGTATTCCGGAGGGCATGCCTGTTCGAGCGTCATTTCAACCCTCAAGCCTGGCTTG

GTGATGGGGCACTGCTTTTCAAGAAGCAGGCCCTGAAATTCAGTGGCGAGCTCGCCAGGACCCCGAGCGCA

GTAGT---TAAACCCTCGC-TCTGGAAGGCCCTGGCGGTG--

- - - - - - - - - - - - - - - -

AAGGTTAGTAAACACCACAATCACGGAACATGCTACCCTGCCCTCCTACTGCACCTCAAACCATGAGCCCG

CAGCTGCTCGTGTGGCTTCGTCATAACGGGGGGCGCATTTTCACCCCTCGCTTTGGATTTTCCATTTTCAGTG

CGGGTGCGGGGTGCGCTTATCAGGGGGCTTATCTCCTACTCCCAAAACCCTGTTGCATCACTCACC-

CCTGCCACCACCACTACCAAACCGCCCACCATCAA-TCCCACCACCCTCCTTCAAT-ACA-

ACTCGTGAAGCGCGTCGAACATTATGCTGACCCTCTATCTACACAGCCGCCGAGCTTGGTAA-----

TGCGTAAGTTGCT-CCTGTCAACACCAG-

TGGACCTTATCATCGCCACCTGTAGCTGACACGTTTCCCAGGGTAACCAAATCGGTGCTGCTTTCTGGTGCG

T C C C A G C T -

CCAGCTCCAAGTCCACCACCGCGACGCTCGACGCGCGATAATACGACCTCGAGGCATCGTTACTGACCTCG

A C T C -

TTAGGCAAACCATCTCTGGCGAGCACGGCCTCGACAGCAATGGCGTGTATGCACCTCCTATTCCCTGCCCA

C C G A T C T T G G C C -

CTCTTTCGCCTTGGCACTGACAATTGCACAGTTACAACGGCACCTCTGAGCTCCAGCTCGAGCGCATGAAC

GTCTACTTCAACGAGGTAAGTAAAAAACCACGGCATCCGTTTTGACATCTGCAAAATGGTTTCCTGCCGTC

GCTCGGACCTTGCTAACGCGTTATCGCCCAGGCCTCCGGCAACAAGTATGTTCCTCGCGCCGTCCTCGTCGA
TCTCGAGCCCGGTACCATGGACGCCGT

>KC343022.1 Diaporthe amygdali strain CBS 126679

CCCTTTGTGAACTTATACCTTA-CTGTTGCCTCGGCG-CAGGCCGGCTCCCCTGGGGGCCCCTC--
GACGAGGAGCAGGCTCGCCGGCGGCCAAGTTAACTCTTGTTTTTAATTTGAAACTCTGAGATAAAACATAA
ATGAATCAAAACTTTCAACAACGGATCTCTTGGTTCTGGCATCGATGAAGAACGCAGCGAAATGCGATAAG
TAATGTGAATTGCAGAATTCAGTGAATCATCGAATCTTTGAACGCACATTGCGCCCTCTGGTATTCCGGAGG
GCATGCCTGTTCGAGCGTCATTTCAACCCTCAAGCCTGGCTTGGTGATGGGGCACTGCCTTTAAAAGGGCA
GGCCCTGAAATTCAGTGGCGAGCTCGCCAGGACTCCGAGCGCAGTAGT---TAAACCCTCGC-TTTGGAAGG-
ACTGGCGGTGCCCT-GCCGTTAAA-CCCCCAA-
CTCTTGAAAATTTGACCTCGGATCAGGTAGGAATACCCGCTGAACTTAAGCATAGAGAAGGAAGGTTAGTA
AACATCACAATCGCAACACATTCTGCATGCCCCTTCCTCGACTTCATAAATCCTGGGCGTGCGCCCGTTC-
CACAGCGTCATCACACCG-
GGGGCGCATTTTCACCCCTCGCTCTGGATTTTCCATTTTCAGTGCGGGTGCGGGGTGCGCTTATCAGCGAGC
TTATCTCCCAC-CCGAAAACCCTGCTACATCACTACA----TACCACCACCACTACCA----------------CCTCATCT-
TCTACTACAAG-AGA-TCTTG-------------
GATCATGCTGACTTCCCATCTTCACAGCCGCTGAGCTCGGTAAGGGTTTGCGTAAGTTGCT--
CCTGTCAACACAC-
CACCGCACCTTATCGCCGCCTGTAGCTGACACGTTTCCCAGGGTAACCAAATCGGTGCTGCTTTCTGGTGCG
TCCCAGCTC--------------
ATCACCGCGATACTCGACGCGCGACAACACGACCTCGCAACATCCTTACTGACCTCGACTC-
GTAGGCAAACCATCTCTGGCGAGCACGGCCTCGACACCAATGGCGTGTATGCACCTCCTATTCCATGCCCA
TCAATCTCGGCCTCGGGGA----
TTGGCACTGACAATTGCACAGCTACAACGGCACTTCCGAGCTCCAGCTCGAGCGCATGAACGTCTACTTCA
ACGAGGTAAGTCAATCACCATGTCATGGCGTTAAC-
AGCCCCAACACGATGCTCTTTTGTCGCTTGGGCTTTGCTGACCGCTTATCGCCCAGGCCTCCGGCAACAAGT
ATGTTCCCCGCGCCGTCCTCGTCGATCTCGAGCCCGGTACCATGGACGCCGT

>KC343024.1 Diaporthe anacardii strain CBS 720.97

CCCTTTGTGAACTTATACCTTA-CTGTTGCCTCGGCG-CAGGCCGTCCCCC-
CTGGGGTCCCTTGGAACAAGGAGCA-GCCGGCCGGTGGCCAAATTAACTC-
TGTTTTTACACTGAAACTCTGAGATAAAACATAAATGAATCAAAACTTTCAACAACGGATCTCTTGGTTCTG
GCATCGATGAAGAACGCAGCGAAATGCGATAAGTAATGTGAATTGCAGAATTCAGTGAATCATCGAATCTT

TGAACGCACATTGCGCCCTCTGGTATTCCGGAGGGCATGCCTGTTCGAGCGTCATTTCAACCCTCAAGCCTG
GCTTGGTGTTGGGGCACTGCCTGTAAAAGGGCAGGCCCTGAAATATAGTGGCGAGCTCGCCAGGACTCCGA
GCGTAGTAGT---TAAACCCTCGC-TTTGGAAGG-CCTGGCGGTGCCCT-GCCGTTAAA--CCCCAA-
CTTTTGAAAATTTGACCTCGGATCAGGTAGGAATACCCGCTGAACTTAAGCATAGAGAAGGAAGGTCAGTA
AACACCGCAATCACAGAACAAGTTACCTTGCCCTCCGATTTTGCCCCAAATCATCAGCATGCGGCTGTTCGT
GCGGCTTCGTTGCACTAGGAGGCGCATTTTCACCCCTCCCTCTGGATTTTCCATTTTCAGTGCGGGTGCGGG
GTGCGCTTATCAGGGAGCTTATCTCCTACACTCAAAACCCTGTTACATCACTCACCCAT---------
G C T A C C A C T A C G T C C A C C A T C A A - T C T C A T C G C C G C T C T T C C A C - G C A -
ACGTTTAAACCGCATCAAAAACTATGCTGACCTTCCATCTACACAGCCGCCGAGCTTGGTAAGGGTTTGCG
T A A G T - - - - - - - - - - C A A C A C C A C -
CGGACCTTATCATCGCCACCTGTAGCTGACACGTTTCCCAGGGTAACCAAATCGGTGCTGCTTTCTGGTGC--
G T C C C A G C C C A G C T C C A A G T C C A C C G C C G C G A C C T C -
G A C G C G C G A C A A G T C G A C C C C G A A G C A T C G T T A C T G A C C T C G A C T T -
TCAGGCAAACCATCTCTGGCGAGCACGGTCTCGACAGCAATGGCGTGTATGCACCTCCTTATTCCTGTCTTC
A A A T C T C G T C C T T - -
TGCCGGCTTGGCACTGACAATCACACAGTTACAACGGCACTTCCGAGCTCCAGCTCGAGCGCATGAACGTC
T A C T T C A A C G A G G T A A G T C A T C A G C C A C G T T G - - - - -
TTGACCATCTAAAGCTCGAATTCCTGCCGTCGCCGGGGCTCTGCTAATGCGTTATCGCCCAGGCCTCCGGCA
ACAAGTATGTGCCCCGCGCCGTCCTCGTCGATCTCGAGCCCGGTACCATGGACGCCGT

>OM918687.1 Diaporthe annellsiae strain BRIP 59731a

C C C T T T G T G A A C T T A T A C C T T A - C T G T T G C C T C G G C G - A A T G C T G G C C C C C -
TCGGGGTCCCCTGAGACAGGGAGCAGGCACGCCGGCGGCCAAGTTAACTCTTGTTTTTACACTGAAACTCT
GAGAAAAAACACAAATGAATCAAAACTTTCAACAACGGATCTCTTGGTTCTGGCATCGATGAAGAACGCA
GCGAAATGCGATAAGTAATGTGAATTGCAGAATTCAGTGAATCATCGAATCTTTGAACGCACATTGCGCCC
TCTGGTATTCCGGAGGGCATGCCTGTTCGAGCGTCATTTCAACCCTCAAGCATTGCTTGGTGTTGGGGCACT
GCCTGTAAAAGGGCAGGCCCTGAAATCTAGTGGCGAGCTCGCCAGGACCCCGAGCGTAGTAGT---
TAAACCCTCGC-TTTGGAAGGCCCTGGCGGTGCCCT-GCCGTTAAA-CCCCC---------------------------------------
- - - - - - - - - - - - - -
GAGAAGGAAGGTTAGTAAACATCACAATCATAAAATGCTCTCCTCTGCCCTCACCCTTTGCCTCAAATCATC
AGTGTTCGGCTGTTGGTACGGCTTCGTCATACTGAGGGGCGCATTTTCACCCCTCGCTCTGGATTTTCCATTT
TCAGTGCGGGTGCGGGGTGCGCTTATCAGGGAGCTTATCTCCTACACCCAAAACCCTGTTGCTACAGCAAC
TCCTTGCTGTTACTACCACCACTTCATTCACACTCACCCCCCATCGACATGCTTGAAT--
CAATCAAGCAAACC---CAGGAAATCATGCTGACTTTCTATCTACACAGCCGCCGAGCTTGGTAA-----
T G C G T A A G T T G C T - C C T G T C A A C A C C G C -

CGGACCTTATCATCGCCACCCGTAGCTGACACGTTTCCCAGGGTAACCAAATCGGTGCTGCTTTCTGGTGC--

GTCC-----------------

ACCACCGCGAGCTCTGACGCGCGACAATACGACCTCGAGCCATCGTTACTGACCTCGACTC-

TCAGGCAAACCATCTCTGGCGAGCACGGCCTCGACAGCAATGGCGTGTATGCACCTCCTATTCTATACCTCC

AGATCTCGTCCTCCCTGCCGGCTTGGCACTGACAATCATACAGTTACAACGGCACTTCCGAGCTCCAGCTCG

AGCGCATGAACGTCTACTTCAACGAGGTAAGTCGACAGCCACGTCG-----

TTGACCATCTGCAGCACGATTTTCTGCCGTCGCTGAGGCCTTGCTAACGCGTTATCGCTCAGGCCTCCGGCA

ACAAGTATGTCCCTCGCGCCGTCCTCGTCGATCTCGAGCCCGGTACCATGGACGCCGT

>KP267896.1 Diaporthe apiculata strain LC3418

CCCTTTGTGAACTTATACCTTA-CCGTTGCCTCGGCG-CCAGCTGGTCCTC--CGGGGCCCCTC--

GGTGTTGAGAGAGCACGCCGGCGGCCAACCTAACTCTTGTTTTTACACTGAAACTCTGAGATAAAACATAA

ATGAATCAAAACTTTCAACAACGGATCTCTTGGTTCTGGCATCGATGAAGAACGCAGCGAAATGCGATAAG

TAATGTGAATTGCAGAATTCAGTGAATCATCGAATCTTTGAACGCACATTGCGCCCTCTGGTATTCCGGAGG

GCATGCCTGTTCGAGCGTCATTTCAACCCTCAAGCCTGGCTTGGTGATGGGGCACTGCCTGTGAAAGGGCA

GGCCCTGAAATACAGTGGCGAGCTCGCCAGGACCCCGAGCGCAGTAGT---TAAACCCTCGC-

TCTGGAAGGCCCTGGCGGTGCCCT-GCCGTTAAA-CCCCCAA-CTCTTGAAA--

--

GAGAAGGAAGGTTAGTAAATACCACAATTGCGGAATATGCTCCCCTGCACTACCATTGCACCTCAAACCGT

CGGCTCGCAGCTGTTCGTGCGGCTTCGTCATATCGGGGGGCGCATTTTCACCCCTCGTTCTGGATTTTCCATT

TTCAGTGCGGGTGCGGGGTGCGCTTATCAGGGGGCTTATCTCCTACTCCCAAAACCCTGTTGCATCACTCAC

GCCCTGCCACTACCAACACCAAAACCCTCAACACCAA-TTCCATCGCTCTCCTACCAT-

ACATGCTTGCAAAACGCATTGAATGCCATGCTGACAATCTATCTACATAGCCGCCG--------------------------------

----

ACCTCGAAGCATCGTTATTGACCTCGACTC-

TTAGGCAAACCATCTCTGGCGAGCACGGCCTCGACAGCAATGGCGTGTATGCACCTCCTATTCCCTGCCCA

CTGATCTTGGCCTCTCTTCCGGCTTGGCACTGACAATTGCACAGTTACAACGGCACTTCTGAGCTCCAGCTC

GAGCGCATGAACGTCTACTTCAACGAGGTATGTCAACAACCACATCATCCATCCGACCATCCTCGACATCC

TTTCCTGCCGTCCTCCGGACCTTGCTAACGCGTTTTCGTCCAGGCTTCCGGCAACAAGTATGTTCCTCGCGC

CGTCCTCGTCGATCTTGAGCCCGGTACTATGGACGCCGT

>KP267887.1 Diaporthe apiculata strain LC3364

CCCTTTGTGAACTTATACCTTA-CCGTTGCCTCGGCG-CCAGCTGGTCCTC--CGGGGCCCCTC--

GGTGTTGAGAGAGCACGCCGGCGGCCAACCTAACTCTTGTTTTTACACTGAAACTCTGAGATAAAACATAA

ATGAATCAAAACTTTCAACAACGGATCTCTTGGTTCTGGCATCGATGAAGAACGCAGCGAAATGCGATAAG

TAATGTGAATTGCAGAATTCAGTGAATCATCGAATCTTTGAACGCACATTGCGCCCTCTGGTATTCCGGAGG

GCATGCCTGTTCGAGCGTCATTTCAACCCTCAAGCCTGGCTTGGTGATGGGGCACTGCCTGTGAAAGGGCA

GGCCCTGAAATACAGTGGCGAGCTCGCCAGGACCCCGAGCGCAGTAGT---TAAACCCTCGC-

TCTGGAAGGCCCTGGCGGTGCCCT-GCCGTTAAA-CCCCCAA-CTCTTGAAA--

- -

GAGAAGGAAGGTTAGTAAATACCACAATTGCGGAATATGCTCCCCTGCACTACCATTGCACCTCAAACCGT

CGGCTCGCAGCTGTTCGTACGGCTTCGTCATATCGGGGGGCGCATTTTCACCCCTCGTTCTGGATTTTCCATT

TTCAGTGCGGGTGCGGGGTGCGCTTATCAGGGGGCTTATCTCCTACTCCCAAAACCCTGTTGCATCACTCAC

GCCCTGCCACTACCAACACCAAAACCCTCAACACCAA-TTCCATCGCTCTCATACAAT-

ACATTCTTGCAAAACGCATTGAATGTCATGCTGACAATCTATCTACATAGCCGCCG-------------------------------

----

ACCTCGAAGCATCCTTACTGACCTCGACTC-

TTAGGCAAACCATCTCTGGCGAGCACGGCCTCGACAGCAATGGCGTGTATGCACCTCCTATTCCCTGCCCA

CTGATCTTGGCCTCTCTTCCGGCTTGGCACTGACAATTGCACAGTTACAACGGCACTTCTGAGCTCCAGCTC

GAGCGCATGAACGTCTACTTCAACGAGGTATGTCAACAACCACATCATCCATCCGACCATGCTCAACATCT

TTTGTTGCTGTCTTCTAGACCTTGCTAACGCGTTATCGTCCAGGCTTCCGGCAACAAGTATGTTCCTCGCGCC

GTCCTCGTCGATCTCGAGCCCGGTACTATGGACGCCGT

>KC343032.1 Diaporthe arecae strain CBS 161.64

CCCTTTGTGAACTTATACCTTA-CTGTTGCCTCGGCG-CATGCTGGCCCCC-

TCGGGGCCCCCTGAGACAGGGAGCAGGCACGCCGGCGGCCAAGTTAACTCTTGTTTTTACACTGAAACTCT

GAGAAAAAACACAAATGAATCAAAACTTTCAACAACGGATCTCTTGGTTCTGGCATCGATGAAGAACGCA

GCGAAATGCGATAAGTAATGTGAATTGCAGAATTCAGTGAATCATCGAATCTTTGAACGCACATTGCGCCC

TCTGGTATTCCGGAGGGCATGCCTGTTCGAGCGTCATTTCAACCCTCAAGCATTGCTTGGTGTTGGGGCACT

GCTTTTTACCGAGCAGGCCCTGAAATCTAGTGGCGAGCTCGCCAGGACCCCGAGCGCAGTAGT---

TAAACCCTCGC-TCTGGAAGGCCCTGGCGGTGCCCT-GCCGTTAAA-CCCCCAA-

CTTCTGAAAATTTGACCTCGGATCAGGTAGGAATACCCGCTGAACTTAAGCATAGAGAAGGAAGGTTAGTA

AACATCACAATCATAAGACGCTCTCCTCTGCCCTCACCCTTTGCCTCAGATCATCAGTGTGCGGCTGTTGGT

ACAGCTTCGTCATACTGAGGGGCGCATTTTCACCCCTCGCTCTGGATTTTCCATTTTCAGTGCGGGTGCGGG

GTGCGCTTATCAGGGAGCTTATCTCCTACACCCAAAACCCTGTTGCAACAACAACTCCTTGCAGTTATTACC

ACCACTACATCCACCCTCAA-CCCCATCGACATCCTTAAAT--CAATCAAGCAAACC---

CAGGAAATCATGCTGACTTTCTATCTACATAGCCGCCGAGCTTGGTAAGGGTTTGCGTAAGTTGCT-

CCCGTCAACACCTC-

CAGACCTTATCATCGCCACCCGTAGCTGACACGTTTCCCAGGGTAACCAAATCGGTGCTGCTTTCTGGTGC--

GTCC-------------------ACCACCGCGAGCTC-
GACGCGCGACAATACGATCTCGAACCATCGTTACTGACCTCGACTC-
TCAGGCAAACCATCTCTGGCGAGCACGGCCTCGACAGCAATGGCGTGTATGCACCTCCTATTTCCTGCCTTC
AGATCTCGTCCTCCCTGCCGGCTTGGCACTGACAATCATACAGTTACAACGGCACTTCCGAGCTCCAGCTCG
AGCGCATGAACGTCTACTTCAACGAGGTAAGCCAACAGCCACGTCG-----
TTGACCATGTGCAGCACGATTTCCTGCCGTCGCCAGG-
CTTTGCTGACGCGTTATCGCACAGGCCTCCGGCAACAAGTATGTCCCTCGCGCCGTCCTCGTCGATCTCGAG
CCCGGTACCATGGACGCCGT

>KC343034.1 Diaporthe arengae strain CBS 114979

CCCTTTGTGAACTTATACC-TT-TTGTTGCCTCGGCG-CATGCTGGCCTCT-AGTAGGCCCCTC--
GGTGAGGAGAAGGCACGCCGGCGGCCAAGTTAACTCTTGTTTTTACACTGAAACTCTGAGAAAAAACACA
AATGAATCAAAACTTTCAACAACGGATCTCTTGGTTCTGGCATCGATGAAGAACGCAGCGAAATGCGATAA
GTAATGTGAATTGCAGAATTCAGTGAATCATCGAATCTTTGAACGCACATTGCGCCCTCTGGTATTCCGGAG
GGCATGCCTGTTCGAGCGTCATTTCAACCCTCAAGCATTGCTTGGTGTTGGGGCACTGCTTCTAACGAAGCA
GGCCCTGAAATCTAGTGGCGAGCTCGCCAGGACCCCGAGCGCAGTAGT---TAAACCCTCGC-
TCTGGAAGGCCCTGGCGGTGCCCT-GCCGTTAAA-CCCCCAA-
CTTCTGAAAATTTGACCTCGGATCAGGTAGGAATACCCGCTGAACTTAAGCATAGAGAAGGAAGGTTAGTA
AACATCACAATCATAAAACATGCTCTCCTGCCCTCACCTTTTGCCTCAAATCATCAGTGTGCGGCTGTTGGT
ACAGCTTCGTCATACTGAGGGGCGCATTTTCACCCCTCGCTCTGGATTTTCCATTTTCAGTGCGGGTGCGGG
GTGCGCTTATCAGGGAGCTTATCTCCTACACCCAAAACCCTGTTGCAACAACAACTCCTTGCAGTTACTACC
ACCACTTCATTCACCCTCAACCCCCATCGACATCCTTGAAT-CAA-TCAAGCAAACCGCCAGG-
AAATCATGCTGACTTTCTATCTACATAGCCGCCGAGCTTGGTAAGGGTTTGCGTAAGTTGCT-
CCTGTCAACACCGA-
CGGACCTTATCATCGCCACCCGTAGCTGACACGTTTCCCAGGGTAACCAAATCGGTGCTGCTTTCTGGTGC--
GTCC-------------------ACCACCGCGAGCTC-
GACGCGCGACAATACGACCTCGAAACATCGTTACTGACCTCGACTC-
TCAGGCAAACCATCTCTGGCGAGCACGGCCTCGACAGCAATGGCGTGTATGCACCTCCTATTCCCTACCTTC
AGATCTCGTCCTCCCTGCCGGCTTGGCACTGACAATCATACAGTTACAACGGCACTTCCGAGCTCCAGCTCG
AGCGCATGAACGTCTACTTCAACGAGGTAAGTCAACAGCCACGTCG-----
TTGGCCATCTGCAGCACGGTTTGCTGCCGTCGCTGGGCCCTTGCTAACGCGTTATCGCACAGGCCTCCGGCA
ACAAGTATGTCCCTCGCGCCGTCCTCGTCGATCTCGAGCCCGGTACCATGGACGCCGT

>KT459414.1 Diaporthe aseana voucher MFLUCC 12-0299a

CCCTTTGTGAACTTATACCTTA-CTGTTGCCTCGGCG-
CATGCTGGCCCCCCTGGGGGTCCCTCGAGACGAGGAGCAGGCACGCCGGCGGCCAAGTTAACTCTTGTTTT
TACACTGAAACTCTGAGAAAAAACACAAATGAATCAAAACTTTCAACAACGGATCTCTTGGTTCTGGCATC
GATGAAGAACGCAGCGAAATGCGATAAGTAATGTGAATTGCAGAATTCAGTGAATCATCGAATCTTTGAAC
GCACATTGCGCCCTCTGGTATTCCGGAGGGCATGCCTGTTCGAGCGTCATTTCAACCCTCAAGCATTGCTTG
GTGTTGGGGCACTGCCTTTAAACGGGCAGGCCCTGAAATCTAGTGGCGAGCTCGCCAGGACCCCGAGCGCA
GTAGT---TAAACCCTCGC-TCTGGAAGGCCCTGGCGGTGCCCT-GCCGTTAAA-CCCCCAA-
CTCTTGAAAATTTGACCTCGGATCAGGTAGGAATACCCGCTGAACTTAAGCATAGAGAAGGAAGGTTAGTA
AACATCACAATCATAGAACATGCTACCCTATCCCCACACTTTGCCTCAAATCATCAGTGTGCGGCTGTTGGT
ATGGCTTCGTCATACTGAGGGGCGCATTTTCACCCCTCGCTTTGGATTTTCCA-
TTTCAGTGCGGGTGCGGGGTGCGCTTATCAGGGAGCTTATCTCCTACAC-
CAAAACCCTGTTGCAACAACAACTCCTTGCCGCTACTACCACCACTTCATTCACCCTCAACCCTCATCGGCA
CCCTTCAAT-CGA-
TCAAGTAAACGGTCGCGAAAATCATGCTGACGCTTTTTCTACACAGCCGCCGAGCTTGGTAAGGGT----------
---AACGTTCTTCTGTGGTCCTAGTCC------------------
CCACT-CGAGCTC-GACGCGCGACAATACGAT-TCGATCCATCGTCACTGACCTCGACTC-
TCAGGCAAACCATCTCTGGCGAGCACGGCCTCGACAGCAATGGCGTGTATGCACCTCCTATTCCCTACCTCC
AGATCTCGTCCTCCCTGCCGGCTTGGCATTGACAATCATACAGTTACAACGGCACTTCCGAGCTCCAGCTCG
AGCGCATGAACGTCTACTTCAACGAGGTAAGTCAACAGCCACGTCG-----
TTGACCATCTGCAGCACGGTTTCCTGCCGTCGCCAAGGCTTTGCTAACGCGTTATCGCCCAGGCCTCCGGCA
ACAAGTATGTTCCTCGCGCCGTCCTTGTCGATCTCGAGCCCGGTACCATGGACGCCGT

>MN708222.1 Diaporthe australiana strain BRIP 66145

CCCTTTGTGAACTTATACCTTA-CTGTTGCCTCGGCG-CATGCTGGCCCCC-TGGGGGTCCCTC--
GATGAGGAGCAGGCACGCCGGCGGCCAAGTTAACTCTTGTTTTTACACTGAAACTCTGAGAAAAAACACAA
ATGAATCAAAACTTTCAACAACGGATCTCTTGGTTCTGGCATCGATGAAGAACGCAGCGAAATGCGATAAG
TAATGTGAATTGCAGAATTCAGTGAATCATCGAATCTTTGAACGCACATTGCGCCCTCTGGTATTCCGGAGG
GCATGCCTGTTCGAGCGTCATTTCAACCCTCAAGCACTGCTTGGTGTTGGGGCACTGCCTTTCAAG-
GGCAGGCCCTGAAATCTAGTGGCGAGCTCGCCAGGACCCCGAGCGCAGTAGT---TAAACCCTCGC-
TCTGGAAGGCCCTGGCGGTGCCCT-GCCGTTAAA-CCCCCAA-
CTCTTGAAAATTTGACCTCGGATCAGGTAGGAATACCCGCTGAACTTAAGCATA-----------------------------
AGAACATGCCACCCTGTCCTCACATTTTGCCTCAAATCATCAGTGTGCGGCTGTTGGTACGGCTTCGTCATA
CTGAGGGGCGCATTTTCACCCCTCGCTCTGGTTTTTCCA-
TTTCAGTGCGGGTGCGGGGTGCGCTTATCAGGGAGCTTATCTCCTACACCCAAAACCCTGTTGCAACAATA
ACACCATGCTGTTCCTT---------CATTCACCCTCAAACCTCATCGATATCCTTGAAT--

T G A T C A A G C A A A C C G C C T C G A A -
ATCATGCTGACATCGTATCTACACAGCCGCCGAGCTTGGCAAGGGTTTGCGTAAGTTGCT-
C C T G T C A A C A C C G C -
CGGACCTTATCATCGCTACCCGTAGCTGACACGTTTCGCAGGGTAACCAAATCGGTGCTGCTTTCTGGTGC--
G T C C - A C C A C C G C G A G C T C -
G A C G C G C G A C A A T A C G A C C T C G A A C C A T C G T T A C T G A C C T C G A C T C -
TCAGGCAAACCATCTCTGGCGAGCACGGCCTCGACAGCAATGGCGTGTATGCACCTCCTATTCCCTACCTCC
AGATCTCGTCCTCCCTGCCGGCTTGGCACTGACAATCATACAGTTACAACGGCAGTTCCGAGCTCCAGCTCG
A G C G C A T G A A T G T C T A C T T C A A C G A G G T A A G T A A A C A G C C A C G T C G - - - - -
TTGACCATGTGCAGCACGGTTTCCTGCCGTCGCCAAGGCCTTGCTAACGCGTTATCGCCCAGGCCTCCGGCA
ACAAGTATGTCCCTCGCGCCGTCCTCGTCGATCTCGAGCCCGGTACCATGGACGCCGT

>KT732950.1 Diaporthe betulae strain CFCC 50469

CCCTTTGTGAACTTATACCTTA-CTGTTGCCTCGGCG-CACGCCGGCCCCC-TTGGGGGCCCCT-----
GAGGAGCAGGCACGCCGGCGGCCAACCTAACTCTTGCTTTTACACTGAAACTCTGAGATAAAACATAAATG
AATCAAAACTTTCAACAACGGATCTCTTGGTTCTGGCATCGATGAAGAACGCAGCGAAATGCGATAAGTAA
TGTGAATTGCAGAATTCAGTGAATCATCGAATCTTTGAACGCACATTGCGCCCTCTGGTATTCCGGAGGGC
ATGCCTGTTCGAGCGTCATTTCAACCCTCAAGCCTGGCTTGGTGATGGGGCACTGCCTGTAAAAGGGCAGG
C C C T G A A A T T C A G T G G C G A G C T C G C C A G G A C C C C G A G C G C A G T A G T - - - T A A A C C C T C G C -
TTTGGAAGGCCCTGGCGGTGCCCT-GCCGTTAAA-CCCCCAA-CTTCTGAAAATTT-----------------------------------
- - - - -
GAGAAGGAAGGTTAGTAAATACCACAGTCACGGAACATGCCACCCTGCCCTCCTACTGCACCTCAAGTCAT
CAGCCCGCAGCTGCTCGCGCGGCTTCGTCATGTCGGGGGGCGCATTTTCACCCCTCGCTTTGGATTTTCCAT
TTTCAGTGCGGGTGCGGGGTGCGCTTATCAGGGGGCTTATCTCCTACACCCAAAACCCTGTTGCATTACTCA
CC-CTCGCCACCACCACCACCAATACGCTCACCATCAA-TCCCATCG-CCCTTTTCAAT-ACA-
ACCCTTGAAACGCGTCGAACATTATGCTGACTCTCTACTTACACAGCCGCCGAGCTTGGCAAGGGTTTGCGT
A A G T T G C T - C C T G T C A A C A C C A G -
CGGACCTTATCATCGCCACCTGTAGCTGACACGTTTCCCAGGGTAACCAAATCGGTGCTGCTTTCTGGTGCG
T T C C A G C T -
CGAGCTCCAAGTTCACCGCCGCGACGCTCGACACGCGACAATACGACCTCGAAGCATCGTTGCTGACCTCG
A C T T -
TTAGGCAAACCATCTCTGGCGAGCACGGCCTCGACAGCAATGGCGTGTATGCACCTCCTATTCCCTGCCCA
CTGATCTTGGCCTCTCTTCCGGCTTGGCACTGACAATCGCACAGTTACAACGGCACTTCTGAGCTCCAGCTC
GAGCGCATGAACGTCTACTTCAACGAGGTAAGTCAACAACCACATCATCCATCCGACCATCTCCAACACGG
T T T A C T G C C A T C G C C -

GGACCTCGCTAACGCGTCATCGCCCAGGCCTCTGGCAACAAGTATGTTCCTCGCGCCGTCCTCGTCGATCTC
GAGCCCGGTACCATGGACGCCGT

>MW882216.1 Diaporthe sp. isolate HHL19101101S2D1

C C C T T T G T G A A C T T A T A C C - C A - C T G T T G C C T C G G C G - C A G G C C G G T C T G T -
G T C A G A C C C C C T G A G A C A G G G A G C A - G C C C G C C G G C G G C C A A C C A A A C T C -
TGTTTCTATAGTGAATCTCTGAGAAAAAACATAAATGAATCAAAACTTTCAACAACGGATCTCTTGGTTCTG
GCATCGATGAAGAACGCAGCGAAATGCGATAAGTAATGTGAATTGCAGAATTCAGTGAATCATCGAATCTT
TGAACGCACATTGCGCCCTCTGGTATTCCGGAGGGCATGCCTGTTCGAGCGTCATTTCAACCCTCAAGCCTG
GCTTGGTGATGGGGCAGTGCCTTGGACAAGGCACGCCCTGAAATTCAGTGGCGAGCTCGCCAGGACCCCGA
GCGCAGTAGT---TACA-TCTCGC-TCTGGAAGGCCCTGGCGGTGCCCT-GCCGTTAAA-CCCCCAA-
CTTCTGAAAATTTGACCTCGGATCAGGTAGGAACACCAGCTGAACTTAAGCATAGAGAAGGAAGCTCACG
AAACATTAAACTCACAACATCAACCCCAAGTCATCCCAGTGCACATCGCGATCATCGGCGCGCGACCGTCT
G C G C C G C T T C G T C A C T C - - -
GTCACGCATTTTCACCCCTCGTTCTGGATTTTCCACTTTCAGTGCGGGTGCGGGGTGTGCTTATCAGGCCGC
T T A T C G C T T G C G T T C A A A A C C C T G T G G C A T C A A C C A C T C C T T G C C G C T G C - -
A C A T T A A C G C C A A C G C C A T C A A - T C T C A T C A C C C C A C C C C C - - - - - - -
TCTGACAAGACCTGAGGAAAACCATGCTGACTCGCTTTCCATACAGCCGCCGAGCTGGGCAAGGGTTTGCG
T A A G T T G C T C C C T G T C A A C A C C G C - C C G A C C T T A T C G C C A C C C - - -
GTAGCTGACACGTTTCCCAGGGTAACCAAATCGGTGCTGCTTTCTGGTGCGTCCCAGCTCCCAGCTCCAGGC
CTACTGCCGCGATGCTCGACGCGCGACAAGACCAGCTCGCAACAGTGTTACTGACCTAGGCTCTGTAGGCA
AACCATCTCTGGCGAGCACGGCCTCGACAGCAATGGCGTGTATGCACCTCCTATTCCCTGCCCACTGATCCC
G T C C T G T C C T C - -
GCTTGGCACTGATGATTGCACAGTTACAACGGCACTTCCGAGCTCCAGCTCGAGCGCATGAACGTCTACTT
CAACGAGGTATGTCAATGGCCACGTCGTCAATCCAAACATCTACTGCGCCGCGTGCTGCCGTAGCCAAAGC
CTTGCTAACGCGTTGTCGCCCAGGCTTCCGGCAACAAGTATGTGCCCCGCGCTGTCCTCGTCGATCTCGAGC
CCGGTACCATGGACGCCGT

>MW882217.1 Diaporthe sp. isolate HHL19101101S2D2

C C C T T T G T G A A C T T A T A C C - C A - C T G T T G C C T C G G C G - C A G G C C G G T C T G T -
G T C A G A C C C C C T G A G A C A G G G A G C A - G C C C G C C G G C G G C C A A C C A A A C T C -
TGTTTCTATAGTGAATCTCTGAGAAAAAACATAAATGAATCAAAACTTTCAACAACGGATCTCTTGGTTCTG
GCATCGATGAAGAACGCAGCGAAATGCGATAAGTAATGTGAATTGCAGAATTCAGTGAATCATCGAATCTT
TGAACGCACATTGCGCCCTCTGGTATTCCGGAGGGCATGCCTGTTCGAGCGTCATTTCAACCCTCAAGCCTG

GCTTGGTGATGGGGCAGTGCCTTGGACAAGGCACGCCCTGAAATTCAGTGGCGAGCTCGCCAGGACCCCGA

GCGCAGTAGT---TACA-TCTCGC-TCTGGAAGGCCCTGGCGGTGCCCT-GCCGTTAAA-CCCCCAA-

CTTCTGAAAATTTGACCTCGGATCAGGTAGGAATACCAAACGAACTTAAGCATAGAGAAGGAAGGTCAGT

AAACATTAAACTCACAACATCAACCCCAAGTCATCCCAGTGCACATCGCGATCATCGGCGCGCGACCGTCT

GCGCCGCTTCGTCACTC---

GTCACGCATTTTCACCCCTCGTTCTGGATTTTCCACTTTCAGTGCGGGTGCGGGGTGTGCTTATCAGGCCGC

TTATCGCTTGCGTTCAAAACCCTGTGGCATCAACCACTCCTTGCCGCTGC--

ACATTAACGCCAACGCCATCAA-TCTCATCACCCCACCCCC-------

TCTGACAAGACCTGAGGAAAACCATGCTGACTCGCTTTCCATACAGCCGCCGAGCTGGGCAAGGGTTTGCG

TAAGTTGCTCCCTGTCAACACCGC-CCGACCTTATCGCCACCC---

GTAGCTGACACGTTTCCCAGGGTAACCAAATCGGTGCTGCTTTCTGGTGCGTCCCAGCTCCCAGCTCCAGGC

CTACTGCCGCGATGCTCGACGCGCGACAAGACCAGCTCGCAACAGTGTTACTGACCTAGGCTCTGTAGGCA

AACCATCTCTGGCGAGCACGGCCTCGACAGCAATGGCGTGTATGCACCTCCTATTCCCTGCCCACTGATCCC

GTCCTGTCCTC--

GCTTGGCACTGATGATTGCACAGTTACAACGGCACTTCCGAGCTCCAGCTCGAGCGCATGAACGTCTACTT

CAACGAGGTATGTCAATGGCCACGTCGTCAATCCAAACATCTACTGCGCCGCGTGCTGCCGTAGCCAAAGC

CTTGCTAACGCGTTGTCGCCCAGGCTTCCGGCAACAAGTATGTGCCCCGCGCTGTCCTCGTCGATCTCGAGC

CCGGTACCATGGACGCCGT

>MW882218.1 Diaporthe sp. isolate HHL19101101S2D3

CCCTTTGTGAACTTATACC-CA-CTGTTGCCTCGGCG-CAGGCCGGTCTGT-

GTCAGACCCCCTGAGACAGGGAGCA-GCCCGCCGGCGGCCAACCAAACTC-

TGTTTCTATAGTGAATCTCTGAGAAAAAACATAAATGAATCAAAACTTTCAACAACGGATCTCTTGGTTCTG

GCATCGATGAAGAACGCAGCGAAATGCGATAAGTAATGTGAATTGCAGAATTCAGTGAATCATCGAATCTT

TGAACGCACATTGCGCCCTCTGGTATTCCGGAGGGCATGCCTGTTCGAGCGTCATTTCAACCCTCAAGCCTG

GCTTGGTGATGGGGCAGTGCCTTGGACAAGGCACGCCCTGAAATTCAGTGGCGAGCTCGCCAGGACCCCGA

GCGCAGTAGT---TACA-TCTCGC-TCTGGAAGGCCCTGGCGGTGCCCT-GCCGTTAAA-CCCCCAA-

CTTCTGAAAATTTGACCTCGGATCAGGTAGGAATACCAGCCAAACTTAAGCATAGAGAAGGAAGGTCAGT

AAACATTAAACTCACAACATCAACCCC-

AAGTCATCCCAGTGCACACGCGATCATCGGCGCGCGACCGTCTGCGCCGCTTCGTCACTC---

GTCACGCATTTTCACCCCTCGTTCTGGATTTTCCACTTTCAGTGCGGGTGCGGGGTGTGCTTATCAGGCCGC

TTATCGCTTGCGTTCAAAACCCTGTGGCATCAACCACTCCTTGCCGCTGC--

ACATTAACGCCAACGCCATCAA-TCTCATCACCCCACCCCC-------

TCTGACAAGACCTGAGGAAAACCATGCTGACTCGCTTTCCATACAGCCGCCGAGCTGGGCAAGGGTTTGCG

TAAGTTGCTCCCTGTCAACACCGC-CCGACCTTATCGCCACCC---

GTAGCTGACACGTTTCCCAGGGTAACCAAATCGGTGCTGCTTTCTGGTGCGTCCCAGCTCCCAGCTCCAGGC
CTACTGCCGCGATGCTCGACGCGCGACAAGACCAGCTCGCAACAGTGTTACTGACCTAGGCTCTGTAGGCA
AACCATCTCTGGCGAGCACGGCCTCGACAGCAATGGCGTGTATGCACCTCCTATTCCCTGCCCACTGATCCC
G T C C T G T C C T C - -
GCTTGGCACTGATGATTGCACAGTTACAACGGCACTTCCGAGCTCCAGCTCGAGCGCATGAACGTCTACTT
CAACGAGGTATGTCAATGGCCACGTCGTCAATCCAAACATCTACTGCGCCGCGTGCTGCCGTAGCCAAAGC
CTTGCTAACGCGTTGTCGCCCAGGCTTCCGGCAACAAGTATGTGCCCCGCGCTGTCCTCGTCGATCTCGAGC
CCGGTACCATGGACGCCGT

>KJ490597.1 Diaporthe biconispora isolate ZJUD62

C C C T T T G T G A A C T T A T A C C T T A - C T G T T G C C T C G G C G - T A C G C T G G C C C C T - -
AGGGGTCCCTTGAGACAAGGAGCAGGCGCGCCGGCGGCCAAGTTAACTCTTGTTTTTACACTGAAACTCTG
A G A A A A A A C A -
AAATGAATCAAAACTTTCAACAACGGATCTCTTGGTTCTGGCATCGATGAAGAACGCAGCGAAATGCGATA
AGTAATGTGAATTGCAGAATTCAGTGAATCATCGAATCTTTGAACGCACATTGCGCCCTCTGGTATTCCGGA
GGGCATGCCTGTTCGAGCGTCATTTCAACCCTCAAGCCTGGCTTGGTGTTGGGGCACTGCTTCTCGGGAAGC
AGGCCCTCAAATCTAGTGGCGAGCTCGCCAGGACCCCGAGCGTAGTAGT---TAAACCCTCGC-
TTTGGAAGGCCCTGGCGGTGCCCT-GCCGTTAAA-CCCCCAA-CTTTGAAAATT-------------------------------------
- - - -
GAGAAGGAAGGTTAGTAAACATCACAATCACAGAATATGCTACCCTGCCCTTCCACTCTGCCTCAAGTCAT
CATCGTGCGGCTGTTCGTGTGGCTTCGTCATACTGAGGGGCGCATTTTCACCCCTCGCTCTGGATTTTCCATT
TTCAGTGCGGGTGCGGGGTGCGCTTATCAGGGAGCTTATCTCCTACACCCAAAACCCTGTTGCAACAAATG
CCCACCAACACCATCACCACCCATTCGTCCCCCCTCAA-TCCCACCGCCCTCCTACAAT-ACA-
GCATGTAAATGGCATTGAAAATCATGCTAACTTTTCACCTCCTCAGCCGCCGAGCTTGGTAAGGGTT----------
----
A C C T C G A A G C A T C G T T A C T G A C C T C G A C T C -
TTAGGCAAACCATCTCTGGCGAGCACGGCCTCGACAGCAATGGCGTGTATGCACCTCCTATTCCCTGTCCAT
TGATTCTGTCCTCTCCTCCGGCTTGGCACTGACAATCATACAGTTACAACGGCACTTCCGAGCTCCAGCTCG
A G C G C A T G A A C G T C T A C T T C A A C G A G G T A A G T C A A C A G C C A C G T C G - - - - -
CTGACCATCTACACCACGATTTCCCGCCGTCGCCGGGGCTTTGCTAACGCGTTATCGCCCAGGCCTCCGGCA
ACAAGTATGTTCCTCGCGCCGTCCTCGTCGATCTCGAGCCCGGTACCATGGACGCCGT

>MN901242.1 Diaporthe biconispora isolate YJ1

CCCTTTGTGAACTTATACCTTA-CTGTTGCCTCGGCG-TACGCTGGCCCCC-AGGAAGTCCCTC--
GGTGAGGAGCAGGCGCGCCGGCGGCCAAGTTAACTCTTGTTTTTACACTGAAACTCTGAGAAAAAAACAA
AATGAATCAAAACTTTCAACAACGGATCTCTTGGTTCTGGCATCGATGAAGAACGCAGCGAAATGCGATAA
GTAATGTGAATTGCAGAATTCAGTGAATCATCGAATCTTTGAACGCACATTGCGCCCTCTGGTATTCCGGAG
GGCATGCCTGTTCGAGCGTCATTTCAACCCTCAAGCCTGGCTTGGTGTTGGGGCACTGCCTGTAAAAGGGC
AGGCCCTCAAATCTAGTGGCGAGCTCGCCAGGACCCCGAGCGTAGTAGT---TAAACCCTCGC-
TTTGGAAGGCCCTGGCGGTGCCCT-GCCGTTAAA-CCCCCAA-
CTTTTGAAAATTTGACCTCGGATCAGGTAGGAATACCCGCTGAACTTAAGCATA----------------------------------
- - - - - - - - - -
GCCCTTCCACTCTGCCTCAAGTCATCATCGTGCGGCTGTTCGTGTGGCTTCGTCATACTGAGGGGCGCATTT
TCACCCCTCGCTCTGGATTTTCCATTTTCAGTGCGGGTGCGGGGTGCGCTTATCAGGGAGCTTATCTCCTAC
ACCCAAAACCCTGTTGCAACAAATGCCCACCAACACCATCACCACCCATTCGTCCACCCTCAA-
TCCCACCGCCCTCCTACAAT-ACA-
GCATGTAAATGGCATTGAAAATCATGCTAACTTTTCACCTACTCAGCCGCCGAGCTTGGTAAGGGTC----------
----
CTCGACGCGCGACAATACGACCTCGAAGCATCGTTGCTGACCTCGACTC-
TTAGGCAAACCATCTCTGGCGAGCACGGCCTCGACAGCAATGGCGTGTATGCACCTCCTATTCCCTGCCCAT
TGATTTCGTCCTCTCCTCCGGCTTGGCACTGACAATCACACAGTTACAACGGCACTTCCGAGCTCCAGCTCG
AGCGCATGAACGTCTACTTCAACGAGGTAAGTCAACAGCCACGTCG-----
CTGACCATCTACACCACGATTTCTCGCCGTCGCCGGGGCTTTGCTAACGCGTTATCGCCCAGGCATCCGGCA
ACAAGTATGTTCCCCGCGCCGTCCTCGTCGATCTCGAGCCCGGTACCATGGACGCCGT

>KJ490582.1 Diaporthe biguttulata isolate ZJUD47

CCCTTTGTGAACTTATACCTTA-CTGTTGCCTCGGCG-
CAGGCCGGCCTCTGCTGAGGCCCCCGAGACGGGGAGCA-
GCCCGCCGGCGGCCAACCTAACTCTTGTTTTTACACTGTATCTCTGAGAAAAAACATAAATGAATCAAAAC
TTTCAACAACGGATCTCTTGGTTCTGGCATCGATGAAGAACGCAGCGAAATGCGATAAGTAATGTGAATTG
CAGAATTCAGTGAATCATCGAATCTTTGAACGCACATTGCGCCCTCTGGTATTCCGGAGGGCATGCCTGTTC
GAGCGTCATTTCAACCCTCAAGCCTGGCTTGGTGTTGGGGCACTGCTTCGAGAGAAGCAGGCCCTGAAATC
TAGTGGCGAGCTCGCCAGGACCCCGAGCGTAGTAGT---TATA-TCTCGT-
TCTGGAAGGCCCTGGCGGTGCCCT-GCCGTTAAA-CCCCCAA-CTTCTGAAAATT-----------------------------------
-----GAGAAGGAAGGTTAGTAAACATCA-AGTCACAGCATAAACTCC-
CTTGCGTCCTATGCACCTCCGAATTATCGGCGCGCGGCCATCTGCGCGGCTTCGTCACATCTTGAAG-
GCATTTTCACCCCTCGCTCTGGATTTTCCATTTTCAGTGCGGGTGCGGGGTGCGCTTATCAGGCGGCTTATCT
CTTACACCCAAAACCCTGTTGCATCCACCACATGCCGCTACCACCAATACCATCATAATCATCTTCAA-

G C A C A A C A A T -

CGAAATGCGCTGGAACATATGCTGACCATCTTTCTACACAGCCGCCGAGCTTGGTAAGGGT---------------------

----

A G C T C G C A G C A T C G T T A C T G A C A T C T G C T T T -

CAGGCAAACCATCTCTGGCGAGCACGGCCTCGACAGCAATGGCGTGTATGCACCTCCTATTCCCTGCCCAT

T G A T C T C G T C C T C T C C T C - -

GCTTGGCACTGACAATTGCACAGTTACAACGGCACTTCCGAGCTCCAGCTCGAGCGCATGAACGTCTACTT

CAACGAGGTAAGTCAACAGCCACGTCGTCAATTCGAGCATCTACGGCATGGTTTCCTGCCGCCGCCAAGGT

CTTGCTAACGCGTTATCTCCCAGGCCTCCGGCAACAAGTATGTGCCTCGCGCTGTCCTCGTCGATCTCGAGC

CCGGTACCATGGACGCCGT

>KJ490583.1 Diaporthe biguttulata isolate ZJUD48

C C C T T T G T G A A C T T A T A C C T T A - C T G T T G C C T C G G C G -

C A G G C C G G C C T C T G C T G A G G C C C C C C G A G A C G G G G A G C A -

GCCCGCCGGCGGCCAACCTAACTCTTGTTTTTACACTGTATCTCTGAGAAAAAACATAAATGAATCAAAAC

TTTCAACAACGGATCTCTTGGTTCTGGCATCGATGAAGAACGCAGCGAAATGCGATAAGTAATGTGAATTG

CAGAATTCAGTGAATCATCGAATCTTTGAACGCACATTGCGCCCTCTGGTATTCCGGAGGGCATGCCTGTTC

GAGCGTCATTTCAACCCTCAAGCCTGGCTTGGTGTTGGGGCACTGCTTCGAGAGAAGCAGGCCCTGAAATC

T A G T G G C G A G C T C G C C A G G A C C C C G A G C G T A G T A G T - - - T A T A - T C T C G T -

TCTGGAAGGCCCTGGCGGTGCCCT-GCCGTTAAA-CCCCCAA-CTTCTGAAAATT------------------------------------

- - - - - G A G A A G G A A G G T T A G T A A A C A T C A - A G T C A C A G C A T A A A C T C C -

CTTGCGTCCTATGCACCTCCGAATTATCGGCGCGCGGCCATCTGCGCGGCTTCGTCACATCTTGAAG-

GCATTTTCACCCCTCGCTCTGGATTTTCCATTTTCAGTGCGGGTGCGGGGTGCGCTTATCAGGCGGCTTATCT

CTTACACCCAAAACCCTGTTGCATCCACCACATGCCGCTACCACCAATACCATCATAATCATCTTCAA-

G C A C A A C A A T - C G A A A T G C G C T G G A -

ACAATGCTGACCATCTTTCTACACAGCCGCCGAGCTTGGTAAGGGTT--

- -

A G C T C G C A G C A T C G T T A C T G A C A T C T G C T T T -

CAGGCAAACCATCTCTGGCGAGCACGGCCTCGACAGCAATGGCGTGTATGCACCTCCTATTCCCTGCCCAT

T G A T C T C G T C C T C T C C T C - - G C T T G G C A C T G A C A A T -

GCACAGTTACAACGGCACTTCCGAGCTCCAGCTCGAGCGCATGAACGTCTACTTCAACGAGGTAAGTCAAC

AGCCACGTCGTCAATTCGAGCATCTACGGCATGGTTTCCTGCCGCCGCCAAGGTCTTGCTAACGCGTTATCT

CCCAGGCCTCCGGCAACAAGTATGTGCCTCGCGCTGTCCTCGTCGATCTCGAGCCCGGTACCATGGACGCC

GT

>MG281015.1 Diaporthe bohemiae strain CBS 143347

C C C T T T G T G A A C T C A T A C C C T A - C T G T T G C C T C G G C G -
CAGGCCGGCCCCCCCGGGGGCCCCTCGAGACGAGGAGCAGGCCGGCCGGCGGCCTAGTTAACTCTTGTTTT
TACACTGAAACTCTGAGAATAAACATAAATGAATCAAAACTTTCAACAACGGATCTCTTGGTTCTGGCATC
GATGAAGAACGCAGCGAAATGCGATAAGTAATGTGAATTGCAGAATTCAGTGAATCATCGAATCTTTGAAC
GCACATTGCGCCCTCTGGTATTCCGGAGGGCATGCCTGTTCGAGCGTCATTTCAACCCTCAAGCCTGGCTTG
GTGATGGGGCACTGCCTGTAAAAGGGCAGGCCCTGAAATTCAGTGGCGAGCTCGCCAGGACCCCGAGCGC
AGTAGT---TAAACCCTCGC-TCTGGAAGGCCCTGGCGGTGCCCT-GCCGTTAAA-CCCCCAA-
CTTCTGAAAATTTGACCTCGGATCAGGTAGGAATACCCGCTGAACTTAAGCATAGAGAAGGAAGGTTAGTA
AACATCACAATCACAGCACATGCTATCTTGCCCTCCCACCTCGAATCGAAACATCGGCGCGCGGCGGCCTG
CGCAGCTCCGTGAGACACGGGGGCGCATTTTCACCCCACGTCCTGGATTTTCCATTTTCAGTGCGGGTGCGG
GGTGCGCTTATCAGGGAGCTTATCTCCCACTCCCAAAACCCTGCTGCACCACT----
CCTTGCCACTACCACCACCA------TCACCATTCA-TCCTACTGCCATCTTCGAACACCA------
CAGAAAGCGCTGGAAATGATGCTAACAATCCAACTATATAGCCGCCGAGCTTGGCAAGGGTT-----------------
---AACCAAATCGGTGCTGCTTTCTGGTGCGTCCC-------
AGCTCCAAACTCACCACCGCGACGCTCGACGCGCGACAACACGGCCTCGCGGCATCGTTACTGACCTCGAC
T C T T -
AGGCAAACCATCTCTGGCGAGCACGGCCTCGACAGCAATGGCGTGTATGCACCTCCTATTCCCTGCACACA
GATCTCGCCCTCTCCTCCGGCTTGGCACTGACAGTTGCACAGTTACAACGGCACTTCCGAGCTCCAGCTCGA
GCGCATGAACGTCTACTTCAACGAGGTATGTCAACAACCATATTGTCCCATCCGACCGTTTCAGCACGGTTT
CCTATCGTCGTCCGGACCTCGCTGACGCGTTATCGCCCAGGCCTCCGGCAACAAGTATGTTCCTCGTGCGGT
CCTCGTCGATCTCGAGCCCGGTACCATGGACGCCGT

>MG281016.1 Diaporthe bohemiae strain CBS 143348

C C C T T T G T G A A C T C A T A C C C T A - C T G T T G C C T C G G C G -
CAGGCCGGCCCCCCCGGGGGCCCCTCGAGACGAGGAGCAGGCCGGCCGGCGGCCTAGTTAACTCTTGTTTT
TACACTGAAACTCTGAGAATAAACATAAATGAATCAAAACTTTCAACAACGGATCTCTTGGTTCTGGCATC
GATGAAGAACGCAGCGAAATGCGATAAGTAATGTGAATTGCAGAATTCAGTGAATCATCGAATCTTTGAAC
GCACATTGCGCCCTCTGGTATTCCGGAGGGCATGCCTGTTCGAGCGTCATTTCAACCCTCAAGCCTGGCTTG
GTGATGGGGCACTGCCTGTAAAAGGGCAGGCCCTGAAATTCAGTGGCGAGCTCGCCAGGACCCCGAGCGC
AGTAGT---TAAACCCTCGC-TCTGGAAGGCCCTGGCGGTGCCCT-GCCGTTAAA-CCCCCAA-
CTTCTGAAAATTTGACCTCGGATCAGGTAGGAATACCCGCTGAACTTAAGCATAGAGAAGGAAGGTTAGTA
AACATCACAATCACAGCACATGCTATCTTGCCCTCCCACCTCGAATCGAAACATCGGCGCGCGGCGGCCTG
CGCAGCTCCGTGAGACACGGGGGCGCATTTTCACCCCACGTCCTGGATTTTCCATTTTCAGTGCGGGTGCGG
GGTGCGCTTATCAGGGAGCTTATCTCCCACTCCCAAAACCCTGCTGCACCACT----

CCTTGCCACTACCACCACCA------TCACCATTCA-TCCTACTGCCATCTTCGAACACCA------

CAGAAAGCGCTGGAAATGATGCTAACAATCCAACTATATAGCCGCCGAGCTTGGCAAGGGTT-----------------

---AACCAAATCGGTGCTGCTTTCTGGTGCGTCCC-------

AGCTCCAAACTCACCACCGCGACGCTCGACGCGCGACAACACGGCCTCGCGGCATCGTTACTGACCTCGAC

T C T T -

AGGCAAACCATCTCTGGCGAGCACGGCCTCGACAGCAATGGCGTGTATGCACCTCCTATTCCCTGCACACA

GATCTCGCCCTCTCCTCCGGCTTGGCACTGACAGTTGCACAGTTACAACGGCACTTCCGAGCTCCAGCTCGA

GCGCATGAACGTCTACTTCAACGAGGTATGTCAACAACCATATTGTCCCATCCGACCGTTTCAGCACGGTTT

CCTATCGTCGTCCGGACCTCGCTGACGCGTTATCGCCCAGGCCTCCGGCAACAAGTATGTTCCTCGTGCGGT

CCTCGTCGATCTCGAGCCCGGTACCATGGACGCCGT

>KY203726.1 Diaporthe sp. YQ-2017a strain CFCC 51632

CCCTTTGTGAACTTATACCTTA-CTGTTGCCTCGGCG-CTAGCTGGTCCTT--CGGGGCCCCTT--

GGTGTTGAGACAGCCCGCCGGCGGCCAACCTAACGCTTGTTTTTACACTGAAACTCTGAGATAAAACATAA

ATGAATCAAAACTTTCAACAACGGATCTCTTGGTTCTGGCATCGATGAAGAACGCAGCGAAATGCGATAAG

TAATGTGAATTGCAGAATTCAGTGAATCATCGAATCTTTGAACGCACATTGCGCCCTCTGGTATTCCGGAGG

GCATGCCTGTTCGAGCGTCATTTCAACCCTCAAGCCTGGCTTGGTGATGGGGCACTGCCTGTAAAAGGGCA

GGCCCTGAAATTCAGTGGCGAGCTCGCCAGGACCCCGAGCGCAGTAGT---TAAACCCTCGC-

TTTGGAAGGCCCTGGCGGTGCCCT-GCCGTTAAA-CCCCCAA-CTTCTGAAA--

-------GGAAGGTTAGTAAATATCACAGTCACGGAACATGCTACCTGGCCCTCCTACTGCACCTC-

AATCATCAGCCCGCAGCTGCTCGCGCGGCCTCGCCATGTCGGGGGGCGCATTTTCACCCCTCGCTTTGGATT

TTCCATTTTCAGTGCGAGTGCGGGGTGCGCTTATCAGGGGGCTTATCTCCTACAACCAAAACCCTGTTACAT

CACTCACCAATCCTTGTCACCACCACCAATACGCTCACCATCAA-CCCCATCG-CCTCTTTCAAT-ACA-

ACTCGTGAAACGCGTCGAATATTATGCTGACCCTCTATCTACACAGCCGCCGAGCTTGGCAA-----

T G C G T A A G T T C C T - C C T G T C A A C A C C A G -

CGGACCTTATCATCGCCACCTGTAGCTGACACGTTTCCCAGGGTAACCAAATCGGTGCTGCTTTCTGGTGCG

T T C C A G C T -

CGAGCTCCAAGTCCACCGCCGCGACGCTCGACACGCGACAATACGACCTCGAAACATCATTGCTGACCTCG

A C T T -

TTAGGCAAACCATCTCTGGCGAGCACGGCCTCGACAGCAATGGCGTGTATGCACCTCCTATTCCCTGCCCA

CTGATCTTGGCCTCTCTTCCGGCTTGGCACTGACAATCGCATAGTTACAACGGCACTTCTGAGCTCCAGCTC

GAGCGCATGAACGTCTACTTCAACGAGGTAAGTCAATAACCAGAACATTCATCCGACCATCTCCAACACGG

TTTACTGCCGTCGCCCGAAGTTCGCTAACGCGGTATCGCCCAGGCCTCCGGCAACAAGTATGTTCCTCGCGC

CGTCCTCGTCGATCTCGAGCCCGGTACCATGGACGCCGT

>MH121498.1 Diaporthe caryae strain CFCC 52563

CCCTTTGTGAACTTATACC-TA-

CTGTTGCCTCGGCGTCAGGCCGGCCCCTACTGGGGCCCCCCGAGACGGGGAGCA-

GCCCGCCGGCGGCCAACTAAACTCTTGTTTCTATAGTGAATCTCTGAGAAAAAACATAAATGAATCAAAAC

TTTCAACAACGGATCTCTTGGTTCTGGCATCGATGAAGAACGCAGCGAAATGCGATAAGTAATGTGAATTG

CAGAATTCAGTGAATCATCGAATCTTTGAACGCACATTGCGCCCCCTGGTATTCCGGGGGGCATGCCTGTTC

GAGCGTCATTTCAACCCTCAAGCCTGGCTTGGTGATGGGGCACTGCTTCGAGAGGAGCAGGCCCTGAAATT

CAGTGGCGAGCTCGCCAGGACCCCGAGCGTAGTAGT---TATA-TCTCGC-

TTTGGAAGGCCCTGGCGGTGCCCT-GCCGTTAAA-CCCCCAA-CTTCTGAAA--

- -

GAGAAGGAAGGTCAGTAAACATTACATCCGCATCATGAACCCCAACTCCGCCTGACGCACATCGTCACCAT

GGGCGCGCGACTTTTTGCACGGCTGCGTCACACCCGTCAAC-

CATTTTCGCCCCTCCCTCTGGATTTTTCATTTTCAGTGCGGGTGCGGGGTGTGCTTATCAGGCCGCTTATCTC

CCACACGCAAAACCCTGTGGCATCAATCACTCCTTG-

CACTGCCTGCTCCAACATCACAGGCATCAAACACCATCAACCTCT----------

ACTTGCGCAACGTTCGGACAATCATGCTGACTTACATCCCACACAGCCGCCGAGCTGGGTAAGGGTTTGCG

TAAGTTGCCTCCTGTCAACACCGC-CCGACCTTATCGCCACCCC--

GTAGCTGACACGTTTCCCAGGGTAACCAAATCGGTGCTGCTTTCTGGTGCGTACCAGCTC-

CAGCTCCGAGCCTGCCACCGCGACGCTCGACGCGCGACAAGACTAGCTCGCAACATCTTTACTGACCTCGT

CTCTTTAGGCAAACCATCTCTGGCGAGCACGGTCTCGACAGCAATGGCGTGTATGTACCTCCTATTCCCTGC

CCACCGATCTCGTCCTCTCCTC--

GCTTGGCACTGATGATCGCACAGTTACAACGGCACTTCCGAGCTCCAGCTCGAGCGCATGAATGTCTACTT

CAACGAGGTAAGCCTATGGCCACGTCTTCAATCCAAA--

CGTCTGGCATGGTTTACTGCCGCCGCCAGGGCCTTGCTAATGCGCTCTCCCCCAGGCCTCCGGCAACAAGTA

TGTGCCTCGCGCCGTCCTCGTCGATCTCGAGCCCGGTACCATGGACGCCG-

>MK626952.1 Diaporthe caryae strain PSCG 520

CCCTTTGTGAACTTATACC-TA-

CTGTTGCCTCGGCGTCAGGCCGGCCCCTACTGGGGCCCCCCGAGACGGGGAGCA-

GCCCGCCGGCGGCCAACCAAACTCTTGTTTCTATAGTGAATCTCTGAGAAAAAACATAAATGAATCAAAAC

TTTCAACAACGGATCTCTTGGTTCTGGCATCGATGAAGAACGCAGCGAAATGCGATAAGTAATGTGAATTG

CAGAATTCAGTGAATCATCGAATCTTTGAACGCACATTGCGCCCCCTGGTATTCCGGGGGGCATGCCTGTTC

GAGCGTCATTTCAACCCTCAAGCCTGGCTTGGTGATGGGGCACTGCTTCGAGAGGAGCAGGCCCTGAAATT

CAGTGGCGAGCTCGCCAGGACCCCGAGCGTAGTAGT---TATA-TCTCGC-

TCTGGAAGGCCCTGGCGGTGCCCT-GCCGTTAAA-CCCCCAA-

CTTCTGAAAATTTGACCTCGGATCAGGTAGGAATACCCGCTGAACTTAAGCATA---------------------------

CATCATGAACCCCAGAACTCCGCCTGACGCACCGTCACCATGGGCGCGCGACTTTTTGCACGGCTGCGTCA

C A C C C G T C A A C -

CATTTTCGCCCCTCCCTCTGGATTTTTCATTTTCAGTGCGGGTGCGGGGTGTGCTTATCAGGCCGCTTATCTC

CCACACGCAAAACCCTGTGGCATCAATCACTTCCTTGCACTGCCTGCTCCAACATCACAGGCATCAAACAC

C A T C A A C C T C T A C T T G C G C A A - - - - - -

CAAACTGTTCGGACAATCATGCTGACTTACATCCCACACAGCCGCCGAGCTGGGTAAGGGTT-----------------

--GCTGGTGATGTCAGCTCGA-GCCTGCC-

CCGCGACGCTCGACGCGCGACAAGACTAGCTCGCAACATCTTTACTGACCTCGTCTCTTTAGGCAAACCAT

CTCTGGCGAGCACGGTCTCGACAGCAATGGCGTGTATGTACCTCCTATTCCCTGCCCACCGATCTCGTCCTC

T C C T C C G -

CTTGGCACTGATGATCGCACAGTTACAACGGCACTTCCGAGCTCCAGCTCGAGCGCATGAATGTCTACTTC

A A C G A G G T A A G C C T A T G G C C A C G T C T T C A A T C C A A A C G T C T -

CGGCATGGTTTACTGCCGCCGCCAGGGCCTTGCTAATGCGCTCTCGCCCAGGCCTCCGGCAACAAGTACGT

GCCTCGCGCCGTCCTCGTCGATCTCGAGCCCGGTACCATGGACGCCGT

>KX024653.1 Diaporthe betulicola strain CFCC 51128

C C C T T T G T G A A C T T A T A C C T T A - C T G T T G C C T C G G C G -

CAGGCCGGCCCCCCCGGGGGCCCCTCGAGACGAGGAGCAGGCCCGCCGGCGGCCAAGTTAACTCTTGTTTT

TACACTGAAACTCTGAGACAAAACATAAATGAATCAAAACTTTCAACAACGGATCTCTTGGTTCTGGCATC

GATGAAGAACGCAGCGAAATGCGATAAGTAATGTGAATTGCAGAATTCAGTGAATCATCGAATCTTTGAAC

GCACATTGCGCCCTCTGGTATTCCGGAGGGCATGCCTGTTCGAGCGTCATTTCAACCCTCAAGCCCGGCTTG

GTGATGGGGCACTGCTTCTCGAGAAGCAGGCCCTGAAACTCAGTGGCGAGCTCGCCAGGACCCCGAGCGC

AGTAGT---TAAACCCTCGC-TCTGGAAGGCCCTGGCGGTGCCCT-GCCGTTAAA-CCCCCAA-

C T T C T G A A A A T T T -

GAGAAGGAAGGTTAGTAAACATCACAATCACAGCACATGCTGTCTTGCCCTCACACC-----

T C G A G A C A T C G G C G C G C G G C G G C C T G C G C G G C T C C G C C A C G C - -

GGGGGCGCATTTTCACCCCTCGTTCTGGATTTTCCATTTTCAGTGCGGGTGCGGGGTGCGCTTATCAGGGAG

CTTATCTCCCGCTCCCAAAACCCTGCTGCACCAC-TCCCCACTACCACCACCA---CCA------TCACCATCAA-

T C C T A C T G C C A C C C T T C A A C A C C A C A C A A A - - - - - -

GCGCTGGAAATGATGCTAACAATCCAATTATACAGCCGCCGAGCTTGGCAAGGGTTTGCGTAAGTTGCT-

C C T G T C A A C A C C A C - C G G A C C C - -

TTATCGCCACCTGTAGCTGACACGTTTCCCAGGGTAACCAAATCGGTGCTGCTTTCTGGTGCGTCCC------

AGCTCCAAACTCACCACCGCGACGCTCGACGCGCGACAACACGACCTCGCGGCATCGTTACTGACCCCGAC

T C -

TTAGGCAAACCATCTCTGGCGAGCACGGCCTCGACAGCAATGGCGTGTATGCACCTCCTATTCCCTGCACA
CAGATCTCGCCCTGTCCTCCGGCTTGGCATTGACAATCGCACAGTTACAACGGCACTTCCGAGCTCCAGCTC
G A G C G C A T G A G C G T C T A C T T C A A C G A G G T A T G T C T A C A A C C A T -
TGTCCCATTCGACCGTCTTCAACACGGTGTCCTACCGTCGTCCGGACCTCGCTAACGCGTTATTGCCCAGGC
CTCCGGCAACAAGTATGTTCCTCGCGCGGTCCTCGTCGATCTTGAGCCCGGTACCATGGACGCCGT

>MH121495.1 Diaporthe betulina strain CFCC 52560

CCCTTTGTGAACTTATACCTTA-CTGTTGCCTCGGCG-CATGCCGGCCCCCCTGGGGGCCCCTT-----
GAGGAGCAGGCACGCCGGCGGCCAACCTAACTCTTGTTTTTACACTGAAACTCTGAGATAAAACATAAATG
AATCAAAACTTTCAACAACGGATCTCTTGGTTCTGGCATCGATGAAGAACGCAGCGAAATGCGATAAGTAA
TGTGAATTGCAGAATTCAGTGAATCATCGAATCTTTGAACGCACATTGCGCCCTCTGGTATTCCGGAGGGC
ATGCCTGTTCGAGCGTCATTTCAACCCTCAAGCCTGGCTTGGTGATGGGGCACTGCTTTTAAA--
AGCAGGCCCTGAAATTCAGTGGCGAGCTCGCCAGGACCCCGAGCGCAGTAGT---TAAACCCTCGC-
TTTGGAAGGCCCTGGCGGTGCCCT-GCCGTTAAA-CCCCCAA-CTTCTGAAA---
- -
GAGAAGGAAGGTTAGTAAATACCACAGTCACGGAACATGCCACCCTGCCCTCCTACTGCACCTCAAGTCAT
CAGCCCGCAGCTGCTCGCGCGGCTTCGTCATGTCTGGGGGCGCATTTTCACCCCTCGCTTTGGATTTTCCAT
TTTCAGTGCGGGTGCGGGGTGCGCTTATCAGGGGGCTTATCTCCTACACCCAAAACCCTGTTGCATCACTCA
CTCCTCGCCACCACCACCACCAATACGCTCACCATCAA--TCCATCG-CCCTTTTCAATA---------
CAACCCACGTCGAACATTATGCTGACCCTCTATCTACACAGCCGCCGAGCTTGGTAAGGGTTTGCGTAAGTT
G C T - C C T G T C A A C A C C A G -
CGGACCTTATCATCGCCACCTGTAGCTGACACGTTTCCCAGGGTAACCAAATCGGTGCTGCTTTCTGGTGCG
T T C C A G C T -
CGAGCTCCAAGTCCACCGCCGCGACGCTCGACACGCGACAATACGACCTCGAAGCATCGTTGCTGACCTCG
A C T T -
TTAGGCAAACCATCTCTGGCGAGCACGGCCTCGACAGCAATGGCGTGTATGCACCTCCTATTCCCTGCCCA
CTGATCTTGGCCTCTCTTCCGGCTTGGCACTGACAATCGCACAGTTACAACGGCACTTCTGAGCTCCAGCTC
GAGCGCATGAACGTCTACTTCAACGAGGTAAGTCAACAACCACATCATCCATCCGACCATCTCCAACACGG
TTTACTGCCGTCGCCCGGACCTCGCTAACGCGTCATCGCCCAGGCCTCCGGCAACAAGTATGTTCCTCGCGC
CGTCCTCGTCGATCTCGAGCCCGGTACCATGGACGCCG-

>MG806115.1 Diaporthe collariana isolate MFLUCC 17-2636

CCCTTTGTGAACTTATACCTTA-CTGTTGCCTCGGCG-CTAGCTGGTCCTT--CGGGGCCCCTT-----
GAGGAGCAGGCACGCCGGCGGCCAACCCAACTCTTGTTTTTACACTGAGACTCTGAGACAAAACATAAATG

AATCAAAACTTTCAACAACGGATCTCTTGGTTCTGGCATCGATGAAGAACGCAGCGAAATGCGATAAGTAA
TGTGAATTGCAGAATTCAGTGAATCATCGAATCTTTGAACGCACATTGCGCCCTCTGGTATTCCGGAGGGC
ATGCCTGTTCGAGCGTCATTTCAACCCTCAAGCCTGGCTTGGTGATGGGGCACTGCCTGTAAAAGGGCAGG
CCCTGAAATTCAGTGGCGAGCTCGCCAGGACCCCGAGCGCAGTAGT---TAAACCCTCGC-
TTTGGAAGGCCCTGGCGGTGCCCT-GCCGTTAAA-CCCCCAA-CTTTGAAAA--TTGACCTCG--------------------
----
GAGAAGGAAGGTTAGTAAATACCACAATCACGGAACATCCTACCCTGCCCTGATACTGCACCTCAAATCAT
CAGCCCGAAGCTGCTCGCGCGGCTTCGTCATATCGGGGGGCGCATTTTCACCCCTCGCTTTGGATTTTCCAT
TTTCAGTGCGGGTGCGGGGTGCGCTTATCAGGGGGCTTATCTCCTACTCCCAAAACCCTGTTGCCTCACTCG
CCTACTACCACCACCACTACCAATACGCTCACCATTAA-TCCCATCG-TCTCTACCAAC-ATA-
ACTCGTGAAACGCGTTGAATACTATGCTGACACTCTATCTACACAGCCGCCGAGCTTGGTAAGGGTT--------
--ACGGTGCTTTCTGGTGCGTCCCAGCT-
CGAGCTCCAAGTCCACCGCCGCGACGCTCGACACGCGACAATACGACCTCGAAGCATCGTTACTGACCTCG
ACTC-
TCAGGCAAACCATCTCTGGCGAGCACGGCCTCGACAGCAATGGCGTGTATGCACCTCCTATTCCCTGCCCA
CTGATCTTGGCCTCTCTTCCGGCTTGGCACTGACAATTGCACAGTTACAACGGCACTTCTGAGCTGCAGCTC
GAGCGCATGAACGTCTACTTCAACGAGGTAAGTCAACAACCACATCATCCATCCGACCATCTCCAACACGG
TTTACTGCCGTCGCCCGGACCTCGCTAACGTGTTATCGCCCAGGCCTCCGGCAACAAGTATGTTCCTCGCGC
CGTCCTCGTCGATCTCGAGCCCGGTACCATGGACGCCGT

>KJ490624.1 Diaporthe discoidispora isolate ZJUD89

CCCTTTGTGAACTTATACCTTA-CTGTTGCCTCGGCG-
CAGGCCGGCCTCTGCTGAGGCCCCCGAGACGGGGAGCA-
GCCCGCCGGCGGCCAAGTTAACTCTTGTTTCTACCATGAATCTCTGAGAAAAAACATAAATGAATCAAAAC
TTTCAACAACGGATCTCTTGGTTCTGGCATCGATGAAGAACGCAGCGAAATGCGATAAGTAATGTGAATTG
CAGAATTCAGTGAATCATCGAATCTTTGAACGCACATTGCGCCCTCTGGTATTCCGGAGGGCATGCCTGTTC
GAGCGTCATTTCAACCCTCAAGCCTGGCTTGGTGTTGGGGCACTGCTTCGAGAGAAGCAGGCCCTGAAATC
TAGTGGCGAGCTCGCTAGGACCCCGAGCGTAGTAGT---TATA-TCTCGT-
TCTGGAAGGCCCTGGCGGTGCCCT-GCCGTTAAA-CCCCCAA-CTTCTGAAAATT--------------------------------
-----GAGAAGGAAGGTTAGTAAACATCA-AGCCGCAGCATACAATCTCTTGCCCTCCTATGCACTC-
AGAATCATCGGCGCGCTACATTCTGCGCGGCTTCGTAACATCCTAAAG-
GCATTTTCACCCCTCGCTCTGGATTTTCCATTTTCAGTGCGGGTGCGGGGTGCGCTTATCAGGCGGCTTATCT
CTTACATCCAAAACCCTCTTGCATCAACCACTCCATCCCGTTGCTACTACCAACAATCACACAATCAT-
CTTCATAA-----------------
GCAGAAGAACCGTGCTGGAACCTAAGCTGACCTTTTTTCTACACAGCCGCCGAGCTTGGTAAGGGT----------

----

AGCTCGCAACATCGTTACTGACCTAGACTCTTTAGGCAAACCATCTCTGGCGAGCACGGTCTCGACAGCAA

T G G C G T G T A T G C A A C T C C T A T T C C C T G C C C A C T G A T A T C G T C C T C T C C T C - -

GCCTGGCACTGACAATTGCACAGTTACAACGGCACTTCCGAGCTCCAGCTCGAGCGCATGAACGTCTACTT

CAACGAGGTAAGTCAATAGCCACGTCGTCAATACAACCATCTACTGCATGGTCTCCTGCCGCCGCCGAGGT

CTTGCTAACGCGTTACCGCCCAGGCCTCCGGCAACAAGTATGTGCCTCGCGCCGTCCTCGTCGATCTCGAGC

CCGGTACCATGGACGCCGT

>KC343065.1 Diaporthe endophytica strain CBS 133811

C C C T T T G T G A A C T T A T A C C - T A - T T G T T G C C T C G G C G -

T A G G C C G G C C T C T A C T G A G G C C C C C T G A A A C A G G G A G C A -

GCCCGCCGGCGGCCAACCAAACTCTTGTTTCTACAGTGAATCTCTGAGAAAAAACATAAATGAATCAAAAC

TTTCAACAACGGATCTCTTGGTTCTGGCATCGATGAAGAACGCAGCGAAATGCGATAAGTAATGTGAATTG

CAGAATTCAGTGAATCATCGAATCTTTGAACGCACATTGCGCCCTCTGGTATTCCGGAGGGCATGCCTGTTC

GAGCGTCATTTCAACCCTCAAGCCTGGCTTGGTGATGGGGCACTGCCTGTAAAAGGGCAGGCCCTGAAATC

T A G T G G C G A G C T C G C C A G G A C C C C G A G C G T A G T A G T - - - T A T A - T C T C G C -

T C T G G A A G G C C C T G G C G G T G C C C T - G C C G T T A A A - C C C C C A A -

CTTCTGAAAATTTGACCTCGGATCAGGTAGGAATACCCGCTGAACTTAAGCATAGAGAAGGAAGGTTAGTA

AACATCG-AACGGCACCTTGACCTCC-ACATCACCCCATACGCCCAGAACTCGTTGGCGCGC-

G C T G T C T A C G A G G C T T C G T C A C A C T C A A - - - -

GCATTTTCACCCCTCCCTCTGGATTTTCCATTTTCAGTGCGGGTGCGGGGTGCGCTTATCAGGCCGCTTATCT

TTCACAC-CAAAACCCTGTCGCACC------TACTCACCAGTGACG-CACCAACAGCATCACCTTCAT-

T C C C A T C T - T T T G T T T G A G - - - - - - - - - - - G G A G C T C T C T C G A A A A T G A T G C T G A C T - -

CTTTCTGAACAGCCGCCGAGCTGGGTAAGGGTTTGCGTAAGTTGCCTCCTGTCAACACCGC-

C A G G G C T T A T C G C C A C C C - - -

GTAGCTGACACGTTTCCCAGGGTAACCAAATCGGTGCTGCTTTCTGGTGCGTCGCAGCTCCCAGCTCCAAAC

CTACCACCGCGGCCCTCGACGCGCGACAAGGCGAGCTCGAAGCATCGATACTGACCTCGTTTCTCTAGGCA

AACCATCTCTGGCGAGCACGGTCTCGACAGCAATGGCGTGTATGCACCTCCTATTCCCTGCCCACTGACCTG

G T C C T C T C C T C - -

GCTTGGCACTGATGATCGCACAGTTACAACGGCACTTCCGAGCTCCAGCTCGAGCGCATGAACGTCTACTT

C A A C G A G G T A T G T C A A C G G C C A C G T C G T C A A T T C A A A -

AGCTCTGGCATGGTCAACTGCCGCCGCCAAGCCCTTGCTAACGCGTTTCCCCCAGGCTTCCGGCAACAAGT

ATGTGCCCCGCGCCGTCCTCGTCGATCTCGAGCCCGGTACCATGGACGCCGT

>KJ210529.1 Diaporthe eres isolate AR5193

CCCTTTGTGAACTTATACCTTA-CTGTTGCCTCGGCG-CTAGCTGGTCCCT--CGGGGCCCCTT--
GGTGTTGAGACAGCCCGTCGGCGGCCAACCTAACTCTTGTTTTACACTGAAACTCTGAGACAAAACATAA
ATGAATCAAAACTTTCAACAACGGATCTCTTGGTTCTGGCATCGATGAAGAACGCAGCGAAATGCGATAAG
TAATGTGAATTGCAGAATTCAGTGAATCATCGAATCTTTGAACGCACATTGCGCCCTCTGGTATTCCGGAGG
GCATGCCTGTTCGAGCGTCATTTCAACCCTCAAGCCTGGCTTGGTGATGGGGCACTGCTTCTCAAGAAGCA
GGCCCTGAAATTCAGTGGCGAGCTCGCCAGGACCCCGAGCGCAGTAGT---TAAACCCTCGC-
T C T G G A A G G C C C T G G C G G T G C C C T - G C C G T T A A A - C C C C C A A -
C T T C T G A A A A T T T G A C C T C G G A T C A G G T A G G A A T A C C C G - - - - - - - - - - - - - -
GAGAAGGAAGGTTAGTAAATATCACAGTCACGGAACATGCTACCTGGCCCTCCTACTGCACCTC-
AATCATCAGCCCGCAGCTGCTCGCGCGGCCTCGCCATGTCGGGGGGCGCATTTTCACCCCTCGCTTTGGATT
TTCAATTTTCAGTGCGAGTGCGGGGTGCGCTTATCAGGGGGCTTATCTCCTACAACCAAAACCCTGTTACAT
CACTCACCAATCCTTGTCACCACCACCAATACGCTCACCATCAA-CCCCATCG-CCTCTTTCAAT-ACA-
ACTCGTGAAACGCGTCGAATATTATGCTGACCCTCTATCTACACAGCCGCCGAGCTTGGTAAGGGTT---------
---GGTGCTGCTTTCTGGTGCGTTCCAGC--
CGAGCTCCAAGTCCACCGCCGCGACGCTTGACACGCGACAATACGACCTCGAAGCATCGTTGCTGACCTCG
A C T T -
TTAGGCAAACCATCTCTGGCGAGCACGGCCTCGACAGCAATGGCGTGTATGCACCTCCTATGCCCTGTCCA
CTGATCTTGACCTCTCTTCCGGCTTGGCACTGACAATCGCACAGTTACAACGGCACTTCTGAGCTCCAGCTC
GAGCGCATGAACGTCTACTTCAACGAGGCAAGTCAATAACCACAACATTCATCCGACCATCTCCAACACGG
TTTACTGCCGTCGCCCGAAGTTCGCTAACGCGTTATCGCCCAGGCCTCCGGCAACAAGTATGTTCCTCGCGC
CGTCCTCGTCGATCTCGAGCCCGGTACCATGGACGCCGT

>KC343112.1 Diaporthe ganjae strain CBS 180.91

C C C T T T G T G A A C C T A T A C C - C A - C T G T T G C C T C G G C G - C A G G C C G G T C T G T -
C T C A G A C C C C C T G A G A C A G G G A G C A - G C C C G C C G G C G G C C G A C C A A A C T C -
CGTTTCTATAGTGAATCTCTGAGTAAAAACATAAATGAATCAAAACTTTCAACAACGGATCTCTTGGTTCTG
GCATCGATGAAGAACGCAGCGAAATGCGATAAGTAATGTGAATTGCAGAATTCAGTGAATCATCGAATCTT
TGAACGCACATTGCGCCCTCTGGTATTCCGGAGGGCATGCCTGTTCGAGCGTCATTTCAACCCTCAAGCCTG
GCTTGGTGATGGGGCAGTGCCTTGGACAAGGCACGCCCTGAAATTCAGTGGCGAGCTCGCCAGGACCCCGA
GCGTAGTAGT---TACA-TCTCGC-TCCGGAAGGCCCTGGCGGTGCCCT-GCCGTTAAA-CCCCCAA-
CTTCTGAAAATTTGACCTCGGATCAGGTAGGAATACCCGCTGAACTTAAGCATA-------------------A----
ACTCACAACATCAACCCCAAGTCATCCCAGTGCACATCGCGATCATCGGCGCGCGACCGTCTGCGCCGCTT
C G T C A C T C - - -
GTCACGCATTTTCACCCCTCGTCCTGGATTTTCCACTTTCAGTGCGGGTGCGGGGTGTGCTTATCAGGCCGC

TTATCGCTTGCGTTCAAAACCCTGCTGCACCAACCACTCTTCGCCGCTGCCCACATTAACGCCAACACCATC

A A - T C T C A T C A A A C C C C A C C C T C T G A C A A A A C A A A C C G - - - -

GAAAACCATGCTGACTCGCTTTCCATACAGCCGCCGAGCTGGGTAAGGGTTTGCGTAAGTTGCTCCCTGTC

A A C A C C G C - C C G A C C T T A T C G C C A C C C - - -

GTAGCTGACACGTTTCCCAGGGTAACCAAATCGGTGCTGCTTTCTGGTGCGTCCCAGCTCCCAGCTCCAGGC

CTACCGCCGCGATGCTCGACGCGCGACAAGACCAGCTCGCAACATTGTTACTGACCTAGGCTCTGTAGGCA

AACCATCTCTGGCGAGCACGGTCTCGACAGCAATGGCGTGTATGCACCTCCTATTCCCTGCCCACTGATCTC

G T C C T G T C C T C - -

GCTTGGCACTGATGATTGCACAGTTACAACGGCACTTCCGAGCTCCAGCTCGAGCGCATGAACGTCTACTT

CAACGAGGTATGTCAATGGCCACGTCGTCAATCCAAACATCTACTGCGCGGCGTGCTGCCGTAGCCAAAGC

CTTGCTAACGCGTTGTCGCCCAGGCTTCCGGCAACAAGTATGTGCCCCGCGCTGTCCTCGTCGATCTCGAGC

CCGGTACCATGGACGCCGT

>MK626955.1 Diaporthe ganjae strain PSCG 489

C C C T T T G T G A A C T T A T A C C - C A - C T G T T G C C T C G G C G - C A G G C C G G T C T G T -

C T C A G A C C C C C T G A G A C A G G G A G C A - G C C C G C C G G C G G C C A A C C A A A C T C -

TGTTTCTATAGTGAATCTCTGAGAAAAAACATAAATGAATCAAAACTTTCAACAACGGATCTCTTGGTTCTG

GCATCGATGAAGAACGCAGCGAAATGCGATAAGTAATGTGAATTGCAGAATTCAGTGAATCATCGAATCTT

TGAACGCACATTGCGCCCTCTGGTATTCCGGAGGGCATGCCTGTTCGAGCGTCATTTCAACCCTCAAGCCTG

GCTTGGTGATGGGGCAGTGCCTTGGACAAGGCACGCCCTGAAATTCAGTGGCGAGCTCGCCAGGACCCCGA

GCGTAGTAGT---TACA-TCTCGC-TCTGGAAGGCCCTGGCGGTGCCCT-GCCGTTAAA-CCCCCAA-

CTTCTGAAAATTTGACCTCGGATCAGGTAGGAATACCCGCTGAACTTAAGCATA--------------------CAC-------

-ATCAACCCCAAGTCATCCCAGTGCACATCGCGATCATCGGCGCGCGACCGTCTGCGCCGCTTCGTCACTC---

GTCACGCATTTTCACCCCTCGTTCTGGATTTTCCACTTTCAGTGCGGGTGCGGGGTGTGCTTATCAGGCCGC

TTATCGCTTGCGTTCAAAACCCTGTGGCATCAACCACTCTTTGCCGCTGCCCACATTAACGCCAACGCCATC

A A - T C T C A T C A C C C C A C C C C C - - - - - - -

TCTGACAAGACCTGAGGAAAACAATGCTGACTCGCTTTCCATACAGCCGCCGAGCTGGGTAAGGGTT-------

- -

GGTGCTGCTTTCTGGTGCGTCCCAGCTCCCAGCTCCAGGCCTACCGCCGCGATGCTCGACGCGCGACAAGA

CCAGCTCGCAACATTGTTACTGACCTCGGCTCTGTAGGCAAACCATCTCTGGCGAGCACGGCCTCGACAGC

A A T G G C G T G T A T G C A C C T C C T A T T C C C T G C C C A C T G A T C C C A T C C T G T C C T C - -

GCTTGGCACTGATGATTGCACAGTTACAACGGCACTTCCGAGCTCCAGCTCGAGCGCATGAACGTCTACTT

CAACGAGGTATGTCAATGGCCACGTCGTCAATCCAGACATCTACTGCGCCGCGTGCTGCCGTAGCCAAAGC

CTTGCTAACGCGTTGTCGCCCAGGCTTCCGGCAACAAGTATGTGCCCCGCGCTGTCCTCGTCGATCTCGAGC

CCGGTACCATGGACGCCGT

>KJ210538.1 Diaporthe nitschkei isolate AR5211

CCCTTTGTGAACTTATACCTTA-CTGTTGCCTCGGCG-CATGCCGGCCCCCCTGGGGGCCCCTT-----
GAGGAGCAGGCACGCCGGCGGCCAACCTAACTCTTGTTTTTACACTGAAACTCTGAGATAAAACATAAATG
AATCAAAACTTTCAACAACGGATCTCTTGGTTCTGGCATCGATGAAGAACGCAGCGAAATGCGATAAGTAA
TGTGAATTGCAGAATTCAGTGAATCATCGAATCTTTGAACGCACATTGCGCCCTCTGGTATTCCGGAGGGC
ATGCCTGTTCGAGCGTCATTTCAACCCTCAAGCCTGGCTTGGTGATGGGGCACTGCTTCTCAAGAAGCAGG
CCCTGAAATCCAGTGGCGAGCTCGCCAGGACCCCGAGCGCAGTAGT---TAAACCCTCGC-
TTTGGAAGGCCCTGGCGGTGCCCT-GCCGTTAAA-CCCCCAA-
CTTCTGAAAATTTGACCTCGGATCAGGTAGGAATACCCG---------------
GAGAAGGAAGGTTAGTAAATATCACAATCACGGAACATGCTACCTGGCCCTCCTACTGTACCTCAAATCAT
CAGCCCGCAGCTGCTCGCGCGGCCTCGCCATATCGGGGGGCGCATTTTCACCCCTCGCTTTGGATTTTCCAT
TTTCAGTGCGAGTGCGGGGTGCGCTTATCAGGGGGCTTATCTCCTACAACCAAAACCCTGTTACATCACTCA
CCAATCCTTGTCACCACCACCAATTCGCTCAGCATCAA-CCCCATCG-CCTCTGTCAAT-ACA-
ACTCGTGAAACGCGTCGAATATTATGCTGACCCTCTATCTACACAGCCGCCGAGCTTGGTAAGGGTT---------
--GGTGCTGCTTTCTGGTGCGTTCCAGC--
CGAGCTCCAAGTCCACCGCCGTGACGCTCGACACGCGACAATACGACCTCGAAGCATCGTTGCTGACCTCG
ACTT-
TTAGGCAAACCATCTCTGGCGAGCACGGCCTCGACAGCAATGGCGTGTATGCACCTCCTATTCCCTGCCCA
CTGATCTTGGCCTCTCTTCCGGCTTGGCACTGACAATCGCATAGTTACAACGGCACTTCTGAGCTCCAGCTC
GAGCGCATGAACGTCTACTTCAACGAGGTAAGTCAACAACCACATCATCCATCCGACCATCTCCAACACGG
TTTACTGCCGTCGCCCGGACCTCGCTAACGTGTTATCGCCCAGGCCTCCGGCAACAAGTATGTTCCTCGCGC
CGTCCTCGTCGATCTCGAGCCCGGTACCATGGACGCCGT

>MK335809.1 Diaporthe hubeiensis isolate JZB320123

CCCTTTGTG-ACTTATACCATA-CTGTTGCCTCGGCG-
CTGGCCGGCCTCCACCGAGGCCCCCTGAGACAGGGAGCA-
GCCCGCCGGCGGCCAAACAAACTCTTGTTTCT-TAGTGAATCTCTGAGAAAAAACAT-
AATGAATCAAAACTTTCAACAACGGATCTCTTGGTTCTGGCATCGATGAAGAACGCAGCGAAATGCGATAA
GTAATGTGAATTGCAGAATTCAGTGAATCATCGAATCTTTGAACGCACATTGCGCCCTCTGGTATTCCGGAG
GGCATGCCTGTTCGAGCGTCATTTCAACCCTCAAGCCTGGCTTGGTGTTGGGGCACCGCCTTTAAAAGGGC
GGGCCCTGAAATCTAGTGGCGAGCTCGCCAGGACCCCGAGCGTAGTAGT---TATA-TCTCGT-
TCTGGAAGGCCCTGGCGGTGCCCT-GCCGTTAAA-CCCCCAA-
CTTCTGAAATTTTGACCTCGGATCAGGTAGGAATACCCGCTGAACTTAAGCATAGAGAAGGAAGGTT--------

- - -
AGTTACGGCATACACTCCCTTGTACCCAGTTGCACATCAGGATTACTGGCGCGCTGCCGTTTGCGCGGCTTC
G T C A C A C C C G C C A G -
GCATTTTCACCCCTCCCTCTGGATTTTCCATTTTCAGTGCGGGTGCGGGGTGCGCTTATCAGGCGGCTTATCT
CTGACGCTACAAACCCTGCTGCATCAACCATTCCTTGTCGCTGCCACCACCA---CCATCACCGTCAA-
A T T C A T C G - - - - - - - - - - - C -
ACGGTCAAATTGATCACGTTGGAGATGAAGCTGACTTTCCATCTCTATAGCCGCCGAGCTCGGTAAGGGTT-
---GGTGCTGCTTTCTGGTGCGTTCCCGCTC--
AGCTCAGAGTCGACCACCGCGACAATCGACGCGCGACAACAGTAGCTCGTGGCATTGATACTGACATCGGC
T C C T -
AGGCAAACCATCTCTGGCGAGCACGGCCTCGACAGCAATGGCGTGTATGCACCTCCTATTCCCTGCCTACT
GGTCTCGTCCTCTCTCCTACCTTGGCACTGATAATGGCACAGCTACAACGGCACTTCTGAGCTCCAGCTCGA
GCGCATGAACGTCTACTTCAACGAGGTGAGCCAAAGCCCACGTTGTCAATCCGGACACCTGCAGCACAATC
CCCTGCCACCGCCAAGGCCTAGCTAACGCGTTATCGTCCAGGCCTCCGGCAACAAGTATGTGCCTCGCGCC
GTCCTCGTCGATCTCGAGCCCGGTACCATGGATGCCGT

>KF576267.1 Diaporthe longispora (nom. illeg.) strain CGMCC 3.17089

CCCTTTGTGAACTTATACC-TA-CTGTTGCCTCGGCG-CTAGCTGGTCCTT--CGGGGCCCCTC--
GGTGTTGAGACAGCCCGCCGGCGGCCAACCCAACTCTTGTTTTACACTGAAACTCTGAGATAAAACATAA
ATGAATCAAAACTTTCAACAACGGATCTCTTGGTTCTGGCATCGATGAAGAACGCAGCGAAATGCGATAAG
TAATGTGAATTGCAGAATTCAGTGAATCATCGAATCTTTGAACGCACATTGCGCCCTCTGGTATTCCGGAGG
GCATGCCTGTTCGAGCGTCATTTCAACCCTCAAGCCTGGCTTGGTGATGGGGCACTGCCTGTAAAAGGGCA
GGCCCTGAAATTCAGTGGCGAGCTCGCCAGGACCCCGAGCGCAGTAGT---TAAACCCTCGC-
TCTGGAAGGCCCTGGCGGTGCCCT-GCCGTTAAA-CCCCCAA-CTTCTGAAAATTT-------------------------------
--------------------GTAAATATCACAGTCACGGAACATGCTACCTGGCCCTCCTACTGCACCTC-
AATCATCAGCCCGCAGCTGCTCGCGCGGCCTCGCCATGTCGGGGGGCGCATTTTCACCCCTCGCTTTGGATT
TTCCATTTTCAGTGCGAGTGCGGGGTGCGCTTATCAGGGGGCTTATCTCCTACAACCAAAACCCTGTTACAT
CACTCACCAATCCTTGTCACCACCACCAATACGCTCACCATCAA-CCCCATCG-ACTCTTTCAAT-ACA-
ACTCGTGAAACGTGTCGAATATTATGCTGACCCTCTATCTACACAGCCGCCGAGCTTGGTAAGGGTT---------
---GGTGCTGCTTTCTGGTGCGTTCCAGC--
CGAGCTCCAAGTCCACCGCCGCGACGCTCGACACGCGACAATACGACCTCGAAGCATCGTTGCTGACCTCG
A C T T -
TTAGGCAAACCATCTCTGGCGAGCACGGCCTCGACAGCAATGGCGTGTATGCACCTCCTATTCCCTGCCCA
CTGATCTTGGCCTCTCTTTCGGCTTGGCACTGACAATCGCACAGTTACAACGGCACTTCTGAGCTCCAGCTC
GAGCGCATGAACGTCTACTTCAACGAGGTAAGTCAATAACCACAACATTCATCCGACCATCTCCAACACGG

TTTACTGCCGTCGCCCGAAGTTCGCTAACGCGTTATCGCCCAGGCCTCCGGCAACAAGTATGTTCCTCGCGC
CGTCCTCGTCGATCTCGAGCCCGGTACCATGGACGCCGT

>KJ590728.1 Diaporthe longicolla isolate FAU599

C C C T T T G T G A A C T T A T A C C - T A - C T G T T G C C T C G G C G -
C A G G C C G G C C T T T A C A A A G G C C C C C T G A G A C A G G G A G C A -
GCCCGCCGGCGGCCAACCAAACTCTTGTTTCTACAGTGAATCTCTGAGACAAAACATAAATGAATCAAAAC
TTTCAACAACGGATCTCTTGGTTCTGGCATCGATGAAGAACGCAGCGAAATGCGATAAGTAATGTGAATTG
CAGAATTCAGTGAATCATCGAATCTTTGAACGCACATTGCGCCCTCTGGTATTCCGGAGGGCATGCCTGTTC
GAGCGTCATTTCAACCCTCAAGCCTGGCTTGGTGATGGGGCACTGCTCTCACGGGAGCAGGCCCTGAAATC
T A G T G G C G A G C T C G C T A G G A C C C C G A G C G T A G T A G T - - - T A T A - T C T C G T -
T C T G G A A G G C C C T G G C G G T G C C C T - G C C G T T A A A - C C C C C A A -
C T T C T G A A A A T T T G A C C T C G G A T C A G G T A G G A A T A C C C G C T G A A C T T A A G C A T A -
A G A A G G A A G G T C A G T A A A T A C C A C -
A C T G C A G C T T G C G C T C C A T A T C C C T C C T G T G C A C A T A G A A A C C G T T G G C G T G C -
G C T G T C T G C G A C G C T C C G T C A C A C C T G T A A A -
GCATTTTCACCCCTCCCTCTGGATTTTCCATTTTCAGTGCGGGTGCGGGGTGCGCTTATCAGGCCGCTTATCT
CACACATC---AACCCTGTCGCACC------TTATCGCCAGTGACA-CACCAATAGCATCACTTTCAT-TCCCACTT-
T C T G C T C C A G - - - - - - - - - - - A G A G C T A T C T C C A A A A T C A T G C T G A C T - -
CTTTCTACACAGCCGCCGAGCTGGGTAAGGGT--
- - -
GTGCTGCTTTCTGGTGCGTCGCAGCTCCCAGCTCCAAGCCTACCACCGCGACCCTCGACGCGCGACAAGGC
GAGCTCGAAGCATCGATACTGACCTCGGTCCTTTAGGCAAACCATCTCTGGCGAGCACGGTCTCGACAGCA
A T G G C G T G T A T G C A C C T C C T A T C C C C T G C C C G - T G G C C T C G T C C T C T C C T C - -
GCTTGGCACTGATGATCGCACAGTTACAACGGCACTTCCGAGCTCCAGCTCGAGCGCATGAACGTCTACTT
CAACGAGGTAAGTCAATAGCCACGTCGTCAATTCAAACACCTCTGGCATGGTCAACTGCTGCCGCCAAGCC
TTTGCTAACGCGTTTTCGCCCAGGCCTCCGGCAACAAGTATGTGCCCCGCGCCGTCCTCGTCGATCTCGAGC
CCGGTACCATGGACGCCGT

>KJ197286.1 Diaporthe middletonii strain BRIP 54884e

C C C T T T G T G A A C T T A T A C C - T A -
T T G T T G C C T C G G C G T T A G G C C G G C C T C C A C T G A G G C C C C C T G A A A C A G G G A G C A -
GCCCGCCGGCGGCCAACTAAACTCTTGTTTCTATAGTGAATCTCTGAGAAAAAACATAAATGAATCAAAAC
TTTCAACAACGGATCTCTTGGTTCTGGCATCGATGAAGAACGCAGCGAAATGCGATAAGTAATGTGAATTG

CAGAATTCAGTGAATCATCGAATCTTTGAACGCACATTGCGCCCCCTGGTATTCCGGGGGGCATGCCTGTTC

GAGCGTCATTTCAACCCTCAAGCCTGGCTTGGTGATGGGGCACTACTTCCACGGGAGTAGGCCCTGAAATT

C A G T G G C G A G C T C G C C A G G A C C C C G A G C G T A G T A G T - - - T A T A - T C T C G C -

T T T G G A A G G C C C T G G C G G T G C C C T - G C C G T T A A A - A C C C C A A -

C T T C T G A A A A T T T G A C C T C G G A T C A G G T A G G A A T A C C C G C T G A A C T T A A - - - - - -

AGAAGGAAGGTCAGTAAACATCACATCCGCATCATGAACCCCAACCCCGCCTCACGCACATCGTCACCATG

G G C G C G C G A C T T T T T G C G C G G C T G C G T C A C C C - - -

GTCAACCATTTTCGCCCCTCCCTCTGGATTTTTCATTTTCAGTGCGGGTGCGGGGTGTGCTTATCAGGCCGCT

T A T C T C C C A C A C G C A A A A C C C T G T G G C A T C A A T C A C T C C T T G -

C A C T G C C A G C T C C A A C A T C A C A A T C A T C A A A T A C C A T C A A C C T C T - - - - - - - - - - -

ACTTGCGCAACGTTCGGACAATCATGCTGACTTACTTCCCACACAGCCGCCGAGCTGGGCAAGGGTTTGCG

T A A G T T G C C T C C T G T C A A C A T C G C - C C G A C C T T A T C G C C A C C C C - -

G T A G C T G A C A C G T T T C C C A G G G T A A C C A A A T C G G T G C T G C T T T C T G G T G C G T A C C A G C T C -

CAGCTCCGAGCCTGCCACCGCGACACTCGACGCGCGACAATACTAGCTCGCAATATCGTTGCTGACCTCGT

CTCTTCAGGCAAACCATCTCTGGCGAGCACGGTCTCGACAGCAATGGCGTGTATGTACCTCCTATTCCCTGC

C G A C C G A T C T C A T C C T C T C C T C - -

GCTTGGCACTGATGATCGCACAGTTACAACGGCACTTCCGAGCTCCAGCTCGAGCGCATGAACGTCTACTT

C A A C G A G G T A A G C C T A T G G C C A C G T C T T C G A T C C A A G - -

CGTCTGGCATGGTTTACTGCCGCCGCCAGGACCTTGCTAATGCGCTCTCGCCCAGGCCTCCGGCAACAAGT

ATGTGCCTCGCGCCGTCCTCGTCGATCTCGAGCCCGGTACCATGGACGCCGT

>KJ197282.1 Diaporthe miriciae strain BRIP 54736j

C C C T T T G T G A A C T T A T A C C - T A T T T G T T G C C T C G G C G -

T A G G C C G G C C T C T A C T G A G G C C C C C T G A G A C A G G G A G C A -

GCCCGCCGGCGGCCAACTAAACTCTTGTTTCTATAGTGAATCTCTGAGAAAAAACATAAATGAATCAAAAC

TTTCAACAACGGATCTCTTGGTTCTGGCATCGATGAAGAACGCAGCGAAATGCGATAAGTAATGTGAATTG

CAGAATTCAGTGAATCATCGAATCTTTGAACGCACATTGCGCCCTCTGGTATTCCGGAGGGCATGCCTGTTC

G A G C G T C A T T T C A A C C C T C A A G C C T G G C T T G G T G A T G G G G C A C T G C C T T C G A G - -

GGCAGGCCCTGAAATCTAGTGGCGAGCTCGCTAGGACCCCGAGCGTAGTAGT---TATA-TCTCGT-

T C T G G A A G G C C C T G G C G G T G C A C T - G C C G T T A A A - C C C C C A A -

C T T C T G A A A A T T T G A C C T C G G A T C A G G T A G G A A T A C C C G C T G A A C T T A A G C A T - - -

G A A G G A A G G T T A G T A A A T A T C A C -

A T T G C T G C T T G C G C T C C A T A T C C T T C C C A T G C A C A T G G G A A T C G T T G C C G T G C -

G C T G T C T G C G A C G C T T C G T C A C A - - - -

GTCAAGCATTTTCACCCCTCCCTCTGGATTTTCCATTTTCAGTGCGGGTGCGGGGTGCGCTTATCAGGCCGC

TTATCTCACACATC--AAACCCTGTCGCACC------CTGTCACCAGTGACA-CACCAATAGCGTCACTTTCAT-
TCCCATTT-GTTGCTCCAG-----------GAAGCTATCTCGAAAATCATGCTGACT--
TTTTCTACACAGCCGCCGAGCTGGGCAAGGGTTTGCGTAAGTTGCTCCCTGTCAACACCGC-
CGGACCTTATCGCCACCC---
GTAGCTGACACGTTTACCAGGGTAACCAAATCGGTGCTGCTTTCTGGTGCGTCGCAGCTCCCAGCTCCAAG
CCTACCGCCGCGACCCTCGACGCGCGACAAGGCGAGCTCGAAGCATCGATACTGACCTCGGTTCTTTAGGC
AAACCATCTCCGGCGAGCACGGTCTCGACAGCAATGGCGTGTATGCACCTCCTATTCCCTGCCCG-TGGC-
TCATCCTCTTCTC--
GCTTGGCACTGATGATCGCACAGTTACAACGGCACTTCCGAGCTCCAGCTCGAGCGCATGAACGTCTACTT
CAACGAGGTCAGTCAATAGCCACGTCGTCAATTCAAA-
ACCTCTGGCATGGTCAACTGCCGCCGCCAAGCCCCTGCTAACGCGTTTTCGCCCAGGCCTCCGGCAACAAG
TATGTGCCCCGCGCCGTCCTCGTCGATCTCGAGCCCGGTACCATGGACGCCGT

>KC343162.1 Diaporthe oncostoma strain CBS 589.78

CCCTTTGTGAACTTATACCTTA-CTGTTGCCTCGGCG-
CAGGCCGGCCCCCTACGGGGCCCCTTGCGACAAGGAGCAGGCCCGCCGGCGGCCAAGTTAACTC-
TGTTTTTACACTGAAACTCTGAGACAAAACATAAATGAATCAAAACTTTCAACAACGGATCTCTTGGTTCTG
GCATCGATGAAGAACGCAGCGAAATGCGATAAGTAATGTGAATTGCAGAATTCAGTGAATCATCGAATCTT
TGAACGCACATTGCGCCCTCTGGTATTCCGGAGGGCATGCCTGTTCGAGCGTCATTTCAACCCTCAAGCCTG
GCTTGGTGTTGGGGCACTGCCTCCGCGGGGGCAGGCCCTGAAATACAGTGGCGAGCTCGCCAGGACTCCGA
GCGCAGTAGT---TAAACCCTCGC-TCTGGAAGG-CCTGGCGGTGCCCT-GCCGTTAAA--CCCCAA-
CTCCTGAAAATTTGACCTCGGATCAGGTAGGAATACCCGCTGAACTTAAGCATAGAGAAGGAAGGTTAGTA
AACATCGCAATCACAAAATCACTCACCTTGTCCTCGCATTTTGCCCCATATCACCAGCGTGCGGCTGCTCTC
GCGGCTTCTTCGCACTGGGAGGCGCATTTTGACCCCTCCCTCTGGATTTTCCATTTTCAGTGCGGGTGCGGG
GTGCGCTTATCAGGGAGCTTATCTTTTCCG--CAAACCCCTGTTACATCACCCCTC----------
CCACCACCAATACGCGTACCATGAA-CCTCATCGTCCGCATTCAAT-ACT-
ACGTGTAAATCGCGTCGAAATCCATGCTGACCTTTTGTTTATACAGCTGCCGAGCTTGGCAAGGGTTTGCGT
AAGTTGCT-CCTGTCAACACCAC-
CGGACCTTATCATCGCCACCTGTAGCTGACACGTTTCCCAGGGTAACCAAATCGGTGCTGCTTTCTGGTGC--
GTCC------AGCTCCAAGTCAACCACCGCGACGTC-
GACGCGCGACAATACGACCTTGAAGCATCGTTACTGACCTCGACTT-
TCAGGCAAACCATCTCTGGCGAGCACGGTCTCGACAGCAATGGCGTGTATGCACCTCCTATTTCCTGTCTTC
AATCTCGTCCTT--
TGCCGGCTTGGCACTGACAATCACACAGTTACAACGGCACTTCCGAGCTCCAGCTCGAGCGCATGAACGTC
TACTTCAACGAGGTAAGTCAACAGCCACGTTG-----TTGGCCATCCACAACCCGAATTCTTGCCGTCGCTGG--

CTTGGCTAACGCGTTACCGCCCAGGCCTCCGGCAACAAGTATGTGCCCCGCGCCGTCCTCGTCGATCTCGA
GCCCGGTACCATGGACGCCGT

>KP267863.1 Diaporthe oraccinii strain LC3166

CCCTTTGTGAACTTATACCTTA-CTGTTGCCTCGGCG-AAGGCTGGCCCCCCCCGGGGCCCCTC--
GGTGTTGAGACAGCCCGCCGGCGGCCAAGTTAACTCTTGTTTTTACACTGAAACTCTGAGATAAAACATAA
ATGAATCAAAACTTTCAACAACGGATCTCTTGGTTCTGGCATCGATGAAGAACGCAGCGAAATGCGATAAG
TAATGTGAATTGCAGAATTCAGTGAATCATCGAATCTTTGAACGCACATTGCGCCCTCTGGTATTCCGGAGG
GCATGCCTGTTCGAGCGTCATTTCAACCCTCAAGCCTGGCTTGGTGATGGGGCACTGCTCTTCAAAGAGCA
GGCCCTGAAATTCAGTGGCGAGCTCGCCAGGACCCCGAGCGCAGTAGT---TAAACCCTCGC-
TCTGGAAGGCCCTGGCGGTGCCCT-GCCGTTAAA-CCCCCAA-CTTCTGAAA--
- -
GAGAAGGAAGGTTAGTAAACACCACAATCACGGAACATGCTACCCTGCCCTCCTACTGCACCTCAAACCAT
GAGCCCGCAGCTGCTCGTTTGGCTTCGTCATAACGGGGGGCGCATTTTCACCCCTCGCTTTGGATTTTCCAT
TTTCAGTGCGGGTGCGGGGTGCGCTTATCAGGGGGCTTATCTCCTACTCCCAAAACCCTGTTGCATCACTCA
CC-CCTGCCACCACCACTACCAATACGCCCACCATGAA-TCCCACCGCCCTCCTTCAAT-TCA-
ACTCGTGAAGCGCGTTGAACATTATGCTGACCCTCTATCTACACAGCCGCCG---------------------------------------
----
A C C T C G A A A C A T C G T T G C T G A C C T C G A C T C -
TTAGGCAAACCATCTCTGGCGAGCACGGCCTCGACAGCAATGGCGTGTATGCACCTCCTATTCCCTGCCCA
CTGATCTTGGCCTCTTTTCCGGCTTGGCACTGACAATTGCGCAGTTACAACGGCACTTCTGAGCTCCAGCTC
GAGCGCATGAACGTCTACTTCAACGAGGTAAGTAAAAAACCACAGCAACCATTT-
G A C A T C T G T A A C A T G G T T T C C T G C C G T T G C T C G G A C C T -
GCTAACGCGTTATCGCCCAGGCCTCCGGCAACAAGTATGTTCCTCGCGCCGTTCTCGTCGATCTCGAGCCCG
GTACCATGGACGCCGT

>KP714505.1 Diaporthe penetriteum isolate LC3353

CCCTTTGTGAACTTATACCTTA-CTGTTGCCTCGGCG-CTAGCTGGTCCTC--CGGGGCCCCTC-----
GAGGAGCAGGCACGCCGGCGGCCAACCTAACTCTTGTTTTTACACTGAAACTCTGAGATAAAACATAAATG
AATCAAAACTTTCAACAACGGATCTCTTGGTTCTGGCATCGATGAAGAACGCAGCGAAATGCGATAAGTAA
TGTGAATTGCAGAATTCAGTGAATCATCGAATCTTTGAACGCACATTGCGCCCTCTGGTATTCCGGAGGGC
ATGCCTGTTCGAGCGTCATTTCAACCCTCAAGCCTGGCTTGGTGATGGGGCACTGCCTGTAAAAGGGCAGG
CCCTGAAATTCAGTGGCGAGCTCGCCAGGACCCCGAGCGCAGTAGT---TAAACCCTCGC-
TCTGGAAGGCCCTGGCGGTGCCCT-GCCGTTAAA-CCCCCAA-CTTCTGAAA--

- -

GAGAAGGAAGGTTAGTAAACACCACAATCTCGGAACATGCTATCCTGCCCTCCTACTGCACCTCAAATCAT

CAGCCCGCAGCTGCTCGGGCGGCTTCGTCATATCGGGGGGCGCATTTTCACCCCTCGCTTTGGATTTTCCAT

TTTCAGTGCGGGTGCGGGGTGCGCTTATCAGGGGGCTTATCTCCTACACCCAAAACCCTGTTGCATCACTCA

CTCCTTCTAACCACCCCCACCAATACGCTCATCATGAA-TTCCATCG-CCTCCTTCAATAACCGTTCG-

CAAAACGCGTTGAATTCCATGCTGACTTTCTATTTCCACAGCCGCCG--

ACCTCGAAGCATCGTTACTGACCTCGAATTCTTAGGCAAACCATCTCTGGCGAGCACGGCCTCGACAGCAA

TGGCGTGTATGCACCTCCTATTCCCTGCCCACTGATCTTGGCCTCTCTTCCGGCTTGGCACTGACAATTGCAC

AGTTACAACGGCACTTCTGAGCTCCAGCTCGAGCGCATGAACGTCTACTTCAACGAGGTAAGTCAACAGCC

ACATCATCCGTCCGACCATCTCCAACATGGTTTACTGCTGTCGCCCGGACCTTGCTAACGCGTTATCGCCCA

GGCCTCCGGCAACAAGTATGTTCCCCGCGCCGTCCTCGTCGATCTCGAGCCCGGTACCATGGACGCCGT

>KC343181.1 Diaporthe pseudomangiferae strain CBS 101339

CCCTTTGTGAACTTATACC-TT-TTGTTGCCTCGGCG-CATGCTGGCCTCT-AGTAGGCCCCTC--

GGTGAGGAGAAGGCACGCCGGCGGCCAAGTTAACTCTTGTTTTTACACTGAAACTCTGAGAAAAAACACA

AATGAATCAAAACTTTCAACAACGGATCTCTTGGTTCTGGCATCGATGAAGAACGCAGCGAAATGCGATAA

GTAATGTGAATTGCAGAATTCAGTGAATCATCGAATCTTTGAACGCACATTGCGCCCTCTGGTATTCCGGAG

GGCATGCCTGTTCGAGCGTCATTTCAACCCTCAAGCATTGCTTGGTGTTGGGGCACTGCTTCTAACGAAGCA

GGCCCTGAAATCTAGTGGCGAGCTCGCCAGGACCCCGAGCGTAGTAGT---TAAACCCTCGC-

TCTGGAAGGCCCTGGCGGTGCCCT-GCCGTTAAA-CCCCCAA-

CTTTTGAAAATTTGACCTCGGATCAGGTAGGAATACCCGCTGAACTTAAGCATAGAGAAGGAAGGTTAGTA

AACATCACAATCATAAAATGCTCTCCTCTGCCCTCACCTTTTGCCTCAGATCACCAGTGTGCGGCTGTTGGT

ACAGCTTCGTCATACTGAGGGGCGCATTTTCACCCCTCGCTCTGGATTTTCCATTTTCAGTGCGGGTGCGGG

GTGTGCTTATCAGGGAGCTTATCTCCTACACCCAAAACCCTGTTGCAACAACAAC----------

CCTACCGCCACTTCATTCATCCTCAACCCCCATCGACATCCTTGAAT--CAATCAAGCAAACCGC-

TAGGAAATCATGCTGACCTTTTCTCTACATAGCCGCCGAGCTTGGTAAGGGTTTGCGTAAGTTGCT-

CCTGTCAACACCGA-

CGGACCTTATCATCGCCACTCGTAGCTGACACGTTTCCCAGGGTAACCAAATCGGTGCTGCTTTCTGGTGC--

GTCC-------------------ACCACCGCGAGCTC-

GACGCGCGACAATACGACCTTGAACCATCGTTACTGACCTCGACTC-

TCAGGCAAACCATCTCTGGCGAGCACGGCCTCGACAGCAATGGCGTGTATGCACCTCCTATCCCCTACCTC

CAGAACTCGTCCTCCCTGCCGGCTTGGCACTGACAATCATACAGTTACAACGGCACTTCCGAGCTCCAGCTC

GAGCGCATGAACGTCTACTTCAACGAGGTAAGTCAACAGCCACGTCG-----

TTGGCCATCTGCAGCACGGTTTGCTGCCGTCACTGGAGCCTTGCTAACGCGTTATCGCACAGGCCTCCGGCA
ACAAGTATGTCCCTCGCGCCGTCCTCGTCGATCTCGAGCCCGGTACCATGGACGCCGT

>KC343184.1 Diaporthe pseudophoenicicola strain CBS 462.69

CCCTTTGTGAACTTATACCTTA-CTGTTGCCTCGGCG-CATGCTGGCCCCC-
TCGGGGTCCCCTGAGACAGGGAGCAGGCACGCCGGCGGCCAAGTTAACTCTTGTTTTTACACTGAAACTCT
GAGAAAAAACACAAATGAATCAAAACTTTCAACAACGGATCTCTTGGTTCTGGCATCGATGAAGAACGCA
GCGAAATGCGATAAGTAATGTGAATTGCAGAATTCAGTGAATCATCGAATCTTTGAACGCACATTGCGCCC
TCTGGTATTCCGGAGGGCATGCCTGTTCGAGCGTCATTTCAACCCTCAAGCATTGCTTGGTGTTGGGGCACT
GCTTTTCACCAAGCAGGCCCTGAAATCTAGTGGCGAGCTCGCCAGGACCCCGAGCGTAGTAGT---
TAAACCCTCGC-TTTGGAAGGCCCTGGCGGTGCCCT-GCCGTTAAA-CCCCCAA-
CTTCTGAAAATTTGACCTCGGATCAGGTAGGAATACCCGCTGAACTTAAGCATAGAGAAGGAAGGTTAGTA
AACATCACAATCATGAAATGCTCTCCTCTGCCCTCACCTTTCGCCTCAGATCATCAGTGTGCGGCTGTTTGT
ACAGCTTCGTCATACTGAGGGGCGCATTTTCACCCCTCGCTCTGGATTTTCCATTTTCAGTGCGGGTGCGGG
GTGCGCTTATCAGGGAGCTTATCTCCTACACCCAAAACCCTGTTGCAACAACAACTTCTTGCAGTTGCTACC
ACCACTTCATTCATCCTCAACCCCCATCGACGTCCTTGAAT-CAA-TCAAGCAAACCGCCAGG-
AAATCATGCTGACTTTCTATCTACATAGCCGCCGAGCTTGGCAAGGGTTTGCGTAAGTTGCT-
CCTGTCAACACCGC-
CGGACCTTATTATCGCCACTCGTAGCTGACACGTTTCCCAGGGTAACCAAATCGGTGCTGCTTTCTGGTGC--
GTCC-------------------ACCGCCGCGAGCTC-
GACGCGCGACAATACGGCCTCGAACCATCTTTACTGACCTCGACTC-
TCAGGCAAACCATCTCTGGCGAGCACGGCCTCGACAGCAATGGCGTGTATGCACCTCCTATTCCCTGCCTTC
AGATCTCGTCCTCCCTGCCGGCTTGGCACTGACAATCATACAGTTACAACGGCACTTCCGAGCTCCAGCTCG
AGCGCATGAACGTCTACTTCAACGAGGTAAGTCAACAGCCACGTCG-----
TTGGCCATCTGCAGCACGGTTTGCTGCCGTCGCCGGGGCCTTGCTAACGCGTTATCGCACAGGCCTCCGGCA
ACAAGTATGTCCCTCGCGCCGTCCTCGTCGATCTCGAGCCCGGTACCATGGACGCCGT

>KY852495.1 Diaporthe sambucusii strain CFCC 51986

CCCTTTGTGAACTTATACC-CA-CTGTTGCCTCGGCG-CAGGCCGGTCTGT-
CTCAGACCCCCCTGAAACAGGGAGCA-GCCCGCCGGCGGCCAACCAAACTC-
TGTTTCTATAGTGAATCTCTGAGAAAAAACATAAATGAATCAAAACTTTCAACAACGGATCTCTTGGTTCTG
GCATCGATGAAGAACGCAGCGAAATGCGATAAGTAATGTGAATTGCAGAATTCAGTGAATCATCGAATCTT
TGAACGCACATTGCGCCCTCTGGTATTCCGGAGGGCATGCCTGTTCGAGCGTCATTTCAACCCTCAAGCCTG
GCTTGGTGATGGGGCAGTGCCTTGGACAAGGCACGCCCTGAAATTCAGTGGCGAGCTCGCCAGGACCCCGA

GCGTAGTAGT---TACA-TCTCGC-TCTGGAAGGCCCTGGCGGTGCCCT-GCCGTTAAA-CCCCCAA-

C T T C T G A A A -

GGAAGGTCAGTAAACATTAAACTCACAACATCAACCCCAAGTCATTCCAGTGCACATCGCGATCATCGGCG

C G C G A C C G T C T G C G C C G C T T C G T C A C T C - - -

GTCACGCATTTTCACCCCTCGTTCTGGATTTTCCACTTTCAGTGCGGGTGCGGGGTGTGCTTATCAGGCCGC

TTATCGCTTGCGTTCAAAACCCTGTGGCATCAACCACTCTTTGCCGCTGCCCACATTAACGCCAACGCCATC

A A - T C T C A T C A C C C C A C C C C C - - - - - - -

TCTGACAAGACCTGAGGAAAACCATGCTGACTCGCTTTCCATACAGCCGCCGAGCTGGGTAA-----

T G C G T A A G T T G C T C C C T G T C A A C A C T G C - C C G A C C T T A T C G C C A C C C - - -

GTAGCTGACACGTTTCCCAGGGTAACCAAATCGGTGCTGCTTTCTGGTGCGTCCCAGCTCCCAGCTCCAGGC

CTACCGGCGCGATGCTCGACGCGCGACAAGACCAGCTCGCAACATTGTTACTGACCTCGGCTCTGTAGGCA

AACCATCTCTGGCGAGCACGGCCTCGACAGCAATGGCGTGTATGCACCTCCTATTCCCTGCCCACTGATCTC

G T C C T G T C C T C - -

GCTTGACACTGATGATTGCACAGTTACAACGGCACTTCCGAGCTCCAGCTCGAGCGCATGAACGTCTACTT

CAACGAGGTATGTCAATGGCCACGTCGTCAATCCAAACATCTACTGCGCGGCGTGCTGCCGTAGCCAAAGC

CTTGCTAACGCGTTGTCGCCCAGGCTTCCGGCAACAAGTATGTGCCCCGCGCTGTCCTCGTCGATCTCGAGC

CCGGTACCATGGACGCCGT

>KY852496.1 Diaporthe sambucusii strain CFCC 51987

C C C T T T G T G A A C T T A T A C C - C A - C T G T T G C C T C G G C G - C A G G C C G G T C T G T -

C T C A G A C C C C C T G A A A C A G G G A G C A - G C C C G C C G G C G G C C A A C C A A A C T C -

TGTTTCTATAGTGAATCTCTGAGAAAAAACATAAATGAATCAAAACTTTCAACAACGGATCTCTTGGTTCTG

GCATCGATGAAGAACGCAGCGAAATGCGATAAGTAATGTGAATTGCAGAATTCAGTGAATCATCGAATCTT

TGAACGCACATTGCGCCCTCTGGTATTCCGGAGGGCATGCCTGTTCGAGCGTCATTTCAACCCTCAAGCCTG

GCTTGGTGATGGGGCAGTGCCTTGGACAAGGCACGCCCTGAAATTCAGTGGCGAGCTCGCCAGGACCCCGA

GCGTAGTAGT---TACA-TCTCGC-TCTGGAAGGCCCTGGCGGTGCCCT-GCCGTTAAA-CCCCCAA-

C T T C T G A A A -

GGAAGGTCAGTAAACATTAAACTCACAACATCAACCCCAAGTCATTCCAGTGCACATCGCGATCATCGGCG

C G C G A C C G T C T G C G C C G C T T C G T C A C T C - - -

GTCACGCATTTTCACCCCTCGTTCTGGATTTTCCACTTTCAGTGCGGGTGCGGGGTGTGCTTATCAGGCCGC

TTATCGCTTGCGTTCAAAACCCTGTGGCATCAACCACTCTTTGCCGCTGCCCACATTAACGCCAACGCCATC

A A - T C T C A T C A C C C C A C C C C C - - - - - - -

TCTGACAAGACCTGAGGAAAACCATGCTGACTCGCTTTCCATACAGCCGCCGAGCTGGGTAA-----

T G C G T A A G T T G C T C C C T G T C A A C A C T G C - C C G A C C T T A T C G C C A C C C - - -

GTAGCTGACACGTTTCCCAGGGTAACCAAATCGGTGCTGCTTTCTGGTGCGTCCCAGCTCCCAGCTCCAGGC

CTACCGGCGCGATGCTCGACGCGCGACAAGACCAGCTCGCAACATTGTTACTGACCTCGGCTCTGTAGGCA
AACCATCTCTGGCGAGCACGGCCTCGACAGCAATGGCGTGTATGCACCTCCTATTCCCTGCCCACTGATCTC
G T C C T G T C C T C - -
GCTTGACACTGATGATTGCACAGTTACAACGGCACTTCCGAGCTCCAGCTCGAGCGCATGAACGTCTACTT
CAACGAGGTATGTCAATGGCCACGTCGTCAATCCAAACATCTACTGCGCGGCGTGCTGCCGTAGCCAAAGC
CTTGCTAACGCGTTGTCGCCCAGGCTTCCGGCAACAAGTATGTGCCCCGCGCTGTCCTCGTCGATCTCGAGC
CCGGTACCATGGACGCCGT

>KU712439.1 Diaporthe tectonendophytica voucher MFLUCC 13-0471

C C C T T T G T G A A C T T A T A C C - T A - C T G T T G C C T C G G C G -
C A G G C C G G C C T C T G C T G A G G C C C C C T G A G A C A G G G A G C A -
GCCCGCCGGCGGCCAACTAAACTCTTGTTTCTTTAGTGAATCTCTGAGAAAAAACATAAATGAATCAAAAC
TTTCAACAACGGATCTCTTGGTTCTGGCATCGATGAAGAACGCAGCGAAATGCGATAAGTAATGTGAATTG
CAGAATTCAGTGAATCATCGAATCTTTGAACGCACATTGCGCCCTCTGGTATTCCGGAGGGCATGCCTGTTC
GAGCGTCATTTCAACCCTCAAGCCTGGCTTGGTGATGGGGCACTGCTCTCGCGGGAGCAGGCCCTGAAATC
T A G T G G C G A G C T C G C C A G G A C C C C G A G C G T A G T A G T - - - T A T A - T C T C G T -
TCTGGAAGGCCCTGGCGGTGCCCT-GCCGTTAAA-CCCCCAA-CTTCTGAAAATT-GACCTCGGATCAGGTAG-
- G A G A A G G A A G G T T A G T A A A T A T C A T -
A T C G C A G C T T G C G C T C C A C A T C C C T C C T A T G C A C A T A G A G A T C G T T G G T G T G C -
G C T G T C T G C G A C G C T T C G T C A C A - - - -
G T C A A G C A T T T T C A C C C C T C C C T C T G G A T T T T C C A T T T T C A G T G C G G G T G C G G G G T G C G C T -
A T C A G G C C G C T T A T C T C A C A C A T C - - - A A C C C T G T C G C A C C - - - - - - T T A T C A C C A G T G A C A -
TACCAATAGCATCACTTTCATTCCCCACTG--CTGCTCCAG----------GGAGCTATCTTGAAAATCATGCTGAC--
----
C T C C - A G C T C C A -
GCCTACCACCGCGACCCTCGACGCGCGACAAGGCGAGCTCGAAGCATCGATACTGACCTCGGTTCTTTAGG
CAAACCATCTCTGGCGAGCACGGTCTCGACAGCAATGGCGTGTATGTACCTCCTATTCCCTGCCCACTGCCC
TCGTCCTCTCCTCCGGCTTGGCACTGATGATCGCGCAGTTACAACGGCACTTCCGAGCTCCAGCTCGAGCGC
A T G A A C G T C T A C T T C A A C G A G G T A A G T C A A T A G C C A C G T C G C C A A T T C A A T C G T C T -
CGGTATGATGAACTGCCGCCGCCAAGCCATTGCTAACGCGTTTTCCCCCAGGCCTCCGGCAACAAGTATGT
GCCCCGCGCCGTCCTCGTCGATCTCGAGCCCGGTACCATGGATGCCGT

>KU712429.1 Diaporthe tectonigena voucher MFLUCC 12-0767

CCCTTTGTGAACTTATACCTTA-CTGTTGCCTCGGCG-
CATGCTGGCCCCCCTGGGGGTCCCTCGAGACGAGGAGCAGGCACGCCGGCGGCCAAGTTAACTCTTGTTTT
TACACTGAAACTCTGAGAAGAAACACAAATGAATCAAAACTTTCAACAACGGATCTCTTGGTTCTGGCATC
GATGAAGAACGCAGCGAAATGCGATAAGTAATGTGAATTGCAGAATTCAGTGAATCATCGAATCTTTGAAC
GCACATTGCGCCCTCTGGTATTCCGGAGGGCATGCCTGTTCGAGCGTCATTTCAACCCTCAAGCATTGCTTG
GTGTTGGGGCACTGCTTTTAAA--
AGCAGGCCCTGAAATCTAGTGGCGAGCTCGCCAGGACCCCGAGCGCAGTAGT---TAAACCCTCGC-
TCTGGAAGGCCCTGGCGGTGCCCT-GCCGTTAAA-CCCCCAA-CTTTGAAAA--TTGACCTCGGATCAGGTAG--
----
GAGAAGGAAGGTTAGTAAACATCACAATCATAGAACATGCTACCCTATCCCCATACTTTGCCTCAAATCAT
CAGTGTGCGGCTGTTGGTATGGCTTCGTCATACTGAGGGGCGCATTTTCACCCCTCGCTTTGGATTTTCCA-
TTTCAGTGCGGGTGCGGGGTGCGCTTATCAGGGAGCTTATCTCCTACAC-
CAAAACCCTGTTGCAACAACAACTCCTTGCCGCTACTACCACCACTTCATTCACCCTCAACCCTCATCGGCA
CCCTTCAAT--CGATCAAGTAAACGGTCGCGAAAATC-TGCTGAC--
---CGAGCT-
CGACGCGCGACAATACGACTTCGATCCATCGTCACTGACCTCGACTC-
TCAGGCAAACCATCTCTGGCGAGCACGGCCTCGACAGCAATGGCGTGTATGCACCTCCTATTCCCTACCTCC
AGATCTCGTCCTCCCTGCCGGCTTGGCACTGACAATCATACAGTTACAACGGCACTTCCGAGCTCCAGCTCG
AGCGCATGAACGTCTACTTCAACGAGGCAAGTCAACAGCCACGTCG-----
TTGACCATCTGCAGCACGGTTTCCTGCCGTCGCCAAGGCTTTGCTAACGCGTTATCGCCCAGGCCTCCGGCA
ACAAGTATGTTCCTCGCGCCGTCCTTGTCGATCTCGAGCCCGGTACCATGGACGCCGT

>OQ917074.1 Diaporthe tuyouyou strain BRIP 75017a

CCCTTTGTGAACTTATACCTTA-CTGTTGCCTCGGCG-CATGCTGGCCCCC-
TCGGGGTCCCTTGAGACAAGGAGCAGGCACGCCGGCGGCCAAGTTAACTCTTGTTTTTACACTGAAACTCT
GAGAAAAAACACAAATGAATCAAAACTTTCAACAACGGATCTCTTGGTTCTGGCATCGATGAAGAACGCA
GCGAAATGCGATAAGTAATGTGAATTGCAGAATTCAGTGAATCATCGAATCTTTGAACGCACATTGCGCCC
TCTGGTATTCCGGAGGGCATGCCTGTTCGAGCGTCATTTCAACCCTCAAGCATTGCTTGGTGTTGGGGCACT
GCCTGTAAAAGGGCAGGCCCTGAAATCTAGTGGCGAGCTCGCCAGGACCCCGAGCGCAGTAGT---
TAAACCCTCGC-TTTGGAAGGCCC-GGCGGTGCCCT-GCCGTTA---

AAGGTTAGTAAACATCACAATTATAGAACATGCCACCCTGTCCTCACATTTTGCCTCAAATCACCAGTGTGC
GGCTGTTGGTACGGCTTCGTCATACTGAGGGGCGCATTTTCACCCCTCGCTCTGGATTTTCCA-
TTTCAGTGCGGGTGCGGGGTGCGCTTATCAGGGAGCTTATCTCCTACACCCAAAACCCTGTTGTAACAACA
ACACCATGCCGTTCCTT---------CATTCACCCTCAAACCCCACCGATATCCTTGAAT--

TGATCAAGCAAACCGCCTCGAA-
ATCATGCTGACATCGTATCTACACAGCCGCCGAGCTTGGCAAGGGTTTGCGTAAGTTGCT-
CCTGTCAACACCAA-
CGGACCTTATCATCGCCACCTGTAGCTGACACGTTTCCCAGGGTAACCAAATCGGTGCTGCTTTCTGGTGC--
GTCCCAGCCCGGCTCCAACGCCACCACCGCGAGCTC-
GACGCGCGACAATACGACCTCGAACCATCGTTACTGACCTCGACTC-
TCAGGCAAACCATCTCTGGCGAGCACGGCCTCGACAGCAATGGCGTGTATGCACCTCCTATTCCCTACGCT
CATATCTTGTCCTCCCTGCCGGCTTGGCACTGACAATCACACAGTTACAACGGCTCTTCCGAGCTCCAGCTC
GAGCGCATGAACGTCTACTTCAACGAGGTAAGTCAACAGCCACGTCG-----
TTGACCATCTGCAGCACGGTTTCCTGCCGTCGCCAAGGCCTTGCTAACGCGTTATCGCCCAGGCCTCCGGCA
ACAAGTATGTTCCTCGCGCCGTCCTCGTCGATCTCGAGCCCGGTACCATGGACGCCGT

>KJ490587.1 Diaporthe unshiuensis isolate ZJUD52

CCCTTTGTGAACTTATACC-TA-TTGTTGCCTCGGCG-
CAGGCCGGCCTCTACTGAGGCCCCCTGAGACAGGGAGCA-
GCCCGCCGGCGGCCAACCAAACTCTTGTTTCTACAGTGAATCTCTGAGACAAAACATAAATGAATCAAAAC
TTTCAACAACGGATCTCTTGGTTCTGGCATCGATGAAGAACGCAGCGAAATGCGATAAGTAATGTGAATTG
CAGAATTCAGTGAATCATCGAATCTTTGAACGCACATTGCGCCCTCTGGTATTCCGGAGGGCATGCCTGTTC
GAGCGTCATTTCAACCCTCAAGCCTGGCTTGGTGATGGGGCACTGCTCTCACGAGAGCAGGCCCTGAAATC
TAGTGGCGAGCTCGCTAGGACCCCGAGCGTAGTAGT---TATA-TCTCGT-
TCTGGAAGGCCCTGGCGGTGCCCT-GCCGTTAAA-CCCCCAA-CTTCTGAAAATT----------------------------------
-----GAGAAGGAAGGTTAGTAAATACCAC-
ACTGCAGCTTGCGCTCCATATCCCTCTTATGCACATAGAAACCGTTGGCGTGC-
GCTGTCTGCGACGCTCCGTCACA----
GTAAAGCATTTTCACCCCTCCCTCTGGATTTTCCATTTTCAGTGCGGGTGCGGGGTGCGCTTATCAGGCCGC
TTATCTCACACATC---AACCCTGTCGCACC------TTATCGCCAGTGACA-CACCAATAGCATCACTTTCAT-
TCCCACTT-TCTGCTCCAG-----------GGAGCTATCTCCAAAATCATGCTGACT--
CTTTCTACACAGCCGCCGAGCTGGGTAAGGGTT--

AGCTCGAAGCATCGATACTGACCTCCGTCCTTTAGGCAAACCATCTCTGGCGAGCACGGTCTCGACAGCAA
TGGCGTGTATGCACCTCCTTTTCCCTGCCCG-TGGCCTCGTCCTCTCCTC--
GCTTGGCACTGATGATCGCACAGTTACAACGGCACTTCCGAGCTCCAGCTCGAGCGCATGAACGTCTACTT
CAACGAGGTAAGTCAATAGCCAGGTCGTCAATTCAAACACCTTTGGCATGGTCAACTGCTGCCGCCAAGCC
TTTGCTAACGCGTTTTCGCCCAGGCCTCCGGCAACAAGTATGTGCCCCGCGCCGTCCTCGTCGATCTCGAGC
CCGGTACCATGGACGCCGT

>KJ490586.1 Diaporthe unshiuensis isolate ZJUD51

CCCTTTGTGAACTTATACC-TA-TTGTTGCCTCGGCG-
CAGGCCGGCCTCTACTGAGGCCCCCTGAGACAGGGAGCA-
GCCCGCCGGCGGCCAACCAAACTCTTGTTTCTACAGTGAATCTCTGAGAAAAAACATAAATGAATCAAAAC
TTTCAACAACGGATCTCTTGGTTCTGGCATCGATGAAGAACGCAGCGAAATGCGATAAGTAATGTGAATTG
CAGAATTCAGTGAATCATCGAATCTTTGAACGCACATTGCGCCCTCTGGTATTCCGGAGGGCATGCCTGTTC
GAGCGTCATTTCAACCCTCAAGCCTGGCTTGGTGATGGGGCACTGCTCTCACGAGAGCAGGCCCTGAAATC
TAGTGGCGAGCTCGCTAGGACCCCGAGCGTAGTAGT---TATA-TCTCGT-
TCTGGAAGGCCCTGGCGGTGCCCT-GCCGTTAAA-CCCCCAA-CTTCTGAAAATT------------------------------------
-----GAGAAGGAAGGTTAGTAAATACCAC-ACTGCAGCTTGCGCTCC-
ATATCCCTCTATGCACATAGAAACCGTTGGCGTGC-GCTGTCTGCGACGCTCCGTCA------
GTAAAGCATTTTCACCCCTCCCTCTGGATTTTCCATTTTCAGTGCGGGTGCGGGGTGCGCTTATCAGGCCGC
TTATCTCACACATC---AACCCTGTCGCACC------TTATCGCCAGTGACA-CACCAATAGCATCACTTTCAT-
TCCCACTT-TCTGCTCCAG-----------GGAGCTATCTCCAAAATCATGCTGACT--
CTTTCTACACAGCCGCCGAGCTGGGTAAGGGTT--

AGCTCGAAGCATCGATACTGACCTCCGTCCTTTAGGCAAACCATCTCTGGCGAGCACGGTCTCGACAGCAA
TGGCGTGTATGCACCTCCTATCCCCTGCCCG-TGGCCTCGTCCTCTCCTC--
GCTTGGCACTGATGATCGCACAGTTACAACGGCACTTCCGAGCTCCAGCTCGAGCGCATGAACGTCTACTT
CAACGAGGTAAGTCAATAGCCACGTCGTCAATTCAAACACCTCTGGCATGGTCAACTGCTGCCGCCAAGCC
TTTGCTAACGCGTTTTCGCCCAGGCCTCCGGCAACAAGTATGTGCCCCGCGCCGTCCTCGTCGATCTCGAGC
CCGGTACCATGGACGCCGT

>KX986783.1 Diaporthe xishuangbanica strain LC6707

CCCTTTGTGAACTTATACCTTA-CTGTTGCCTCGGCG-
CATGCCGGCCCCCCCGGGGGCCCCTCGAGACGAGGAGCAGGCACGCCGGCGGCCAAGTTAACTCTTGTTTT
TACACTGAAACTCTGAGAAAAAACACAAATGAATCAAAACTTTCAACAACGGATCTCTTGGTTCTGGCATC
GATGAAGAACGCAGCGAAATGCGATAAGTAATGTGAATTGCAGAATTCAGTGAATCATCGAATCTTTGAAC
GCACATTGCGCCCTCTGGTATTCCGGAGGGCATGCCTGTTCGAGCGTCATTTCAACCCTCAAGCATTGCTTG
GTGTTGGGGCACTGCTTTTCGAGAAGCAGGCCCTGAAATCTAGTGGCGAGCTCGCCAGGACCCCGAGCGCA
GTAGT---TAAACCCTCGC-TCTGGAAGGCCCTGGCGGTGCCCT-GCCGTTAAA-CCCCCAA-
CTTCTGAAAATTTGACCTCGGATCAGGTAGGAATACCCGCTGAACTT-------
GAGAAGGAAGGTTAGTAAACACCACAATCATAAAACATGCTACCCTGCCCCCACACTTTGCCTCAAATCAT

CAGTGTGCGGCTGTTGGTACGGCTTCGTCATACTGAGGGGCGCATTTTCACCCCTCGCTCTGGATTTTCCAT
TTTCAGTGCGGGTGCGGGGTGCGCTTATCAGGGAGCTTATCTCCTACAC-
CAAAACCCTGCTGTAACAACAACTCACTGCCGCTACTACCACCACTTGATTCACACTCAAACCCCATCGAC
ATCCTTGAAT--
CGCTCAAGTAAATGGCCGCGAAAATCATGCTGACTCTTTTCCTACACAGCCGCCGAGCTTGGTAAGG--------
----
GGTGCTCTTTCTGGTGCGTCCTAAGTCCACACCGCGACGCGCGACAATACGACCTCGAATCATCGTTACTGA
CCTCGACTC-
TCAGGCAAACCATCTCTGGCGAGCACGGCCTCGACAGCAATGGCGTGTATGCACCTCCTATTCCCTACCTCC
AGATCTCGTCCTCCCTGCCGGCTTGGCACTGACAATCATGCAGTTACAACGGCACTTCCGAGCTTCAGCTCG
AGCGCATGAACGTCTACTTCAACGAGGTAAGTCAACAGCCACGTCG-----
TTGATCATCTGCAGCACGATTTGCTGCCGTCGCCGGGGCCTTGCTAACGCGTTATCGCCCAGGCCTCCGGCA
ACAAGTATGTCCCTCGCGCCGTCCTCGTCGATCTCGAGCCCGGTACCATGGACGCCGT

>MW477883.1 Diaporthe zaofenghuang strain TZFH1

CCCTTTGTGAACTTATACCTTA-CTGTTGCCTCGGCG-CTAGCTGGTCCTC--CGGGGCCCCTC-----
GAGGAGCAGGCACGCCGGCGGCCAAGTTAACTCTTGTTTTTACACTGAAACTCTGAGATAAAACATAAATG
AATCAAAACTTTCAACAACGGATCTCTTGGTTCTGGCATCGATGAAGAACGCAGCGAAATGCGATAAGTAA
TGTGAATTGCAGAATTCAGTGAATCATCGAATCTTTGAACGCACATTGCGCCCTCTGGTATTCCGGAGGGC
ATGCCTGTTCGAGCGTCATTTCAACCCTCAAGCCTGGCTTGGTGATGGGGCACTGCTTCTCAGGAAGCAGG
CCCTGAAATTCAGTGGCGAGCTCGCCAGGACCCCGAGCGCAGTAGT---TAAACCCTCGC-
TCTGGAAGGCCCTGGCGGTGCCCT-GCCGTTAAACCCCCCAA-
CTTCTGAAAATTTGACCTCGGATCAGGTAGGAATACCCGCTGAACTTAAGCATA-------GGGGGTA------
ACCACAAT-
CTCGGACATGCTATCCTGCCCTCCTACTGCACCTCAAATCATCAGCCCGCAGCTGCTCGGGCGGCTTCGTCA
TATCGGGGGGCGCATTTTCACCCCTCGCTTTGGATTTTCCATTTTCAGTGCGGGTGCGGGGTGCGCTTATCA
GGGGGCTTATCTCCTACACCCAAAACCCTGTTGCATCACTCACC-
CTTCTAACCACCCCCACCAATACGCTCATCATGAA-TTCCATCG-CCTCCTTCAATAACC-
GTTCGCAAAACGCGTTGAATTCCATGCTGACTTTCTATTTCCACAGCCGCCGAGCTTGGTAAGGGTC---------
---GCGTGATGTAGC--CGAGCT-CA-
GTGTACCGCCGCGACGCTCGACACGCGACAATACGACCTCGAAGCATCGTTACTGACCTCGAATTCTTAGG
CAAACCATCTCTGGCGAGCACGGCCTCGACAGCAATGGCGTGTATGCACCTCCTATTCCCTGCCCACTGATC
TTGGCCTCTCTTCCGGCTTGGCACTGACAATTGCACAGTTACAACGGCACTTCTGAGCTCCAGCTCGAGCGC
ATGAACGTCTACTTCAACGAGGTAAGTCAACAACCACATCATCCATCCGACCATCTCCAACATGGTTTACT

GCGGTCGCCCGGACCTTGCTAACGCGTTATCGCCCAGGCCTCCGGCAACAAGTATGTTCCTCGCGCCGTCCT
CGTCGATCTGGAGCCCGGTACAATGGACGCCGT

>MW477884.1 Diaporthe zaofenghuang strain TZFH3

CCCTTTGTGAACTTATACCTTA-CTGTTGCCTCGGCG-CTAGCTGGTCCTC--CGGGGCCCCTC-----
GAGGAGCAGGCACGCCGGCGGCCAAGTTAACTCTTGTTTTTACACTGAAACTCTGAGATAAAACATAAATG
AATCAAAACTTTCAACAACGGATCTCTTGGTTCTGGCATCGATGAAGAACGCAGCGAAATGCGATAAGTAA
TGTGAATTGCAGAATTCAGTGAATCATCGAATCTTTGAACGCACATTGCGCCCTCTGGTATTCCGGAGGGC
ATGCCTGTTCGAGCGTCATTTCAACCCTCAAGCCTGGCTTGGTGATGGGGCACTGCTTCTCAGGAAGCAGG
CCCTGAAATTCAGTGGCGAGCTCGCCAGGACCCCGAGCGCAGTAGT---TAAACCCTCGC-
TCTGGAAGGCCCTGGCGGTGCCCT-GCCGTTAAACCCCCCAA-
CTTCTGAAAATTTGACCTCGGATCAGGTAGGAATACCCGCTGAACTTAAGCATA--------ATTTTA------
CCCACAATCCTCGGACATGCTATCCTGCCCTCCTACTGCACCTCAAATCATCAGCCCGCAGCTGCTCGGGCG
GCTTCGTCATATCGGGGGGCGCATTTTCACCCCTCGCTTTGGATTTTCCATTTTCAGTGCGGGTGCGGGGTG
CGCTTATCAGGGGGGCTTATCTCCTACACCCAAAACCCTGTTGCATCACTCACC-
CTTCTAACCACCCCCCACCAATACGCTCATCATGAA-TTCCATCG-CCTCCTTCAATAACC-
GTTCGCAAAACGCGTTGAATTCCATGCTGACTTTCTATTTCCACAGCCGCCGAGCTTGGTAAGGGTC---------
--GTGGCACGGTA-CGTGCTGCA-
GTGTACCGCCGCGACGCTCGACACGCGACAATACGACCTCGAAGCATCGTTACTGACCTCGAATTCTTAGG
CAAACCATCTCTGGCGAGCACGGCCTCGACAGCAATGGCGTGTATGCACCTCCTATTCCCTGCCCACTGATC
TTGGCCTCTCTTCCGGCTTGGCACTGACAATTGCACAGTTACAACGGCACTTCTGAGCTCCAGCTCGAGCGC
ATGAACGTCTACTTCAACGAGGTAAGTCAACAACCACATCATCCATCCGACCATCTCCAACATGGTTTACT
GCGGTCGCCCGGACCTTGCTAACGCGTTATCGCCCAGGCCTCCGGCAACAAGTATGTTCCTCGCGCCGTCCT
CGTCGATCTGGAGCCCGGTACAATGGACGCCGT

>KC343004.1 Diaporthella corylina strain CBS 121124

CCCTCTGTGAACTTATACCATA-CCGTTGCCTCGGCG-
CAGGCTGGTAGGCCCTCCTACCCCCCAGGGCGGGGAGAGAGCCGGCCGGCGGCCCTACAAACTCTTCTTCT
TATACTACCTCTCTGAGAAAAAAAACAAATGAATCAAAACTTTCAACAACGGATCTCTTGGTTCTGGCATC
GATGAAGAACGCAGCGAAATGCGATAAGTAATGTGAATTGCAGAATTCAGTGAATCATCGAATCTTTGAAC
GCACATTGCGCCCGCTGGTATTCCGGCGGGCATGCCTGTTCGAGCGTCATTTTACCCCTCAAGTTCGACTTG
GTGTTGGGGCATTACCTTCAGGAGGGTAAGCCCTGAAATAGACTGGCGGGCTCACTAACACTCCGAGCGTA
GTAGTGCATTCAACCTCGCTTCTGGACGGATTAGTTTGTTTCTTAGCCACTAAACCCCCCAATCTTTTATAA
AGTTGACCTCGGATCAGGTAGGAATACCCGCTGAACTTAAGCATAGAGAAGGAGGGTATGTACACACCATT

ATCACATCACCATCGACATCACACTCGAGCTC-------
GATCGACCGTGTGCGGTCGTGGCCTCTGCCTTGCCCACCTGACCCGTACGTTTTCGCCCTCCCACCGCA-----
CATTTTCTTTGTG-GTGCGGGGTTTGCGTCT------GCTTATCTCAGATAAACGTGACCC--
ACCCGACCGCCACCCGCCAGCCGCACCAGCACCGGCACCGGCACCAGCAAACCTCCCTGCCAACCCATCAC
ATGTCCCCTGCGTACCGT-
TGGCGAACCATGCTAATTCGTTTTCTCTACAGCTGCTGAGCTCGGTAAGGGTTTGCGTAAGTTGCTCCCTGT
CAACACCCCGCCGCCATTGCCGCGGCTGCCTGGAGGAGACACGTCTCGCAGGGTAACCAAATTGGTGCTGC
CTTCTGGTGCGTACCTCTCT--
AGCTCCGGATCTGCGCCTGCGGCGCGACACGGACCTCGATGTCCCCTCGTGGCATTGATACTGACCTCGAC
CG--
CAGGCAAACCATCTCTGGCGAGCACGGCCTTGACAGCAATGGCGTGTATGTACTGCCCATGCCCTATCCAC
CCTCCACGACGACCACGCCTCGCTCCGACTGACCACCGCGTAGCTACAACGGCACCTCCGAGCTCCAGCTC
GAGCGCATGAACGTCTACTTCAACGAGGTATGTCAATCGACGCACCCGTGGCATCGT-
GCTGCTGGCACTGCTC-
CCGCCGTCGCCCTGCTCCGACTGACGCGCTTCTTCTTAGGCCTCCGGCAACAAGTATGTCCCCCGCGCCGTC
CTCGTCGACCTCGAGCCCGGTACCATGGATGCCGT
