# Supplementary material for: A New Species of Diaporthe: Metabolite Profile and Ability to Suppress Sclerotium Rot of Sunchoke
Source: J Fungi (Basel). 2026 Oct 21;12(10):794. doi: 10.3390/jof12100794 (PMC13706414; doi:10.3390/jof12100794)
Supplement: Supplementary file 1 [file jof-12-00794-s001.zip › Supplementary Data Table S1.pdf]

Supplementary Data Table S1.

Host plants, number of samples examined, and number of endophytic fungal isolates recovered.

Host plant	No. of samples	No. of Fungal isolates
Globba winitii	1	3
Crinum Crinum asiaticum L.	2	5
Cratoxylum formosum	1	4
Typha angustifolia L.	3	9
Stemona collinsiae Craibr.	3	8
Gardenia saxatilis Geddes	2	5
Dionaea muscipula	1	3
Tiliacora triandra (Colebr.) Diels	4	9
Smilax perfoliata Lour.	3	7
Gardenia jasminoides J.Ellis	1	4
Amorphophallus paeoniifolius (Dennst.)	2	6
Phyllanthus niruri L	4	10
Acanthus ebracteatus Vahl.	4	11
Pavonia rigida (Wall. ex Mast.) Hochr.	1	4
Dioscorea alata L.	1	5
Kaempferia marginata Carey ex Roscoe	2	6
Catunaregam tomentosa (Blume ex DC.) Tirveng	2	6
Samanea saman (Jacq.) Merr.	1	4
Careya sphaerica Roxb.	1	2
Zingiber montanum (J.Koenig) Link ex A.Dietr.	2	6
Oxyceros horridus Lour.	1	2
Epipremnum aureum (Linden & André) G.S.Bunting	1	4
Oxalis corniculata L.	4	11
Ixora finlaysoniana Wall. ex G.Don	1	3
Sindora siamensis Teijsm. ex Miq.	1	4
Dioscorea esculenta (Lour.) Burkill	1	2
Chromolaena odorata (L.) R.M.King & H.Rob.	3	9
Harrisonia perforata (Blanco) Merr.	2	4
Drynaria quercifolia (L.) J. Sm.	3	6
Bauhinia scandens L.	3	8
Phyllanthus emblica L.	2	7
Aganosma marginata (Roxb.) G.Don	3	10
Memecylon edule Roxb.	3	9
**Total**	**69**	**196**
