# Supplementary material for: A New Species of Diaporthe: Metabolite Profile and Ability to Suppress Sclerotium Rot of Sunchoke
Source: J Fungi (Basel). 2026 Oct 21;12(10):794. doi: 10.3390/jof12100794 (PMC13706414; doi:10.3390/jof12100794)
Supplement: Supplementary file 1 [file jof-12-00794-s001.zip › Supplementary Data Table S2 .pdf]

Supplementary Data Table S2.

Effect of a fungal endophyte on the pathogenic fungus *S. rolfsii* using the dual culture technique.

Fungal isolate	Molecular identification	Gene/region	Percent inhibition
3.2	*Diaporthe formosum* sp. nov.	ITS, TEF1-α, TUB2	57.14 ± 1.17^{ef}
23.1	*Hypoxylon* sp.	ITS	68.57 ± 1.17^{a}
25.2	*Macrophomina phaseolina*	ITS	57.14 ± 3.50^{ef}
26.3	*Hypoxylon* sp.	ITS	65.71 ± 1.17^{ab}
31.1	*Lasiodiplodia theobromae*	ITS	61.43 ± 2.33^{cd}
32.1	*Daldinia korfii*	ITS	64.29 ± 1.17^{bc}
37.1	*Aspergillus flavus*	ITS	55.71 ± 2.33^{f}
42.1	*Lasidiopodia* spp.	ITS	62.86 ± 1.17^{bcd}
42.3	*Macrophomina* spp.	ITS	57.14 ± 2.33^{ef}
49.1	*Penicillium cataractarum*	ITS	68.57 ± 0.58^{a}
62.1	*Aspergillus oryzae*	ITS	50.00 ± 1.17^{g}
64.2	*Pyrrhoderma noxium*	ITS	60.00 ± 2.33^{de}

Values represent the mean ± SD of the percentage inhibition in the dual-culture assay between endophytic fungi and *S. rolfsii*. Values followed by the same letter are not significantly different according to the LSD test ($P > 0.05$).
