# Supplementary material for: A New Species of Diaporthe: Metabolite Profile and Ability to Suppress Sclerotium Rot of Sunchoke
Source: J Fungi (Basel). 2026 Oct 21;12(10):794. doi: 10.3390/jof12100794 (PMC13706414; doi:10.3390/jof12100794)
Supplement: Supplementary file 1 [file jof-12-00794-s001.zip › Supplementary Data Table S3 .pdf]

Supplementary Data Table S3

Proteins identified from the endophytic fungus *D. formosum* sp. nov. using LC-MS-based analysis

Protein ID	Protein name	Gene name	Organism	Number of proteins	Razor + unique peptides	Sequence coverage [%]	Score	Intensity Blank	Intensity 3.2	Q-value
A0ABR4E308	Peptidase S1 domain-containing protein	FJTKL_00426	Diaporthe vaccinii	4	1	5.8	18.712	0	8,608.00	0
A0ABR1P4J1	Xylanolytic transcriptional activator regulatory domain-containing protein	SLS63_007735	Diaporthe eres (Phomopsis oblonga)	1	1	1.7	7.7166	0	8,175.60	0
A0ABR4F8M5	Calcineurin-like phosphoesterase domain-containing protein	FJTKL_13733	Diaporthe vaccinii	1	1	5.3	7.4719	0	7,051.30	0
A0A0G2FVQ9	Putative alpha-glucosidase	UCDDA912_g02008	Diaporthe ampelina	2	1	1	6.9865	0	0	0
A0ABR1PQW9	protein disulfide-isomerase (EC 5.3.4.1)	SLS63_000476	Diaporthe eres (Phomopsis oblonga)	2	1	5.5	6.8623	0	6,490.60	0
A0A2I7ZNP4	Histone H3	his3	Diaporthe citri	1	1	29.4	6.4048	0	0	0
A0AAD9SPE8	HAUS augmin-like complex subunit 6 N-terminal domain-containing protein	N8I77_000134	Phomopsis amygdali (Fusicoccum amygdali)	1	1	2.2	6.3288	0	0	0
A0A2P5IF14	CwfJ domain-containing protein	DHEL01_v200544	Diaporthe helianthi	3	1	2.5	6.3103	0	5,542.60	0

Protein ID	Protein name	Gene name	Organism	Number of proteins	Razor + unique peptides	Sequence coverage [%]	Score	Intensity Blank	Intensity 3.2	Q-value
A0AAD9S338	MIF4G domain-containing protein	N8I77_012689	Phomopsis amygdali (Fusicoccum amygdali)	1	1	1.5	6.2925	0	0	0
A0ABR4DX74	Uncharacterized protein	FJTKL_03158	Diaporthe vaccinii	1	1	2.2	6.2782	0	9,830.30	0
A0AAD9SNV4	Uncharacterized protein	N8I77_000463	Phomopsis amygdali (Fusicoccum amygdali)	1	1	3.5	6.0705	0	0	0
A0ABR1PQS6	2EXR domain-containing protein	SLS63_000419	Diaporthe eres (Phomopsis oblonga)	1	1	3.7	6.0438	0	24,367.00	0
A0ABR3Y1Y8	PA domain-containing protein	Daus18300_000750	Diaporthe australafricana	1	1	4.4	6.034	0	4,446.10	0
A0A2P5HI60	Uncharacterized protein	DHEL01_v211666	Diaporthe helianthi	1	1	3.8	5.999	0	0	0
A0ABR3XRC9	25S rRNA (Uridine(2843)-N(3))-methyltransferase	Daus18300_002183	Diaporthe australafricana	1	1	2.2	5.9964	0	0	0
A0ABR3WDG8	Uncharacterized protein	Daus18300_009628	Diaporthe australafricana	1	1	12	5.9824	0	15,906.00	0
A0ABR4F8I7	DH domain-containing protein	FJTKL_14897	Diaporthe vaccinii	6	1	0.6	5.9693	0	9,915.30	0
A0ABR4E031	Uncharacterized protein	FJTKL_01548	Diaporthe vaccinii	1	1	1.4	5.9667	0	0	0
A0ABR4FB88	Alkaline phosphatase	FJTKL_10658	Diaporthe vaccinii	1	1	11.3	5.9583	0	0	0
A0AAD9SJX8	Uncharacterized protein	N8I77_006810	Phomopsis amygdali (Fusicoccum amygdali)	1	1	0.7	5.9525	0	0	0.054054
