# Supplementary material for: A New Species of Diaporthe: Metabolite Profile and Ability to Suppress Sclerotium Rot of Sunchoke
Source: J Fungi (Basel). 2026 Oct 21;12(10):794. doi: 10.3390/jof12100794 (PMC13706414; doi:10.3390/jof12100794)
Supplement: Supplementary file 1 [file jof-12-00794-s001.zip › Supplementary Data Table S4 .pdf]

Supplementary Data Table S4

Metabolomics profiling of the endophytic fungus *D. formosum* sp. nov. using LC-MS based analysis

Met ID	RT (min)	m/z	Metabolite name	Adduct type	Ontology	Total score	S/N	Blank	3.2
2854	10.466	427.17441	low score: NCGC00380826-01![1-(3,4-dihydroxy-5-methoxyphenyl)-7-(3,4-dihydroxyphenyl)heptan-3-yl] acetate [IIN-based on: CCMSLIB00000845079]	[M+Na]+	Linear diarylheptanoids	1.819	180.6	53.61	3,344,378.25
90	0.664	96.04382	3-Hydroxypyridine	[M+H]+	Hydroxypyridines	1.795	141.8	4,694.85	1,209,069.39
256	0.969	124.03893	Nicotinic acid	[M+H]+	Pyridinecarboxylic acids	1.782	74.56	696.37	238,768.62
408	4.512	146.05968	2-hydroxyquinoline	[M+H]+	Hydroquinolones	1.745	29.58	184.79	59,379.23
385	3.147	143.03349	Kojic Acid	[M+H]+	Pyranones and derivatives	1.734	326.5	3,313.40	2,026,888.51
2218	8.106	359.15247	low score: Triptonide	[M+H]+	Oxepanes	1.727	622.4	0	10,637,415.89
2431	8.526	383.14722	low score: Duartin, Dimethyl Ether	[M+Na]+	8-O-methylated isoflavonoids	1.719	86.53	0	1,142,889.84
386	1.513	143.03358	Kojic Acid	[M+H]+	Pyranones and derivatives	1.714	377.9	1,098.54	5,992,787.15
608	1.925	174.05469	4-Quinolinecarboxylic acid	[M+H]+	Quinoline carboxylic acids	1.67	189.5	459.36	417,779.94
1834	6.303	317.10123	Byakangelicol	[M+H]+	5-methoxypsoralens	1.655	81.77	12.89	6,323,226.74
2452	9.005	385.16263	THYROTROPIN RELEASING HORMONE	[M+Na]+	Oligopeptides	1.646	71.32	25.23	1,830,466.83
3080	12.028	455.20407	Obacunone	[M+H]+	Limonoids	1.645	23.95	0	422,994.81
754	6.213	193.0493	7,8-Dihydroxy-4-methylcoumarin	[M+H]+	7,8-dihydroxycoumarins	1.636	10.03	0	422,541.02
3202	9.871	471.19965	Limonin	[M+H]+	Limonoids	1.635	14.37	0	600,439.53
1585	9.102	291.15646	NCGC00385489-01!3-[4-methyl-1-(2-methylpropanoyl)-3-oxocyclohexyl]butanoic acid [IIN-based on: CCMSLIB00000846465]	[M+Na]+	Menthane monoterpenoids	1.634	33.62	0	92,468.28
2380	6.328	377.15762	[1,3-dihydroxy-1-(7-methoxy-2-oxochromen-6-yl)-3-methylbutan-2-yl] (E)-2-methylbut-2-enoate (or Angelol D)	[M+H]+	Coumarins and derivatives	1.626	8.84	74.08	629,596.26
1990	4.386	335.10944	triandrin	[M+Na]+	Fatty acyl glycosides of mono- and disaccharides	1.623	8.64	0	833,144.37

Met ID	RT (min)	m/z	Metabolite name	Adduct type	Ontology	Total score	S/N	Blank	3.2
2488	9.337	389.15729	low score: diacetoxyscirpenol	[M+Na]+	Trichothecenes	1.618	4.25	577.6	165,481.85
276	3.024	127.03844	Maltol	[M+H]+	Pyranones and derivatives	1.615	70.97	6,852.61	1,455,888.22
1964	9.051	331.15131	low score: 8-(3-methoxy-2-(methoxycarbonyl)phenyl) octanoic acid	[M+Na]+	O-methoxybenzoic acids and derivatives	1.605	15.97	57.12	8,824.20
3054	8.272	451.1933	NCGC00385479-01_C21H32O9_	[M+Na]+	Diterpene glycosides	1.603	21.47	466.53	306,776.37
739	5.635	191.06956	NCGC00381405-01!7-hydroxy-3,5-dimethylisochromen-1-one	[M+H]+	Isocoumarins and derivatives	1.592	62.53	0	627,499.12
751	6.052	193.04897	7,8-Dihydroxy-4-methylcoumarin	[M+H]+	7,8-dihydroxycoumarins	1.592	12.54	17.26	533,308.25
1700	0.706	303.06854	low score: 4-hydroxy-1'-methylspiro[benzo[e][1,3]oxazine-2,3'-indolin]-2'-one	[M+Na]+	Benzoxazines	1.591	#####	1,046.18	200,683.66
740	6.188	191.07016	2-hydroxy-4-methyl-3H-benzo[e][1,4]diazepin-5(4H)-one	[M+H]+	1,4-benzodiazepines	1.59	74.5	0	643,258.90
3403	11.61	499.23071	low score: Dihydrogedunic Acid, Methyl Ester	[M+Na]+	Naphthopyrans	1.589	86.81	0	1,975,973.88
1858	5.98	319.11591	5-(3-furan-2-yl-acryloyl)-2,2-dimethyl-4,6-dioxo-cyclohexanecarboxylic acid methyl ester	[M+H]+	Beta-diketones	1.587	49.78	92.84	2,448,078.81
623	5.411	177.05412	trans-Ferulic acid; [M+H-H2O]+	[M+H-H2O]+	Hydroxycinnamic acids	1.586	29.88	1,371.01	210,475.93
961	8.381	219.17348	5-hydroxyculmorin	[M-2H2O+H]+	Bicyclic monoterpenoids	1.574	3	0	9,461.22
807	4.375	199.08626	4-Nitrosodiphenylamine	[M+H]+	Aniline and substituted anilines	1.567	20.9	433.55	100,033.33
3917	11.126	611.2453	low score: (1S,2R,4S,9R,10R,14S,15S,17S)-9-(furan-3-yl)-1-hydroxy-15-[(1R)-1-hydroxy-2-methoxy-2-oxoethyl]-10,14,16,16-tetramethyl-7,18-dioxo-3,8-dioxapentacyclo[12.3.1.0?,?.0?,??.0?,??]octadecan-17-yl 2-methylpropanoate	[M+Na]+	Limonoids	1.55	9.17	0	537,386.86
461	2.805	154.04971	low score: 3-AMINO-4-HYDROXYBENZOATE	[M+H]+	Hydroxybenzoic acid derivatives	1.549	4.63	394.53	251,222.91
2287	7.074	367.17252	low score: Dehydrocorydalin	[M+H]+	Protoberberine alkaloids and derivatives	1.547	14.14	161.57	22,371.79
3863	10.883	595.2514	(R)-N-((R)-1-(2-aminoacetamido)-4-methyl-1-oxopentan-2-yl)-1-(2-((3-(4-fluorophenyl)-2-	[M+H]+	Dipeptides	1.543	97.1	55.15	299,677.13

Met ID	RT (min)	m/z	Metabolite name	Adduct type	Ontology	Total score	S/N	Blank	3.2
			methyl-4-oxo-4H-chromen-7-yl)oxy)acetyl)pyrrolidine-2-carboxamide						
3204	14.862	471.27237	low score: Withanone	[M+H]+	Withanolides and derivatives	1.54	10.41	0	81,506.31
2342	9.603	373.16208	Phillygenin	[M+H]+	Furanoid lignans	1.539	17.3	0	109,849.81
2234	2.514	361.07397	low score: NCGC00347521-02!3,6,8-trihydroxy-3-methyl-2,4-dihydrobenzo[a]anthracene-1,7,12-trione [IIN-based on: CCMSLIB00000848965] (or rabelomycin)	[M+Na]+	Angucyclines	1.537	59.07	0	15,025.10
499	2.698	160.07521	Indole-3-acetaldehyde	[M+H]+	3-alkylindoles	1.533	21.57	99.94	208,819.93
2630	10.042	403.17953	low score: AVE5638	[M+H]+	Phenylpiperidines	1.522	132.4	1,886.50	11,216,635.64
3402	11.224	499.23065	low score: Dihydrogedunic Acid, Methyl Ester	[M+Na]+	Naphthopyrans	1.521	25.63	0	746,582.93
624	6.028	177.05435	low score: 7-Hydroxy-4-methylcoumarin	[M+H]+	7-hydroxycoumarins	1.521	6.94	443.21	537,443.38
3999	10.008	637.2995	low score: Thalsimine	[M+H]+	Tannins	1.512	24.26	0	172,179.17
2908	7.37	433.14566	low score: 3,5,6,7,8,3',4'-Heptamethoxyflavone	[M+H]+	8-O-methylated flavonoids	1.506	92.92	0	1,673,404.88
1857	6.912	319.11588	5-(3-furan-2-yl-acryloyl)-2,2-dimethyl-4,6-dioxo-cyclohexanecarboxylic acid methyl ester	[M+H]+	Beta-diketones	1.506	113.9	498.9	4,540,557.64
573	5.821	169.08916	low score: 2-(3-oxobutyl)cyclopentane-1,3-dione	[M+H]+	Beta-diketones	1.503	109.1	53.92	7,786,521.53
1358	5.614	265.09717	Peucedanol	[M+H]+	7-hydroxycoumarins	1.501	38.58	2,547.95	698,261.77
94	1.568	97.02825	low score: Propionic acid	[M+Na]+	Carboxylic acids	1.501	9.37	1,168.21	212,199.59
2119	5.244	349.12534	low score: Citrusin	[M+Na]+	Phenolic glycosides	1.49	3.37	0	455,368.04
3038	7.34	449.17767	low score: NCGC00385793-01_C21H30O9_Azuleno[4,5-b]furan-2(3H)-one, 8-(beta-D-glucopyranosyloxy)decahydro-6-hydroxy-6-methyl-3,9-bis(methylene)-, (3aS,6R,6aR,8S,9aR,9bS)-	[M+Na]+	Guaianolides and derivatives	1.487	6.03	0	547,833.95
625	8.01	177.0544	low score: 4-Methylumbelliferone	[M+H]+	7-hydroxycoumarins	1.486	5.39	0	296,312.20

Met ID	RT (min)	m/z	Metabolite name	Adduct type	Ontology	Total score	S/N	Blank	3.2
1600	7.857	293.13901	low score: NCGC00179952-02_C16H20O5_2H-3-Benzoxacyclododecin-2,10(1H)-dione, 4,5,6,7,8,9-hexahydro-11,13-dihydroxy-4-methyl- (or (S)-Curvularin)	[M+H]+	Macrolides and analogues	1.478	94.94	113.21	7,549,208.80
2233	0.715	361.0737	low score: NCGC00347521-02!3,6,8-trihydroxy-3-methyl-2,4-dihydrobenzo[a]anthracene-1,7,12-trione [IIN-based on: CCMSLIB00000848965]	[M+Na]+	Angucyclines	1.477	60.57	0	14,022.83
1359	5.884	265.14096	low score: 4-epivulgarin	[M+H]+	Eudesmanolides, secoeudesmanolides, and derivatives	1.465	64.48	597.48	205,848.40
673	10.098	183.0294	low score: 1-Methyluric acid	[M+H]+	Xanthines	1.463	38.78	547.29	5,219,228.39
1601	6.028	293.13925	low score: curvularin	[M+H]+	Macrolides and analogues	1.462	98.26	266.82	7,438,689.11
2490	10.827	389.19324	low score: quassin	[M+H]+	Quassinoids	1.458	14.28	0	541,623.84
752	7.831	193.04915	low score: 4-Methyl-6,7-dihydroxycoumarin (or 4-Methylesculetin)	[M+H]+	6,7-dihydroxycoumarins	1.456	8.76	0	427,243.66
2354	3.032	375.08908	low score: NCGC00169972-02!3,6-dihydroxy-8-methoxy-3-methyl-2,4-dihydrobenzo[a]anthracene-1,7,12-trione [IIN-based on: CCMSLIB00000847976] (or 8-O-Methylrabelomycin)	[M+Na]+	Angucyclines	1.449	31.15	0	1,934.59
3025	7.814	447.16214	low score: NCGC00384773-01_C21H28O9_2-Hydroxycyclohexyl 2-O-[(2E)-3-(4-hydroxyphenyl)-2-propenoyl]-beta-D-glucopyranoside (or Purpurein)	[M+Na]+	Coumaric acid esters	1.449	10.16	48.87	444,856.62
113	2.653	100.07513	low score: 2-Piperidone	[M+H]+	Piperidinones	1.442	26.47	1,344.72	166,574.78
2782	7.546	419.13208	low score: liquiritin	[M+H]+	Flavonoid O-glycosides	1.439	33.56	0	1,011,396.04
518	2.912	162.05437	low score: 2,8-Quinolinediol	[M+H]+	Quinolones and derivatives	1.438	8.64	0	123,911.97
675	6.213	183.03084	low score: 1-Methyluric acid	[M+H]+	Xanthines	1.435	115.1	191.87	10,460,963.45
1701	1.184	303.06873	low score: 4-hydroxy-1'-methylspiro[benzo[e][1,3]oxazine-2,3'-indolin]-2'-one	[M+Na]+	Benzoxazines	1.434	#####	0	117,313.81
1818	8.526	315.12299	low score: 4'-hydroxy-2',4,6'-trimethoxychalcone	[M+H]+	Cinnamylphenols	1.433	32.75	12.24	613,663.53

Met ID	RT (min)	m/z	Metabolite name	Adduct type	Ontology	Total score	S/N	Blank	3.2
2869	11.224	429.18945	low score: Cratoxylone	[M+H]+	8-prenylated xanthones	1.433	19.75	0	247,662.97
1819	10.925	315.15643	low score: NCGC00384680-01_C17H24O4_Bicyclo[7.2.0]undec-4-en-3-one, 11-[(acetyloxy)methyl]-4-(hydroxymethyl)-11-methyl-8-methylene-, (1R,4Z,9S)-	[M+Na]+	Sesquiterpenoids	1.429	27.3	15.06	13,190.89
2363	7.72	375.14243	low score: Rubone	[M+H]+	2'-Hydroxychalcones	1.417	57.23	0	2,392,372.97
1855	5.499	319.07904	low score: lecanoric acid	[M+H]+	Depsides and depsidones	1.416	28.74	0	1,516,798.52
1247	6.102	253.17908	low score: Walleminone	[M+H]+	Sesquiterpenoids	1.415	5.71	65.2	20,342.85
626	8.272	177.05455	low score: 7-Hydroxy-4-methylcoumarin (or 4-Methylumbelliferone)	[M+H]+	7-hydroxycoumarins	1.414	5.58	0	258,142.09
2175	7.043	355.11508	low score: L-Asarinin	[M+H]+	Furanoid lignans	1.413	13.68	0	357,703.08
3617	8.548	539.19019	low score: NCGC00380700-01_C27H32O10_Methyl (2S)-[(5S,6S,10S,12S,13R,15S,19S)-6-(3-furyl)-15,18-dihydroxy-1,5,16-trimethyl-8,14-dioxo-7,11-dioxahexacyclo[14.2.1.0~2,12~.0~5,10~.0~10,12~.0~13,18~]nonadec-19-yl](hydroxy)acetate	[M+Na]+	Prostaglandins and related compounds	1.411	15.52	0	225,895.03
3037	6.507	449.14133	low score: Bruceine H	[M+Na]+	Quassinoids	1.408	5.6	0	442,151.85
1962	6.096	331.11661	low score: NCGC00381075-01!3-(2,4-dihydroxypentyl)-6,8-dimethoxyisochromen-1-one [IIN-based on: CCMSLIB00000847759]	[M+Na]+	Isocoumarins and derivatives	1.404	149.4	0	6,715,968.74
1079	10.925	233.15279	low score: (+)-Costunolide	[M+H]+	Germacranolides and derivatives	1.402	78.09	124.48	24,159.62
1794	8.902	313.10495	low score: 5,7-dimethoxy-3-(4-methoxyphenyl)-4H-chromen-4-one (or 4',5,7-Trimethoxyisoflavone)	[M+H]+	7-O-methylisoflavones	1.395	23.87	0	433,518.48
887	6.164	209.08102	low score: Sinapoyl aldehyde	[M+H]+	Methoxyphenols	1.39	13	0	550,934.09
1368	4.604	267.08371	low score: 5-(2-oxohexahydro-1H-thieno[3,4-d]imidazol-4-yl)pentanoic acid	[M+Na]+	Biotin and derivatives	1.388	11.49	20.5	36,096.37
1327	6.213	261.11493	low score: 8-Deoxy-lactucin	[M+H]+	Gamma butyrolactones	1.38	87.25	866.49	11,082,079.43
883	4.146	209.07768	low score: Ethyl caffeate	[M+H]+	Coumaric acids and derivatives	1.375	#####	70.92	34,198.86

Met ID	RT (min)	m/z	Metabolite name	Adduct type	Ontology	Total score	S/N	Blank	3.2
1702	7.34	303.08411	low score: 5,7-dihydroxy-2-(3-hydroxy-4-methoxyphenyl)-2,3-dihydro-4H-chromen-4-one (or hesperetin)	[M+H]+	4'-O-methylated flavonoids	1.374	42.84	0	1,355,021.92
173	2.852	109.02914	low score: Catechol	[M-H]-	Catechols	1.847	76.77	6,963.82	707,333.17
223	0.701	117.01891	Succinic acid	[M-H]-	Dicarboxylic acids and derivatives	1.815	17.48	3,024.26	1,528,344.88
224	1.267	117.01905	Succinic acid	[M-H]-	Dicarboxylic acids and derivatives	1.806	14.42	3,336.60	2,973,543.24
315	4.418	131.07268	2-Hydroxyisocaproic acid	[M-H]-	Hydroxy fatty acids	1.786	512.2	2,085.53	4,792,176.61
614	3.742	175.0609	2-Isopropylmalic acid	[M-H]-	Hydroxy fatty acids	1.779	264.2	208.94	227,356.64
24	1.247	73.02937	Propionic acid	[M-H]-	Carboxylic acids	1.769	77.98	1,059.81	107,212.66
544	4.952	165.05515	3-Phenyllactic acid	[M-H]-	Phenylpropanoic acids	1.763	16.48	5,050.91	1,079,275.16
248	3.669	123.04465	3-Methylcatechol	[M-H]-	Catechols	1.752	8.11	987.01	808,265.71
661	3.322	181.05118	4-Hydroxyphenyllactic acid	[M-H]-	Phenylpropanoic acids	1.735	84.09	2,964.03	1,225,958.38
329	0.724	133.01358	D-(+)-Malic acid	[M-H]-	Beta hydroxy acids and derivatives	1.727	68.15	1,010.90	550,377.42
420	4.952	147.04462	low score: trans-Cinnamic acid	[M-H]-	Cinnamic acids	1.723	106.8	1,513.43	751,466.67
222	5.794	117.0185	Erythronolactone	[M-H]-	Gamma butyrolactones	1.717	63.43	690.35	143,235.53
31	1.927	75.00887	low score: Glycolic acid	[M-H]-	Alpha hydroxy acids and derivatives	1.71	12.53	0	142.87
309	5.549	131.03394	Methylsuccinic acid	[M-H]-	Methyl-branched fatty acids	1.697	52.65	1,465.68	328,543.37
642	4.004	179.03412	Caffeic acid	[M-H]-	Hydroxycinnamic acids	1.691	30.83	2,498.01	629,394.39
1060	10.348	233.15569	low score: (4S,5Z,6S)-4-(2-methoxy-2-oxoethyl)-5-[2-[(E)-3-phenylprop-2-enoyl]oxyethylidene]-6-[(2S,3R,4S,5S,6R)-3,4,5-trihydroxy-6-(hydroxymethyl)oxan-2-yl]oxy-4H-pyran-3-carboxylic acid (or jasminoside)	[M-H]-	Eremophilane, 8,9-secoeremophilane and furoeremophilane sesquiterpenoids	1.69	244.8	0	2,580,841.13
249	3.026	123.04476	3-(Hydroxymethyl)phenol	[M-H]-	Benzyl alcohols	1.677	19.53	124.66	1,301,401.83
227	3.124	117.0554	DL-2-Hydroxyvaleric acid	[M-H]-	Hydroxy fatty acids	1.676	9.41	1,059.55	1,211,576.78
636	3.856	178.08704	N-acetyltyramine	[M-H]-	1-hydroxy-2-unsubstitute	1.674	79.64	319.28	1,089,036.21

Met ID	RT (min)	m/z	Metabolite name	Adduct type	Ontology	Total score	S/N	Blank	3.2
					d benzenoids				
1408	10.728	273.14944	(4aS)-6,7-dihydroxy-1,1,4a-trimethyl-3,4,10,10a-tetrahydro-2H-phenanthren-9-one (or Nimbidiol)	[M-H]-	Diterpenoids	1.671	72.89	528.88	1,035,305.77
331	5.452	133.01384	D-(+)-Malic acid	[M-H]-	Beta hydroxy acids and derivatives	1.667	3.37	323.72	120,951.93
348	3.322	135.04559	PHENYLACETIC ACID	[M-H]-	Benzene and substituted derivatives	1.661	47.35	1,454.91	1,006,366.34
1199	6.493	249.15198	2-[(1S,2S,4aR,8aS)-1-hydroxy-4a-methyl-8-methylidene-1,2,3,4,5,6,7,8a-octahydronaphthalen-2-yl]prop-2-enoic acid (or NCGC00384653-01)	[M-H]-	Eudesmane, isoeudesmane or cycloeudesmane sesquiterpenoids	1.658	147.9	395.81	4,284,589.88
164	4.483	107.04999	BENZYL ALCOHOL	[M-H]-	Benzyl alcohols	1.65	181.2	492.24	845,010.77
172	2.504	109.02898	Catechol	[M-H]-	Catechols	1.633	9.59	1,286.69	289,891.99
498	4	159.06421	3-Methyladipic acid	[M-H]-	Medium-chain fatty acids	1.629	61.58	226.51	353,841.49
764	6.804	195.06526	Divaric acid	[M-H]-	Hydroxybenzoic acid derivatives	1.628	7.26	705.1	465,562.62
1332	9.232	265.14746	(1S,8R,9R)-8-hydroxy-4-(propan-2-ylidene)-10-oxatricyclo[7.2.1.01,5]dodecane-8-carboxylic acid	[M-H]-	Alpha hydroxy acids and derivatives	1.627	9.45	1,623.98	4,559,246.67
1200	9.664	249.15498	2-[(1S,2S,4aR,8aS)-1-hydroxy-4a-methyl-8-methylidene-1,2,3,4,5,6,7,8a-octahydronaphthalen-2-yl]prop-2-enoic acid (or NCGC00384653-01)	[M-H]-	Eudesmane, isoeudesmane or cycloeudesmane sesquiterpenoids	1.617	170.9	1,433.42	4,022,867.22
553	2.782	167.03419	4-Hydroxymandelic acid	[M-H]-	1-hydroxy-2-unsubstituted benzenoids	1.614	58.21	1,518.39	487,128.52
1485	5.617	281.13791	low score: Octyl gallate	[M-H]-	Galloyl esters	1.607	3.88	0	1,062,004.91
1212	8.204	251.09233	Trinexapac-ethyl	[M-H]-	Vinylogous acids	1.606	7.82	21	2,100,231.79
312	7.371	131.03479	GLUTARATE	[M-H]-	Dicarboxylic acids and derivatives	1.603	27.84	266.6	194,187.01
310	2.714	131.03442	Methylsuccinic acid	[M-H]-	Methyl-branched fatty acids	1.603	17.55	1,098.03	121,720.89

Met ID	RT (min)	m/z	Metabolite name	Adduct type	Ontology	Total score	S/N	Blank	3.2
1489	7.115	281.13879	4-(2,7-dihydroxy-6-methylheptan-2-yl)-3-hydroxybenzoic acid (or NCGC00180729-01)	[M-H]-	Sesquiterpenoids	1.601	5.65	1,158.24	1,228,992.42
89	3.055	94.02969	low score: 3-Hydroxypyridine	[M-H]-	Hydroxypyridines	1.599	10.65	477.07	66,525.80
250	4.134	123.04484	3-(Hydroxymethyl)phenol	[M-H]-	Benzyl alcohols	1.586	23.15	4,260.61	801,335.56
848	9.645	205.15944	low score: 4-Octylphenol	[M-H]-	1-hydroxy-2-unsubstituted benzenoids	1.585	28.28	0	396,967.90
90	3.448	94.02971	low score: 3-Hydroxypyridine	[M-H]-	Hydroxypyridines	1.583	42.18	1,332.28	141,929.26
1811	8.422	319.15436	low score: zearalenol	[M-H]-	Macrolides and analogues	1.572	12.94	402.33	441,802.82
1577	5.686	293.10223	low score: NCGC00380353-01_C15H18O6_Pentaleno[1,6a-c]pyran-9-carboxylic acid, 1,3,4,7,7a,9a-hexahydro-4-hydroxy-4-(hydroxymethyl)-6,7-dimethyl-3-oxo-, (4R,7S,7aR,9aR)-	[M-H]-	Terpene lactones	1.566	63.43	683.29	3,051,111.73
1564	12.087	291.1228	low score: curvularin	[M-H]-	Macrolides and analogues	1.556	3.64	658.5	148,797.45
1318	6.096	263.12857	Hulupinic acid (or Abscisic acid, Umbellifolide)	[M-H]-	Vinylogous acids	1.555	33.13	55.19	2,289,590.71
1568	7.834	291.12445	curvularin	[M-H]-	Macrolides and analogues	1.554	77.86	139.06	3,659,625.80
311	1.312	131.03458	low score: Glutaric acid	[M-H]-	Dicarboxylic acids and derivatives	1.549	22.69	677.76	132,948.19
1348	4.759	267.08655	low score: Ctrinin hydrate	[M-H]-	Salicylic acid and derivatives	1.549	22.56	140.8	977,807.01
1319	9.645	263.13058	Hulupinic acid (or Abscisic acid, Umbellifolide)	[M-H]-	Vinylogous acids	1.542	76.85	0	3,759,279.48
1718	4.95	309.09915	low score: Picrotin	[M-H]-	Furopyrans	1.542	12.85	8,515.52	4,745,619.44
979	5.23	223.06158	low score: Trinexapac	[M-H]-	Vinylogous acids	1.537	208.1	471.55	2,965,325.02
407	3.24	145.04974	low score: 2-Methylglutaric acid	[M-H]-	Methyl-branched fatty acids	1.536	15.47	879.73	92,938.84
2384	3.322	385.09076	low score: b-Orcinolcarboxylic acid	[2M-2H+Na]-	Hydroxybenzoic acid derivatives	1.533	14.1	0	122,990.62
2797	10.699	429.19064	low score: (1-acetyloxy-3-hydroxy-6,8a-dimethyl-7-oxo-3-propan-2-yl-2,3a,4,8-tetrahydro-1H-azulen-4-yl) 4-hydroxybenzoate (or NCGC00380527-01)	[M-H]-	Sesquiterpenoids	1.533	12.55	326.38	718,178.68
527	7.158	163.07628	low score: 2,4,6-Trimethylbenzoic acid	[M-H]-	Benzoic acids	1.529	15.19	53.85	685,139.97

Met ID	RT (min)	m/z	Metabolite name	Adduct type	Ontology	Total score	S/N	Blank	3.2
52	1.102	85.02932	low score: 1,4-Butynediol	[M-H]-	Primary alcohols	1.526	113.8	537.28	124,148.20
1792	10.534	317.13937	low score: Zearalenone	[M-H]-	Zearalenones	1.518	70.09	98.83	1,194,097.81
2092	0.998	353.07138	low score: 6:3+6O fatty acyl hexoside	[M-H]-	Fatty acyl glycosides of mono- and disaccharides	1.509	106.5	0	2,617.22
766	5.417	195.06561	low score: 2,4,5-Trimethoxybenzaldehyde	[M-H]-	Benzoyl derivatives	1.505	7.16	503.99	578,963.27
1717	5.452	309.09766	low score: Picrotin	[M-H]-	Furopyrans	1.503	8.41	6,529.61	2,382,919.40
1835	8.141	321.13391	4,6-dihydroxy-3-(3-hydroxy-2,5-dimethylphenoxy)-5-methoxy-2,5-dimethylcyclohex-2-en-1-one	[M-H]-	p-Xylenols	1.503	32.47	335.31	955,229.53
1511	3.428	285.06073	low score: Benzoic acid + 2O, O-Pen	[M-H]-	p-Hydroxybenzoic acid alkyl esters	1.502	10.69	0	17,683.54
1196	6.115	249.14896	low score: 2-[(2S,4aR,8aS)-2-hydroxy-4a-methyl-8-methylidene-3,4,5,6,7,8a-hexahydro-1H-naphthalen-2-yl]prop-2-enoic acid (or 7-Hydroxy--costic acid)	[M-H]-	Eudesmane, isoeudesmane or cycloeudesmane sesquiterpenoids	1.502	7.34	0	382,680.39
1488	5.265	281.13834	4-(2,6-dihydroxy-6-methylheptan-2-yl)-3-hydroxybenzoic acid (or hydroxysydonic acid)	[M-H]-	Sesquiterpenoids	1.498	3.35	421.93	1,398,877.86
1075	9.645	235.134	low score: Heptylparaben	[M-H]-	p-Hydroxybenzoic acid alkyl esters	1.497	31.6	0	1,611,033.38
840	5.452	205.04979	low score: Eugenitol	[M-H]-	Chromones	1.492	14.54	140.47	279,324.05
2672	10.918	415.17468	low score: methyl (1R,4aS,9S,10S)-10-acetyloxy-5,9-dihydroxy-1,4a-dimethyl-2-oxo-7-propan-2-yl-10,10a-dihydro-9H-phenanthrene-1-carboxylate	[M-H]-	1-hydroxysteroids	1.492	13	850.21	702,317.80
659	9.15	181.0504	low score: b-Orcinolcarboxylic acid	[M-H]-	Hydroxybenzoic acid derivatives	1.491	10.84	13.09	125,800.58
2652	12.503	413.19559	low score: CHEMBL1616521	[M-H]-	Morphinans	1.479	17.12	957.08	321,560.06
1313	7.158	263.12833	low score: Hulupinic acid	[M-H]-	Vinylogous acids	1.473	10.31	721.78	493,496.27
446	6.779	151.07619	Divarinol	[M-H]-	Phenylpropanes	1.469	19.4	385.05	543,308.65

Metabolites with scores > 1.0 were matched to the reference database using MS/MS information, whereas metabolites with scores ≤ 1.0 were matched using MS_1 information only and therefore have lower confidence in

identification. All metabolites are considered putatively annotated because their identities were assigned by database matching and were not confirmed using authentic reference standards.
